# Activation of CO, Isocyanides, and Alkynes by Frustrated Lewis Pairs Based on Cp*M/N (M = Rh, Ir) Couples

**DOI:** 10.1021/acs.inorgchem.5c00332

**Published:** 2025-05-19

**Authors:** Carlos Ferrer-Bru, Joaquina Ferrer, Fernando J. Lahoz, Pilar García-Orduña, Daniel Carmona

**Affiliations:** Departamento de Química Inorgánica, 16765 Instituto de Síntesis Química y Catálisis Homogénea (ISQCH), CSIC—Universidad de Zaragoza, Pedro Cerbuna 12, 50009 Zaragoza, Spain

## Abstract

The complexes [Cp*M­(κ^3^ *N*,*N*′,*N*″-**L**)]­[SbF_6_] (Cp* = η^5^-C_5_Me_5_; M = Rh, **1**, Ir, **2**; **HL** = pyridinyl-amidine) display M/N transition metal frustrated Lewis pair reactivity toward a range of substrates containing triple bonds. Whereas the rhodium complex **1** reacts with CO yielding compound [Cp*Rh­(CO)­(κ^2^ *C*,*N*-**LCO**)]­[SbF_6_] (**3**), which contains a terminal carbonyl and a carbamoyl group, the iridium complex **2** generates compound [Cp*Ir­(κ^3^ *C*,*N*,*N*′-**LCO**)]­[SbF_6_] (**4**), which only features the carbamoyl group. Compounds **1** and **2** react with stoichiometric amounts of the isocyanides CNR (R = Cyclohexyl, *p*-C_6_H_4_(OMe), CH_2_SO_2_(*p*-Tolyl)) to give the corresponding 1,1-insertion complexes [Cp*M­(κ^3^ *C*,*N*,*N*′-**LCNR**)]­[SbF_6_] (**5–10**). Complexes containing inserted and coordinated isocyanide ligands of formula [Cp*M­(CNR)­(κ^2^ *C*,*N*-**LCNR**)]­[SbF_6_] (**11–15**) are obtained upon treating **1** and **2** with excess of the corresponding isocyanide. Compound **2** reacts with CN^*t*^Bu affording the adduct [Cp*Ir­(CN^*t*^Bu)­(κ^2^ *N*,*N*′-**L**)]­[SbF_6_] (**16**) which contains a terminal CN^*t*^Bu ligand. Complex **16** is protonated by HSbF_6_ to give [Cp*Ir­(CN^*t*^Bu)­(κ^2^ *N*,*N*′-**HL**)]­[SbF_6_]_2_ (**17**). The terminal alkynes HC≡CR (R = Ph, CO_2_Et) react with **1** and **2** rendering the alkynyl complexes **18–21**. Dimethyl acetylenedicarboxylate reacts with complex **2** to give compound **22** via the formal 1,2-addition of a basic nitrogen atom and the metal across the alkyne triple bond. The new complexes have been characterized by analytical, spectroscopic and X-ray diffraction (XRD) methods.

## Graphical abstract

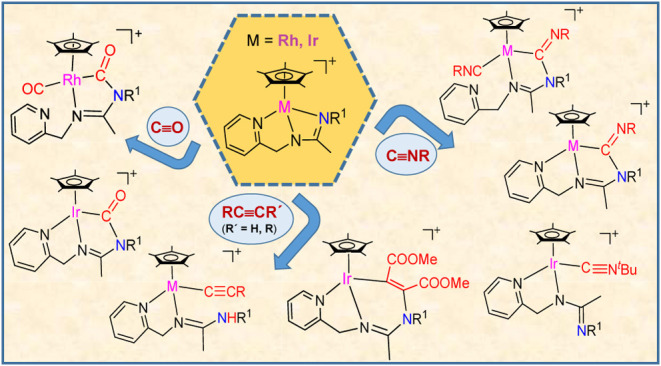

## Introduction

The initial discovery that an intermolecular or intramolecular combination of a Lewis acid and base that do not form the corresponding adduct due to geometry constraints (FLP) can activate small molecules^1^ was followed by a large number of studies introducing FLPs based on an ample variety of new acidic and basic components,^2^ including metal fragments (TMFLPs).^3^ The development of new FLPs demonstrated the effectiveness with which these species react with a wide range of small molecules (olefins, alkynes, CO_2_, SO_2_, NO, CO, N_2_O, *N*-sulfinyltolylamines etc.).^2e,2f,2h,4^ In turn, the capability of FLP chemistry to intervene efficiently in diverse fields such as homogeneous and heterogeneous catalysis including asymmetric versions,^2b–2e,3c,3d,4,5^ bioinorganic chemistry,^4^ polymers, organic chemistry,^4,6^ and materials science^2e,4^ was evidenced. In this context, a finding that substantially broadened this field was the discovery that, to exhibit FLP behavior, it is not necessary for the acidic and basic components to avoid interacting with each other. It is sufficient that an equilibrium allows access to the free acid and base for FLP reactivity to be observed.^7^

In particular, FLP chemistry has been previously applied to the activation of small molecules containing triple bonds such as carbon monoxide, isocyanides or alkynes. Regarding the activation of CO,^8^ the donor and acceptor components of conventional FLP systems capture CO following a behavior reminiscent of the σ-donation and π- acceptance of electron density characteristic of CO coordination in organometallic chemistry. Notably, the capture of CO by FLP species facilitates its reduction in the resulting adducts either stoichiometrically or catalytically.^8^ Moreover, examples are known in which the interaction of TMFLPs with CO leads to the formation of terminal metal carbonyls^9^ or carbonyl ligand insertion products,^10^ without any apparent involvement of the basic component of the FLP species in either case. As a rare example, cooperative Lewis pair chemistry has been reported for the CO activation using platinum(0) complexes as a Lewis base in conjunction with the main group Lewis acid B­(C_6_F_5_)_3_. The resulting Pt/B FLP systems led to the cooperative coupling of ethene and carbon monoxide affording the five-membered metallacycle compounds shown in Scheme 1.^11^

**Scheme 1:**
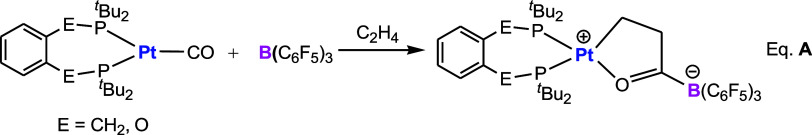
Activation of CO by TMFLPs Species

Isocyanides are important organic reagents widely used in coordination and organometallic chemistry that play an important role not only in the academic area, but also in numerous industrial processes, primarily due to their bonding properties, reactivity, and implications in organic synthesis.^12^ They exhibit reactivity toward FLPs,^13,14^ but, in general, they merely add to the acidic component of the TMFLPs, without intervention of the basic site, as it is the case of the reactions of the TMFLPs based on a Zr/P couple with butyl isocyanides shown in Scheme 2 (eqs **B** and **C**).^14^

**Scheme 2:**
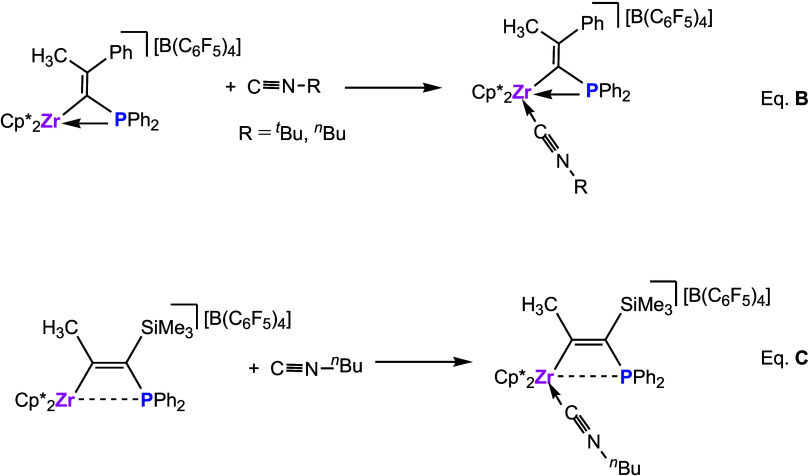
Reactions of TMFLP Species with Isocyanides

The reactivity of FLP species based on main group elements with alkynes has been extensively studied and has been the subject of recent literature reviews.^15^ This reactivity includes catalytic applications in a variety of organic transformations such as alkyne derivatization,^15^ hydrogenation^16^ or hydrosilylation.^17^ However, the chemistry of TMFLPs in this area is much less developed,^9,11a,14a,18–22^ TMFLPs based on zirconium as metal component being by far the most investigated.^9,14a,18–20^ Regarding alkynes, the most studied one is phenylacetylene,^9,11a,14a,18–20^ whose reaction with TMFLPs generally leads to metal alkynyl complexes through deprotonation.^9,11a,18,19^ Scheme 3 collects in eqs **D** and **E** two selected examples in which TMFLPs based on Zr/P and Zr/N couples react with phenylacetylene giving rise to alkynyl phosphonium^9^ and alkynyl ammonium adducts,^18^ respectively.

**Scheme 3:**
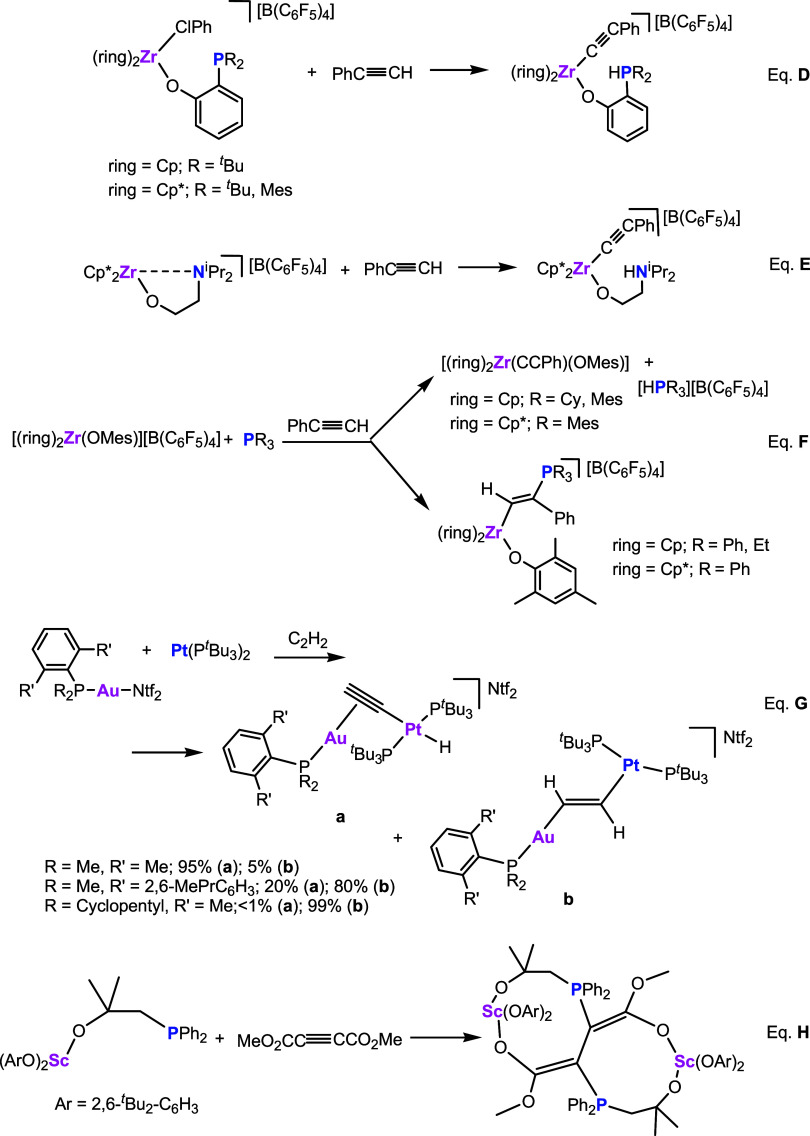
Reactions of TMFLP Species with Alkynes

However, it has been reported that the reaction of intermolecular TMFLPs, based on zirconocene aryloxide and tertiary phosphanes, with phenylacetylene can yield deprotonation products, 1,2-addition products, or mixtures of both, depending on the phosphane substituents and whether the zirconocene ring is C_5_H_5_ or C_5_Me_5_ ^20^ (Scheme 3, eq **F**). Similarly, cooperative transition metal-only frustrated Lewis pairs based on Au­(I) and Pt(0) are also able to effect deprotonation and/or FLP 1,2-addition across acetylene. Notably, subtle modifications of the phosphane ligands bound to gold have a strong effect on the regioselectivity of the activation (deprotonation vs 1,2-addition, see eq **G**).^21^

Finally, a scandium mixed alkoxyl/diaryloxide complex reacts with 0.5 mol of the internal alkyne dimethyl acetylenedicarboxylate leading to the formation of a bicyclo[7.7.0]­cetane-derived metallacycle following a double 1,4-addition pattern (eq **H**).^22^

In recent years, we have been developing a research program to study the behavior of species with stoichiometry **I** (Chart 1) in the activation processes of small molecules as well as in catalytic organic transformations. Compounds **I** are half-sandwich complexes of rhodium­(III), iridium­(III), ruthenium­(II), or osmium­(II) with phosphanoguanidine, phosphano-thiourea, pyridinyl-guanidine, and pyridinyl-amidine tridentate ligands coordinated in a *fac* κ^3^ manner. These compounds can be considered masked FLP because, in all cases, the steric strain of the four-membered M–N^1^–C–N^2^ ring makes the dissociated species **II**, containing free acceptor (metal) and donor (N^2^ atom) sites, accessible in solution under mild conditions. Indeed, following FLP reactivity pathways, they activate a variety of small molecules and mediate some catalytic reactions.^23^

**Chart 1:**
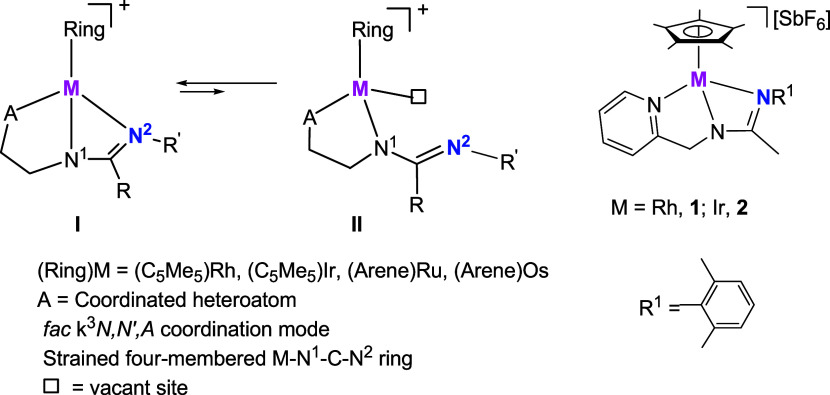
Masked FLPs Studied in This Work and Their Derived Active Species

In this work, we present the results obtained by applying the masked rhodium and iridium FLPs **1** and **2** containing a pyridinyl-amidine ligand (Chart 1) to the activation of carbon monoxide, isocyanides, and alkynes.

## Results and Discussion

### Reaction with Carbon Monoxide

Reaction of complexes [Cp*M­(κ^3^ *N*,*N′*,*N*″-**L**)]­[SbF_6_] (Cp* = η^5^-C_5_Me_5_; M = Rh, **1**, Ir, **2**; **HL** = pyridinyl-amidine ligand) with carbon monoxide gives rise to compounds [Cp*Rh­(CO)­(κ^2^ *C*,*N*-**LCO**)]­[SbF_6_] (**3**) and [Cp*Ir­(κ^3^ *C*,*N*,*N′*-**LCO**)]­[SbF_6_] (**4**) in good yields (eqs 1 and 2).
1

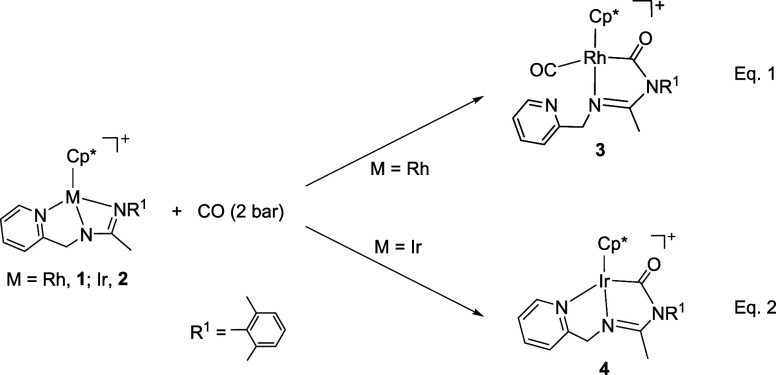




In good agreement with the structure proposed in eq 1, the IR spectrum of complex **3** shows two absorption bands at 2054 and 1688 cm^–1^ attributed to terminal and carbamoylic carbonyl groups, respectively. Two doublets in the ^13^C­{^1^H} NMR spectrum, centered at 186.17 ppm (*J*(RhC) = 74.0 Hz) and 193.66 ppm (*J*(RhC) = 33.1 Hz), indicate that both carbonyl groups are coordinated with the rhodium atom. For complex **4**, a strong IR band at 1657 cm^–1^ and a singlet at 185.30 ppm in the ^13^C­{^1^H} NMR spectrum make evident the presence of the carbamoyl group.^24^ In the formation of rhodium compound **3**, no monocarbonyl intermediate was detected by NMR. In contrast, the formation of the iridium dicarbonyl compound homologous to complex **3** has not been observed after treating **4** for 24 h under the same conditions.

The formation of the carbamoyl fragment of compounds **3** and **4** can be explained as a result of the interaction between the two components of the masked FLPs **1** and **2** with carbon monoxide: coordination of the carbonyl carbon to the metal and a nucleophilic attack by the nitrogen NR^1^ on the same carbon atom.

Molecular structure of cationic complex **3**, determined by X-ray diffraction (XRD) is depicted in Figure 1. The terminal and carbamoylic nature of the carbonyl ligands lead to the markedly different Rh–CO bond lengths, 1.9041(17) Å for the terminal group and 2.0290(16) Å for the carbamoylic one.^24a–24c^ Correspondingly, a typical CO triple bond distance (C(29)–O(2), 1.125(2) Å) was found for the terminal CO ligand while a CO double bond distance (C(11)–O(1), 1.201(2) Å) was determined for the carbamoylic one.^24a–24c^

**Figure 1:**
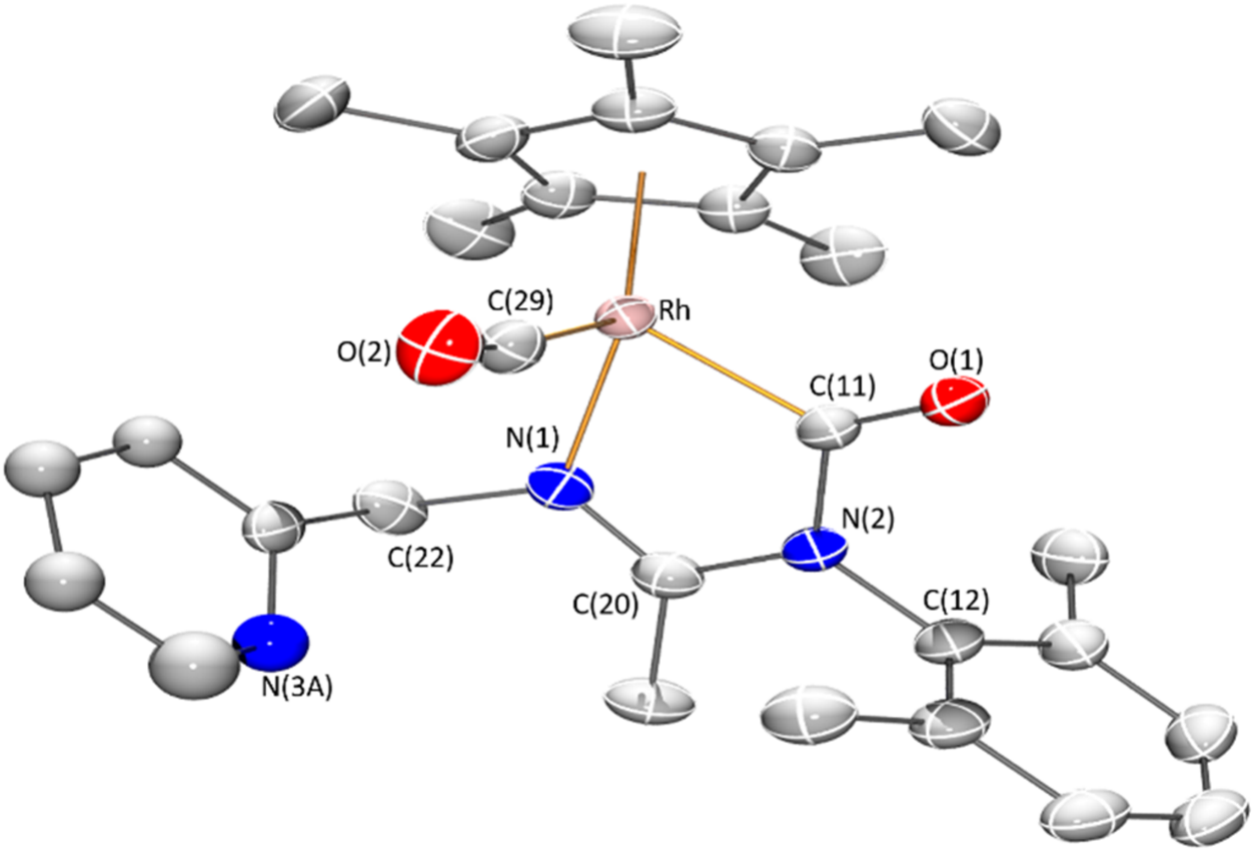
Molecular structure of the cationic complex of **3** with 50% probability ellipsoids. For clarity, hydrogen atoms and minor part of disordered pyridinic fragment have been omitted. Selected bond lengths (Å) and angles (°): Rh–Ct 1.8674(8), Rh–N(1) 2.0711(14), Rh–C(11) 2.0290(16), Rh–C(29) 1.9041(17), C(11)–N(2) 1.431(2), C(12)–N(2) 1.451(2), C(20)–N(1), 1.293(2), C(20)–N(2), 1.371(2); Ct–Rh–N(1) 129.37(5), N(1)–Rh–C(11) 78.59(6), N(1)–Rh–C(29) 95.62(7), C(11)–Rh–C(29) 92.88(7). Ct represents the centroid of the Cp* ring.

The bond distances C(20)–N(1), 1.293(2) Å, and C(20)–N(2), 1.371(2) Å, within the metallacycle Rh–N(1)–C(20)–N(2)–C(11) show π charge delocalization among the three atoms and indicate a greater double bond character for the C(20)–N(1) bond.^23a,23b^ Comparing these distances with the corresponding C–N bond distances measured in the iridium compound **2**, (1.378(6) and 1.304(6) Å, respectively)^23b^ it can be proposed that the bond order for C(20)–N(1) changes from single to double upon the CO insertion reaction. Similarly, this insertion causes the opposite change in bond order for the C(20)–N(2) bond (see eq 1). The remaining structural parameters match closely those reported for related Cp*Rh pyridinyl-amidinato complexes.^23a,23b^

### Reaction with Isocyanides

#### Reaction with CNCy, *p*-CNC_6_H_4_(OMe), and CNCH_2_SO_2_(*p*-Tolyl)

Complexes **1** and **2** rapidly reacted with one equivalent of the alkyl or aryl isocyanides CNR (R = Cyclohexyl, *p*-C_6_H_4_(OMe), CH_2_SO_2_(*p*-Tolyl)), at room temperature, to give the corresponding 1,1-insertion compounds **5**-**10** which were isolated in yields ranging from 80 to 87% (eq 3).

The new imidoyl compounds were characterized by microanalysis, mass spectrometry, IR and multinuclear one-dimensional (1D) and two-dimensional (2D) NMR spectroscopy. The IR spectrum of the products showed no bands assignable to ν­(C≡N) in the region 2200–1900 cm^–1^ but did show bands in the 1609–1559 cm^–1^ interval assignable to C–N double or partial double bonds.^25^ The ^13^C­{^1^H} NMR spectrum showed a doublet in the region 180–198 ppm with a coupling constant *J*(RhC) ≈ 45 Hz for the rhodium complexes **5**, **7**, and **9** and a singlet in the interval 167–184 ppm for the iridium complexes **6**, **8**, and **10**, tentatively attributable to the carbon atom of a coordinated M–C­(N)=N moiety.

As intense NOE interactions are observed between the hydrogens of the isocyanide group R and the methyl protons of the Cp* ligand (see Supporting Information), it is proposed that, in compounds **5**-**10**, the inserted isocyanide exhibits a *Z* configuration around its C=N double bond. This configuration was confirmed by the determination of the crystal structure of compound **8**, through X-ray diffraction (see below). The corresponding *E* isomer was not observed.

The imidoyl compounds are able to add a new molecule of isocyanide. Indeed, a dichloromethane solution of complex **6** reacts with cyclohexyl isocyanide leading to compound **11**, which contain one terminal and one inserted isocyanide molecule (eq 4). Displacement of the pyridine arm from a cyclometalated ligand by isocyanides has been previously suggested for a gold­(III) compound.^12c^ As expected, complexes **12**-**15**, congeners of **11**, could also be prepared upon treatment of dichloromethane solutions of **1** and **2** with a slight excess (3 equiv) of the corresponding isocyanide (eq 5).
5

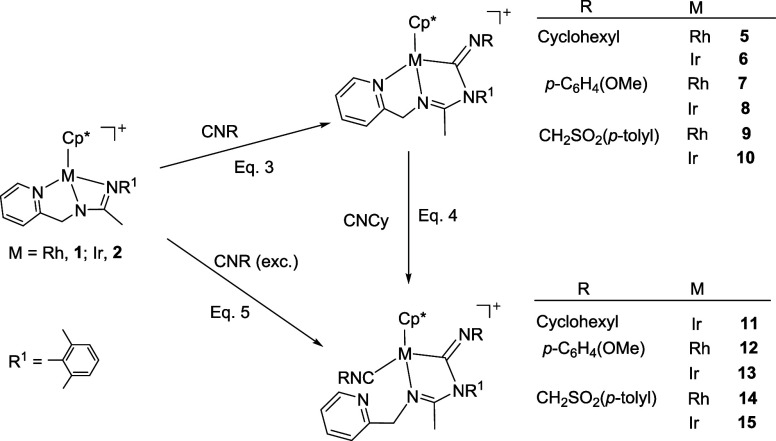




The IR spectrum of complexes **11**-**15** shows two strong bands in the regions 2182–2158 and 1624–1600 cm^–1^ characteristic of terminal and imidoyl ligands, respectively. In particular, the wavenumbers of the terminal ν­(C≡N) band are approximately 40 cm^–1^ shifted toward higher frequencies relative to the corresponding free isocyanide. This positive shift supports an almost exclusively σ-donor coordination of the employed isocyanide when coordinated with fragments of scarce π-donor capacity such as cationic Cp*M^2+^ fragments.^24d^ Additionally, the ^13^C­{^1^H} NMR spectra of the rhodium complexes **12** and **14** present two doublets at about 182 (*J*(RhC) ≈ 36 Hz) and 145 ppm (*J*(RhC) ≈ 73 Hz) which support that both CNR molecules are coordinated with the metal. For the iridium complexes **11**, **13**, and **15** the inserted and terminal isocyanide carbon atom resonate in the 172–154 and 127–114 ppm range, respectively. NOE relationship between the isocyanide R group and the Cp* methyl protons (see SI) indicate that in the formation of **11**-**15** from the corresponding imidoyl complex **6**-**10** the *Z* configuration around the C=NR double bond is retained.

#### Reaction with CN*^t^* Bu

The reaction of compounds **1** and **2** with CN^*t*^Bu gave different results compared to those shown for the isocyanides considered above. The rhodium compound **1** produced a mixture of unidentified products, even when the reaction was carried out at low temperature. In contrast, the iridium complex **2** reacts with one equivalent of CN^*t*^Bu, yielding complex **16** in nearly quantitative yield (eq 3).
6

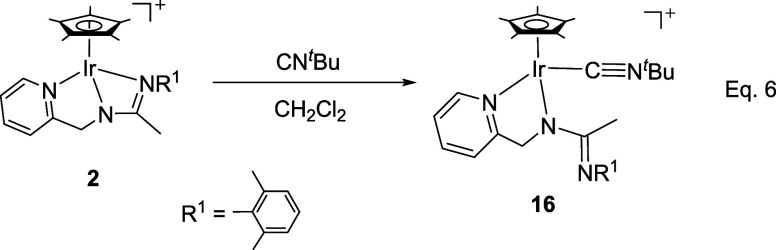




A strong absorption band centered at 2189 cm^–1^ in the IR spectrum of complex **16** along with one singlet at 116.69 ppm in the ^13^C­{^1^H} NMR spectrum point to a terminal coordination mode for the isocyanide in the complex. Again, the high shift toward higher energies of the ν­(CN) band observed in the IR spectrum (about 60 cm^–1^)^26^ support the very low π basicity of the iridium center in this compound. Strikingly, although it has been described that a Δν­(CN) value higher than 40 cm^–1^ indicates that the CNR ligand is susceptible to nucleophilic attack,^27^ the intramolecular nucleophilic addition of the hanging NR^1^ fragment of the pyridinyl amidinato ligand to the CN^*t*^Bu ligand has not been observed. It should be noted that the bulkiness of isocyanide species has been found to play a key role in controlling the insertion reactions they undergo^28^ and, therefore, the presence of the bulky *tert*-butyl substituent could be responsible for the lack of the mentioned nucleophilic addition. In this regard, NOE interactions have been observed between the methyl protons of the substituent on the amidinato ligand and the Cp* methyl protons, as well as between the methyl protons of the R^1^ group and the methylene protons of the pyridinyl-amidinato ligand (SI). These interactions suggest that in compound **16**, the bulkiness of the isocyanide ^*t*^Bu substituent forces the rotamer around the CH_2_N–C­(Me)­NR^1^ single bond of the amidinato moiety to adopt an *s-trans* conformation, with the C­(Me)=NR^1^ nitrogen pointing away from the C≡N isocyanide carbon, thereby preventing the nucleophilic attack from occurring.

The isolation and characterization of compound **16** suggest that a possible mechanism for the insertion of isocyanides into the M–NR^1^ bond of compounds **1** and **2**, leading to the formation of compounds **5**-**10** (eq 3), involves the coordination of the isocyanide with the metal after the M–NR^1^ bond breaks, followed by a nucleophilic attack of the nitrogen on the coordinated carbon of the isocyanide. However, a mechanistic pathway that, after the breaking of the M–NR^1^ bond, begins with a nucleophilic attack by the nitrogen atom on the carbon of the isocyanide, followed by the coordination of this carbon to the metal, cannot be ruled out.^12c^

Compound **16** is unstable in solution. At room temperature, in dichloromethane, it decomposes within a few hours into a mixture of unidentified products. In protic solvents, such as methanol, it quickly evolves into compounds where protonation of the uncoordinated nitrogen is detected, which led us to test the reaction of protonation of **16** with HSbF_6_. Indeed, when stoichiometric amounts of HSbF_6_ were added to dichloromethane solutions of **16**, the dicationic complex [Cp*Ir­(CN^*t*^Bu)­(κ^2^ *N,N′*-**HL**)]­[SbF_6_]_2_ (**17**) was obtained as a mixture of two isomers in a 72/28 molar ratio (eq 4).
7

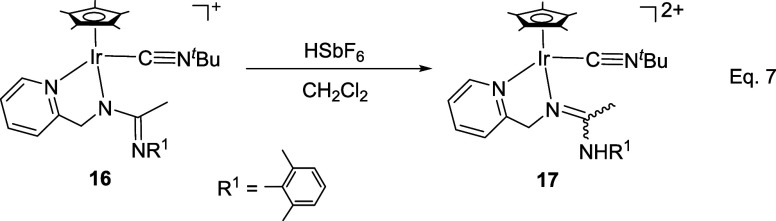




A broad IR band centered at 3355 cm^–1^ denotes the presence of an N–H functionality and, for the most abundant isomer, NOE interactions of the methyl protons of the substituent on the amidinato ligand with the Cp* methyl protons as well as with the *tert*-butyl methyl protons (SI) suggest that, the NHR^1^ group is pointing away from the isocyanide ligand.

#### Molecular Structure of the Complexes **8**, **11**, **16**, and **17**

The crystal structure of the isocyanide complexes **8**, **11**, **16**, and **17** has been elucidated by X-ray diffractometric analysis. Molecular structure of the cations are shown in Figure 2 and Table 1 collects the most relevant structural parameters. In the crystal of **17**, two independent molecules, labeled as **17a** and **17b**, were observed in the asymmetric unit with no significant differences between their structural parameters.

**Figure 2:**
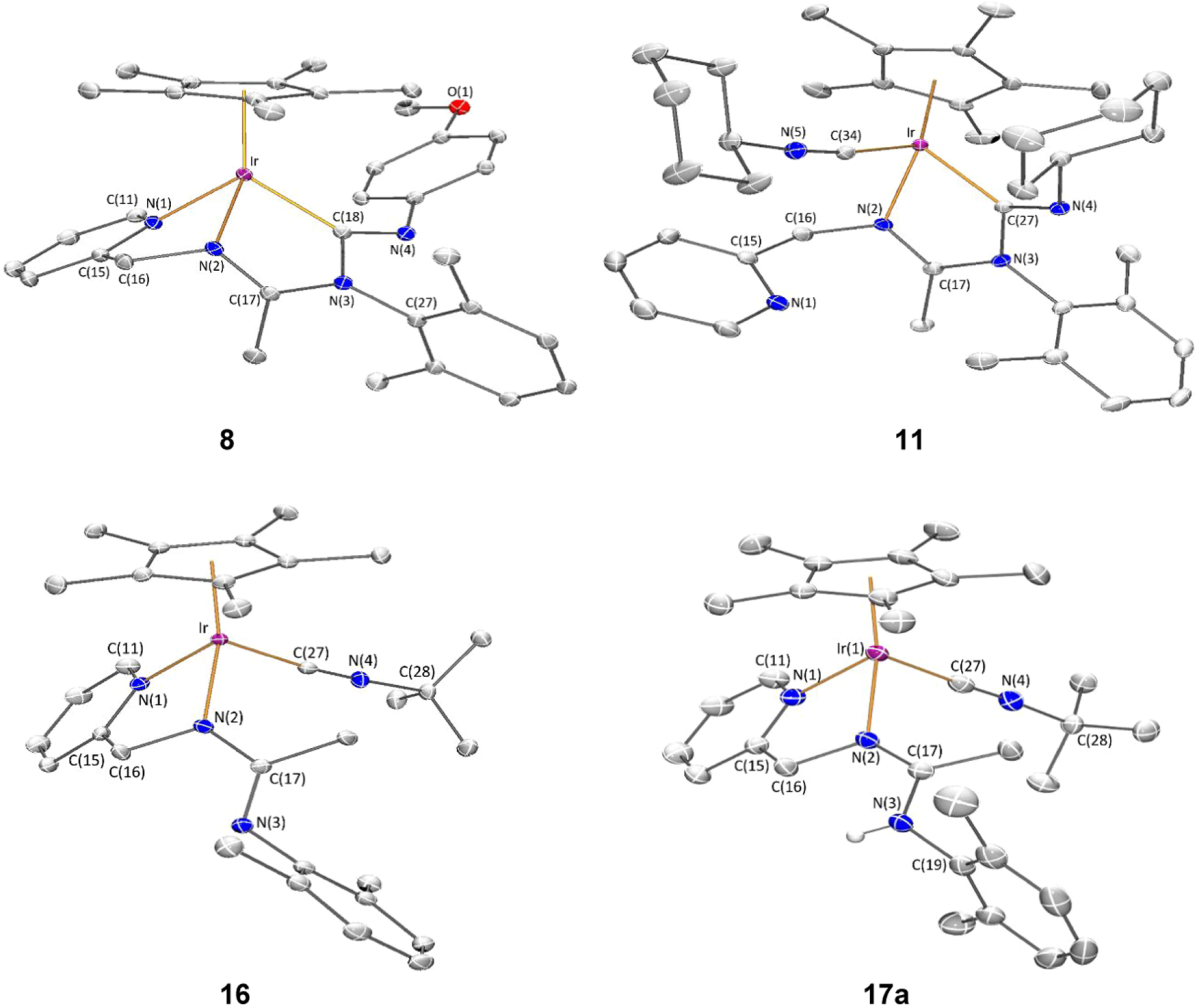
Molecular structure of the cation of the complexes **8**, **11**, **16**, and **17a** with 50% probability ellipsoids. For clarity, hydrogen atoms (except the NH proton of **17a**) have been omitted. Only one (**17a**) of the two independent cations of compound **17** is shown.

**Table 1:** Selected Structural Parameters of the Cation of the Complexes 8, 11, 16, and 17 (Bond Lengths in Å and Angles in Degrees)

Compd	Ir–CN_t_ ^b^	–C≡N	Ir–CN_i_ ^c^	>C=N	Ir–N(1)	Ir–N(2)	N(2)–C(17)	N(3)–C(17)
2^a^	--	--	--	--			1.378(6)	1.304(6)
8	--	--	2.0585(15)	1.2773(19)	2.1136(13)	2.0495(13)	1.304(2)	1.3477(19)
11	1.936(2)	1.153(3)	2.059(2)	1.267(3)	-	2.0610(16)	1.300(3)	1.352(3)
16	1.951(2)	1.155(3)	--	--	2.0914(17)	2.0879(18)	1.354(3)	1.301(3)
17a	1.970(2)	1.149(3)	--	--	2.0879(18)	2.1028(17)	1.307(3)	1.345(3)
17b	1.969(2)	1.145(3)	--	--	2.0910(19)	2.0941(17)	1.305(3)	1.341(3)

a Ref 23b.

b CN_t_ represents terminal isocyanide.

c CN_i_ represents inserted isocyanide.

All the cations exhibit the so-called three-legged piano stool geometry having a η^5^-Cp* ligand formally occupying three *fac* coordination positions. The insertion of CN­(*p*-MeOC_6_H_4_) into the Ir–NR^1^ bond of the starting compound **2** (eq 3) generates a tridentate ligand that, adopting a κ^3^ *C*,*N*,*N*′ coordination mode, occupies the three remaining vacant sites in the cation of **8**. The cation of **11** can be considered the result of the cleavage of the Ir–N­(pyridine) bond in complex **6** (homologous to **8** but with a cyclohexyl isocyanide molecule, see eq 3) followed by the coordination of a second molecule of cyclohexyl isocyanide as a terminal ligand in the created vacant site. The cation of complex **16** can be regarded as resulting from the cleavage of the Ir–NR^1^ bond in compound **2**, followed by the coordination of a CN^*t*^Bu molecule at the resulting vacant site. Protonation of the NR^1^ nitrogen atom in complex **16** leads to the formation of the dicationic compound **17**.

For terminal isocyanides, the Ir–C bond distance [from 1.936(2) Å (**11**) to 1.970(2) Å (**17a**)], as well as the C≡N bond distance [from 1.145(3) Å (**17b**) to 1.155(3) Å (**16**)], falls in the range determined for Ir­(III)–C and C≡N bond distances, respectively, in Cp*Ir­(III) terminal isocyanide complexes.^29^ Analogously, for inserted isocyanides, the Ir–C bond distance [2.0585(15) Å (**8**) and 2.059(2) Å (**11**)] is comparable to those found in Cp*Ir­(III) compounds containing inserted 2,6-xylyl isocyanide into an Ir–P bond.^29d^

Insertion of an isocyanide ligand into a metal–nitrogen bond usually leads to a charge delocalization involving the carbon and nitrogen atoms of the isocyanide ligand along with the nitrogen atom of the metal–nitrogen bond where the isocyanide has been inserted.^30^ In complexes **8** and **11**, the presence of an additional nitrogen atom located two bonds away from the nitrogen atom undergoing the insertion reaction gives the resulting CN bond in the inserted isocyanide a double-bond character, with lengths of 1.2773(19) Å in complex **8** and 1.267(3) Å in complex **11**. Instead, charge delocalization occurs between the two nitrogen atoms, N(2) and N(3), of the amidine ligand and the bonded carbon atom, C(17), with the following bond lengths: C(17)–N(2) is 1.304(2) Å in complex **8** and 1.300(3) Å in complex **11**, while C(17)–N(3) is 1.3477(19) Å in complex **8** and 1.352(3) Å in complex **11**. In the cation of the complexes **16** and **17**, where N(3) is not coordinated to the metal, a charge delocalization is also observed among the N(2), N(3), and C(17) atoms, regardless of whether N(3) is protonated or not. However, while in **8**, **11**, and **17** the N(2)–C(17) bond exhibits greater double bond character than the N(3)–C(17) bond, in **16** the opposite happens: it is the N(3)–C(17) bond that has more double bond character (Table 1).

### Reactions with Alkynes

Complexes **1** and **2** react with terminal alkynes HC≡CR (R = Ph, CO_2_Et) under mild conditions affording complexes **18–21** in which the terminal alkyne has been deprotonated (eq 5). Indeed, one strong IR band in approximately 2100 cm^–1^ and two ^13^C­{^1^H} NMR doublets (Rh) or singlets (Ir) in the regions 100–113 or 89–100 ppm, respectively, are indicative of the existence of a coordinated alkynyl group. Additionally, an IR band in the region of 3250–3290 cm^–1^ together with an ^1^H NMR broad singlet in the interval 7–8 ppm denote the presence of an NH group.
8

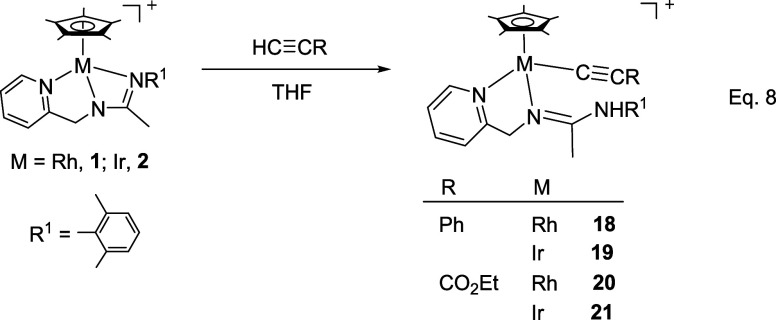




Only one isomer has been detected for compounds **18**, **20**, and **21**, but iridium compound **19** has been isolated as a mixture of two isomers in a molar ratio of 87:13. NOE measurements indicate that the most abundant isomer isolated from compound **19**, as well as the only isomer detected for compounds **18**, **20**, and **21**, is the *Z* isomer with respect to the C=N double bond of the pyridinyl amidine ligand (see SI).

On the other hand, the activated internal alkyne dimethyl acetylenedicarboxylate reacts with the iridium complex **2** affording the 1,2-addition product **22** (eq 6). The presence of two methyl ester functionalities in the product is indicated by two ν­(CO) bands, at 1725 and 1685 cm^–1^, in the IR spectrum, as well as by two singlets, at 173.67 and 164.20 ppm, in the ^13^C­{^1^H} NMR spectrum and two additional singlets, at 3.72 and 3.19 ppm, in the ^1^H NMR spectrum. All these data, together with two singlets, at 134.57 and 114.56 ppm, in the ^13^C­{^1^H} NMR spectrum, attributed to two olefinic carbon atoms, support the existence of a MeCO_2_C=CCO_2_Me group in the adduct.
9

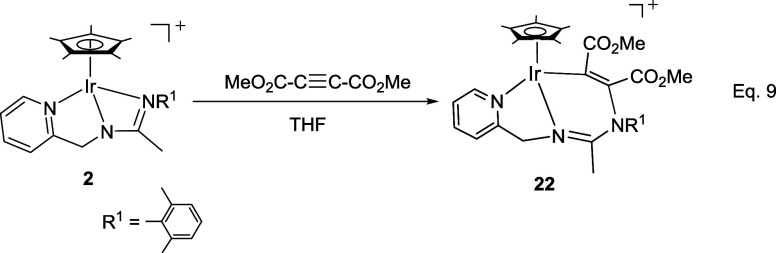




Examples of terminal alkyne activation by TMFLPs are very scarce and those of internal alkyne activation are even less abundant. Only a handful of transition metals have been investigated in these processes, zirconium being the most studied metal. Specifically, the deprotonation of terminal alkynes mediated by FLP species based on Zr/N pairs has been described, without the observation of 1,2-addition reactions to the alkyne.^18,19^ Regarding internal alkynes, as far as we know, the only example of dimethyl acetylenedicarboxylate activation by TMFLPs is the double 1,4-addition reaction of dimethyl acetylenedicarboxylate mediated by the scandium mixed alkoxyl/diaryloxide complex shown in eq **H**, Scheme 3.^22^

The proposed structure for complex **22** has been confirmed by X-ray diffractometric methods. Figure 3 shows the molecular structure of the cation together with the most relevant structural parameters. The metal exhibits a pseudo-octahedral geometry. An η^5^-Cp* group formally occupies three coordination positions, and the other three are occupied by two nitrogen atoms from the pyridinyl amidinato ligand and one carbon atom. The addition of the complex to the triple bond of the alkyne reduces the bond order of its central carbons [C(27)–C(30) 1.341(2) Å] and forms a six-membered metallacycle Ir–N(2)–C(17)–N(3)–C(30)–C(27). In the cation, this metallacycle adopts a boat conformation, with atoms Ir(1) and N(3) at the bow and stern. A certain charge delocalization is observed in the N(2)–C(17)–N(3) fragment [N(2)–C(17) 1.298(2), N(3)–C(17) 1.369(2)].

**Figure 3:**
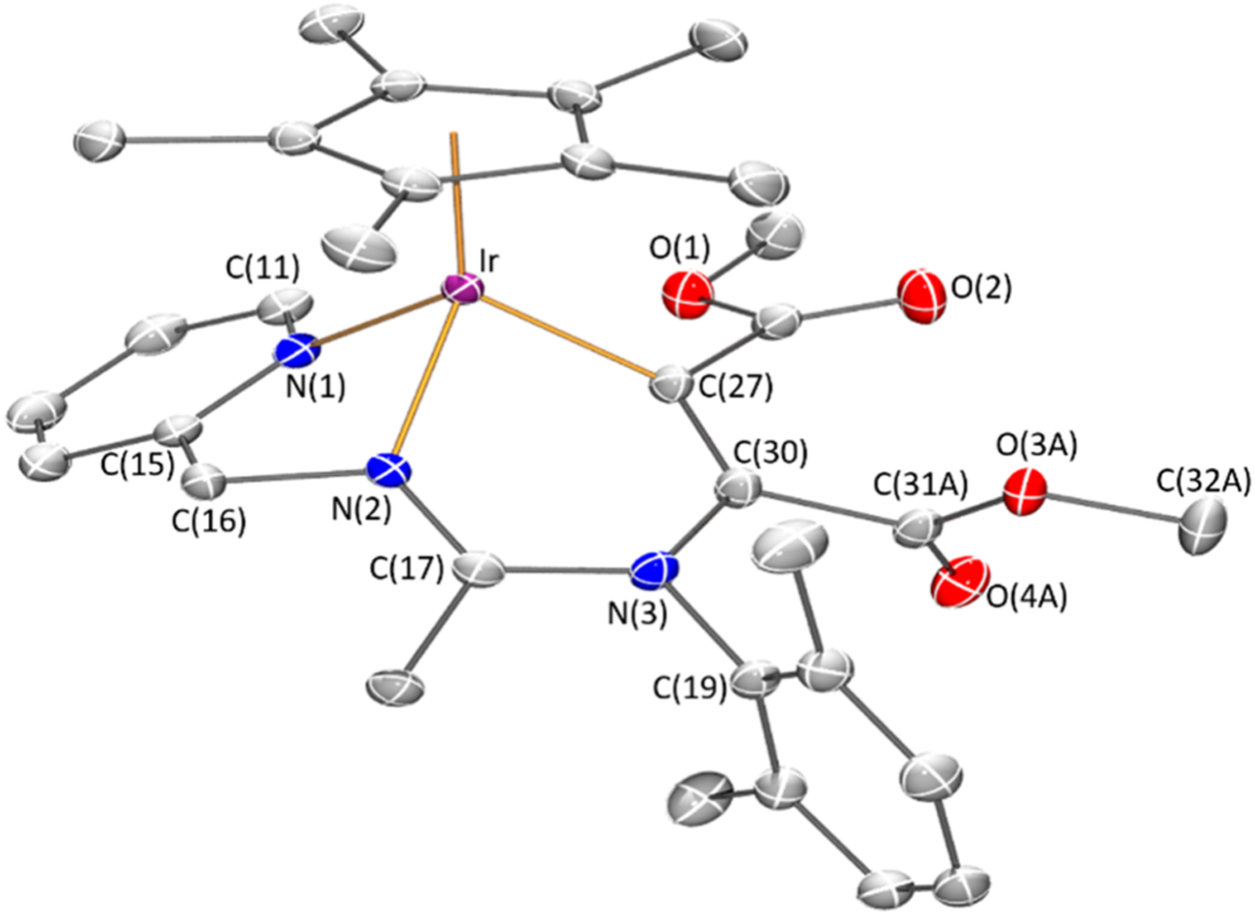
Molecular structure of the cation of complex **22** with 50% probability ellipsoids. For clarity, hydrogen atoms and the minor component of the disordered – CO_2_Me fragment have been omitted. Selected bond lengths (Å) and angles (°): Ir–Ct 1.82256(11), Ir–N(1) 2.1079(13), Ir–N(2) 2.0683(13), Ir–C(27) 2.0566(15), N(2)–C(17) 1.298(2), N(3)–C(17) 1.369(2), C(27)–C(30) 1.341(2), N(3)–C(30) 1.432(2); Ct–Ir–N(1) 126.09(4), Ct–Ir–N(2) 130.56(4), Ct–Ir–C(27) 128.15(4), N(1)–Ir–N(2) 75.13(5), N(1)–Ir–C(27) 92.24(6), N(2)–Ir–C(27) 82.87(6). Ct represents the centroid of the Cp* ring.

Finally, it should be noted that in all the new compounds described in this work, the metal is a stereogenic center and the compounds have been prepared as racemates. Regarding the crystalline structures determined by X-ray diffraction, all the compounds crystallize in centrosymmetric space groups (see SI) and, therefore, they have also been isolated as racemates. The ORTEP views of the cations shown in Figures 1, 2, and 3, correspond to the *R* at metal enantiomer for compounds **8**, **16**, **17a**, and **22** and to the *S* at metal enantiomer for compounds **3**, **11**.

## Conclusions

When compounds **1** and **2** react with molecules containing triple bonds, such as carbon monoxide, isocyanides, or alkynes, they behave like masked transition metal frustrated Lewis pairs. The use of TMFLPs in the cooperative activation of this type of molecules is very underdeveloped. This is likely due to the fact that these substrates coordinate with the metal, forming very stable terminal carbonyl, isocyanide, or alkynyl complexes,^9,10,14^ which hinders or prevents the basic part of the FLP system from participating in the process. However, in our case, it has been observed that both the acidic and its basic counterpart of the FLP are involved in all the examined reactions. As an exception, the reaction of the iridium complex **2** with CN^*t*^Bu results in the formation of complex **16**, in which only the coordination of the isocyanide as a terminal ligand has taken place (eq 3). Most likely, the steric hindrance associated with the bulky ^*t*^Bu substituent is responsible for this behavior.

In summary, compounds **1** and **2** provide a simple and easy access to powerful TMFLPs, whose electronic properties enable them to activate a variety of organic substrates. Understanding the reasons behind their activity in detail allows us to design and select new types of molecules with bonding situations that can be activated for catalytic purposes. Such tasks are currently underway in our laboratory.

## Experimental Section

### General Information

All preparations have been carried out under argon, unless otherwise stated. All solvents were treated in a PS-400–6 Innovative Technologies Solvent Purification System (SPS). Infrared spectra were recorded on a PerkinElmer Spectrum-100 FT-IR spectrometer (ATR mode). Carbon, hydrogen and nitrogen analyses were performed using a PerkinElmer 240 B microanalyzer. ^1^H and ^13^C NMR spectra were recorded on a Bruker AV-300 (300.13 MHz) or a Bruker AV-400 (400.16 MHz) spectrometers. Chemical shifts are expressed in ppm upfield from SiMe_4_; *J* values are given in Hz. COSY, NOESY, HSQC and HMBC ^1^H-X (X = ^1^H, ^13^C) correlation spectra were obtained using standard procedures. Mass spectra were obtained with a Micro Tof-Q Bruker Daltonics spectrometer.

### Preparation and Characterization of Complexes 3 and 4

At room temperature, in a sealed NMR tube, 0.10 mmol of [Cp*M­(κ^3^ *N*,*N*′,*N*″-**L**)]­[SbF_6_] (M = Rh, **1**; Ir, **2**) were dissolved in dry and oxygen-free acetone (0.5 mL). The solution was charged with CO (2 bar) and monitored by ^1^H NMR. The conversion to **3** (M = Rh) or **4** (M = Ir) was completed after 12 h or 30 min, respectively. The resulting orange (M = Rh) or yellow (M = Ir) solution was vacuum-dried affording **3** or **4** as pure compounds without further purification.

#### Compound **3**

Yield: 64.9 mg (83%). Anal. Calcd for C_28_H_33_F_6_N_3_O_2_RhSb: C, 42.99; H, 4.25; N, 5.37. Found: C, 42.72; H, 4.47; N, 5.47. HRMS (μ-TOF): C_28_H_33_N_3_O_2_Rh [M-SbF_6_]^+^: calcd 546.1622, found 546.1610. IR (cm^–1^): ν­(C≡O) 2054 (s); ν­(C=O) 1688 (m); ν­(C=N) 1620 (m), 1594 (w); ν­(SbF_6_) 653 (s).
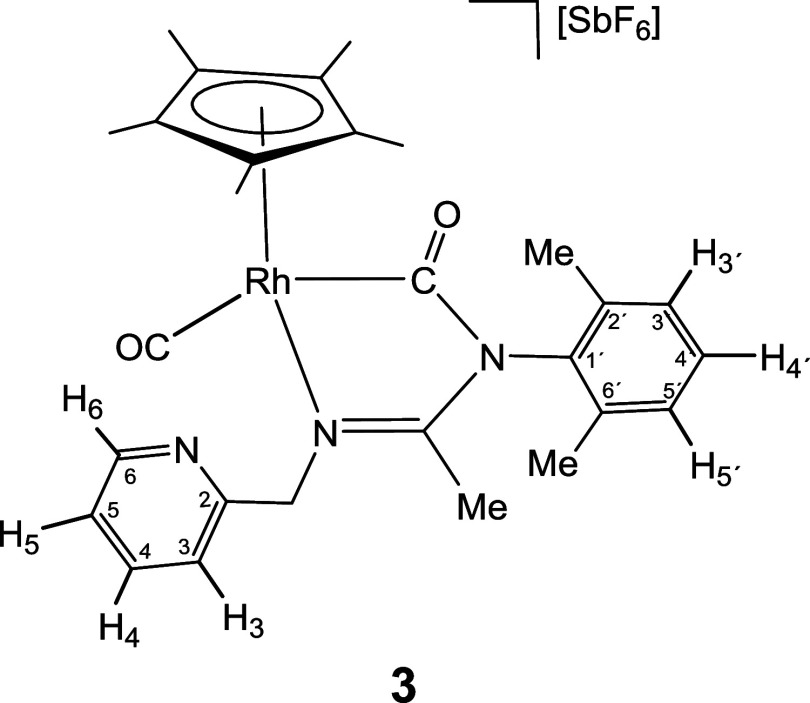


##### ^1^H NMR (300.13 MHz, THF-*d*_8_, RT, ppm)

δ = 8.51 (d, *J* = 6.1 Hz, 1H, H_6_); 7.85 (pt, 1H, H_4_); 7.53 (d, *J* = 7.7 Hz, 1H, H_3_); 7.32 (pt, 1H, H_5_); 7.29–7.14 (m, 3H, H_3′_, H_4′_, H_5′_); 5.52, 4.82 (AB system, *J*(AB) = 16.3 Hz, 1H, CH_2_); 2.16, 2.15 (2 × s, 6H, C_6_H_3_ *Me*_2_); 2.12 (s, 3H, Me); 1.94 (s, 15H, C_5_Me_5_).

##### ^13^C­{^1^H} NMR (75.48 MHz, THF-*d*_8_, RT, ppm)

δ = 193.66 (d, *J* = 33.1 Hz, Rh–C=O); 186.17 (d, *J* = 74.0 Hz, Rh–C≡O); 171.14 (C=N); 156.50 (C_2_); 150.38 (C_6_); 138.60 (C_4_); 137.56, 137.52, 137.13 (C_1′_, C_2′_, C_6′_); 130.57, 129.77, 129.70 (C_3′_, C_4′_, C_5′_); 124.35 (C_5_); 124.20 (C_3_); 108.31 (d, *J* = 4.7 Hz, *C*_5_Me_5_); 59.67 (CH_2_); 18.31, 18.13 (C_6_H_3_ *Me*_2_); 15.55 (Me); 9.44 (C_5_ *Me*_5_).

#### Compound **4**

Yield: 65.1 mg (77%). Anal. Calcd for C_27_H_33_F_6_IrN_3_OSb: C, 38.45; H, 3.94; N, 4.98. Found: C, 38.53; H, 3.98; N, 5.25. HRMS (μ-TOF): C_27_H_33_IrN_3_O [M-SbF_6_]^+^: calcd 608.2247, found 608.2231. IR (cm^–1^): ν­(C=O) 1657 (m); ν­(C=N) 1607 (m), 1594 (m); ν­(SbF_6_) 652 (s).
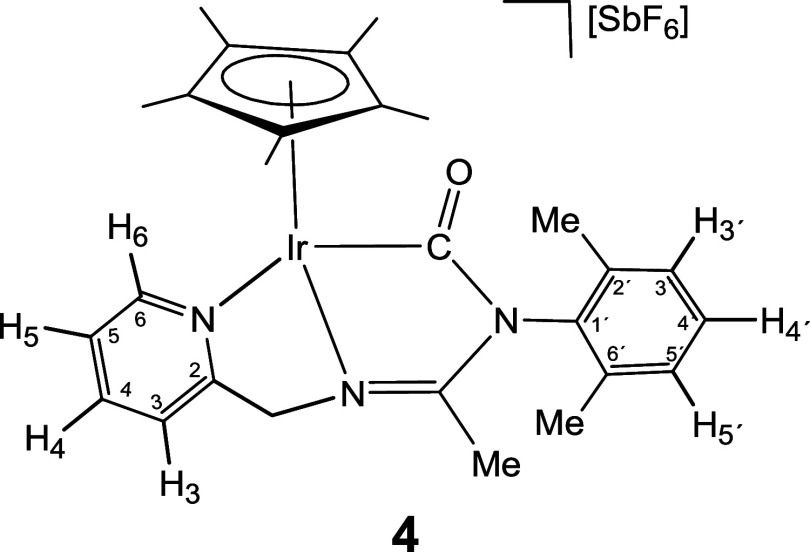


##### ^1^H NMR (300.13 MHz, THF-*d*_8_, RT, ppm)

δ = 8.59 (d, *J* = 6.4 Hz, 1H, H_6_); 8.01 (pt, 1H, H_4_); 7.84 (d, *J* = 7.8 Hz, 1H, H_3_); 7.42 (pt, 1H, H_5_); 7.23–7.00 (m, 3H, H_3′_, H_4′_, H_5′_); 5.66, 5.16 (AB system, *J*(AB) = 16.3 Hz, 2H, CH_2_); 2.15 (s, 3H, Me); 2.06, 1.61 (2 × s, 6H, C_6_H_3_ *Me*_2_); 1.75 (s, 15H, C_5_Me_5_).

##### ^13^C­{^1^H} NMR (75.48 MHz, THF-*d*_8_, RT, ppm)

δ = 185.30 (Ir–C=O); 175.22 (C=N); 166.58 (C_2_); 153.91 (C_6_); 140.95 (C_4_); 137.44, 136.84, 136.50 (C_1′_, C_2′_, C_6′_); 129.68, 129.36, 129.29 (C_3′_, C_4′_, C_5′_); 126.53 (C_5_); 123.27 (C_3_); 94.12 (*C*_5_Me_5_); 63.39 (CH_2_); 18.38, 17.95 (C_6_H_3_ *Me*_2_); 13.92 (Me); 8.91 (C_5_ *Me*_5_).

### Preparation and Characterization of Complexes **5–10**

Under argon, at room temperature, to a suspension of [Cp*M­(κ^3^ *N*,*N′*,*N*″**-L**)]­[SbF_6_] (M = Rh, **1**; Ir, **2**) (0.10 mmol) in CH_2_Cl_2_ (6 mL), 0.10 mmol of the corresponding isocyanide were added. After 30 min of stirring, the resulting solution was vacuum-concentrated until *ca*. 0.5 mL. Slow addition of diethyl ether afforded an orange (M = Rh) or yellow (M = Ir) solid which was filtered off, washed with diethyl ether (3 × 3 mL) and vacuum-dried.

#### Compound **5**

Yield: 71.8 mg (86%). Anal. Calcd for C_33_H_44_F_6_N_4_RhSb: C, 47.45; H, 5.31; N, 6.70. Found: C, 47.43; H, 5.14; N, 6.56. HRMS (μ-TOF): C_33_H_44_N_4_Rh [M-SbF_6_]^+^: calcd 599.2615, found 599.2623. IR (cm^–1^): ν­(C=N) 1609, 1585 (br); ν­(SbF_6_) 654 (s).
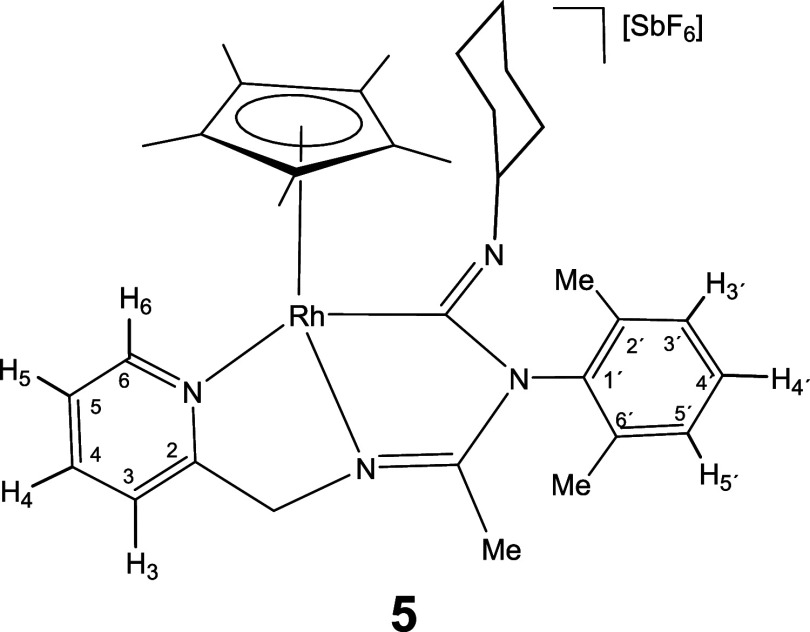


##### ^1^H NMR (300.13 MHz, CD_2_Cl_2_, RT, ppm)

δ = 8.28 (d, *J* = 7.0 Hz, 1H, H_6_); 7.90 (pt, 1H, H_4_); 7.56 (d, *J* = 7.9 Hz, 1H, H_3_); 7.41 (pt, 1H, H_5_); 7.26–6.96 (m, 3H, H_3′_, H_4′_, H_5′_); 5.10, 5.16 (AB system, *J*(AB) = 16.9 Hz, 2H, CH_2_); 3.45–3.25 (m, 1H, CH of Cy); 2.14, 1.56 (2 × s, 6H, C_6_H_3_ *Me*_2_); 1.92 (s, 3H, Me); 1.69 (s, 15H, C_5_Me_5_); 2.00–1.10 (m, 10H, CH_2_ of Cy).

##### ^13^C­{^1^H} NMR (75.48 MHz, CD_2_Cl_2_, RT, ppm)

δ = 180.79 (d, *J* = 43.8 Hz, Rh–C=N); 172.06 (C=N); 163.75 (C_2_); 152.77 (C_6_); 139.98 (C_4_); 138.84, 137.51, 135.35 (C_1′_, C_2′_, C_6′_); 128.86, 128.72 (C_3′_, C_4′_, C_5′_); 125.83 (C_5_); 122.45 (C_3_); 98.927 (d, *J* = 5.9 Hz, *C*_5_Me_5_); 66.26 (CH of Cy); 60.39 (CH_2_); 38.78, 35.39, 26.32, 25.59, 25.42 (5 × CH_2_ of Cy); 19.26, 18.46 (C_6_H_3_ *Me*_2_); 15.85 (Me); 10.27 (C_5_ *Me*_5_).

#### Compound **6**

Yield: 79.8 mg (86%). Anal. Calcd for C_33_H_44_F_6_IrN_4_Sb: C, 42.89; H, 4.80; N, 6.06. Found: C, 43.29; H, 4.59; N, 6.07. HRMS (μ-TOF): C_33_H_44_IrN_4_ [M-SbF_6_]^+^: calcd 689.3190, found 689.3206. IR (cm^–1^): ν­(C=N) 1599, 1579, 1561 (br); ν­(SbF_6_) 654 (s).
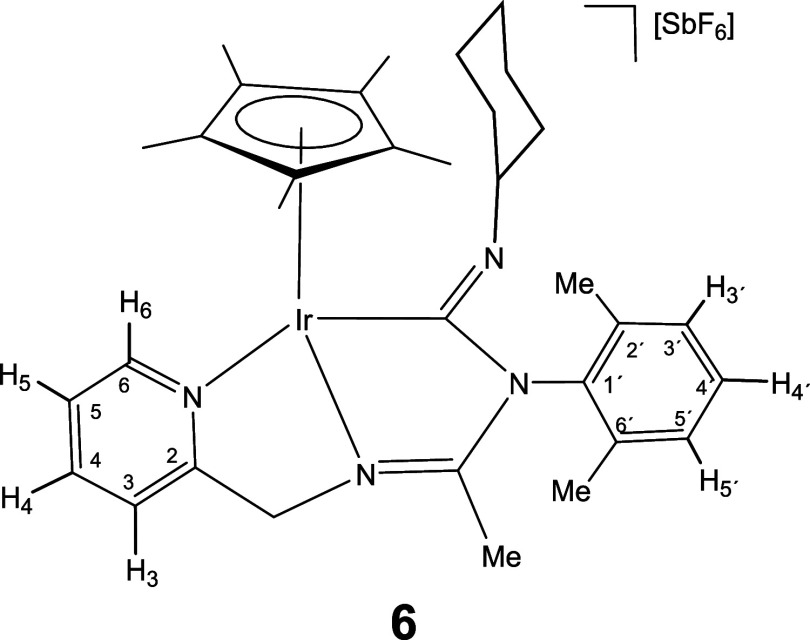


##### ^1^H NMR (300.13 MHz, CD_2_Cl_2_, RT, ppm)

δ = 8.39 (d, *J* = 6.6 Hz, 1H, H_6_); 7.90 (pt, 1H, H_4_); 7.63 (d, *J* = 7.8 Hz, 1H. H_3_); 7.35 (pt, 1H, H_5_); 7.26–7.00 (m, 3H, H_3′_, H_4′_, H_5′_); 5.28, 4.93 (AB system, *J*(AB) = 16.2 Hz, 2H, CH_2_); 3.55–3.24 (m, 1H, CH of Cy); 2.09, 1.57 (2 × s, 6H, C_6_H_3_ *Me*_2_); 1.99 (s, 3H, Me); 1.74 (s, 15H, C_5_Me_5_); 1.85–0.85 (m, 10H, CH_2_ of Cy).

##### ^13^C­{^1^H} NMR (75.48 MHz, CD_2_Cl_2_, RT, ppm)

δ = 173.09 (C=N); 167.26 (Ir–C=N); 164.78 (C_2_); 153.01 (C_6_); 139.98 (C_4_); 138.65, 137.50, 135.09 (C_1′_, C_2′_, C_6′_); 128.87, 128.68 (C_3′_, C_4′_, C_5′_); 126.09 (C_5_); 122.16 (C_3_); 91.51 (*C*_5_Me_5_); 67.04 (CH of Cy); 61.85 (CH_2_); 39.41, 35.49, 26.35, 25.55, 25.44 (5 × CH_2_ of Cy); 19.05, 18.37 (C_6_H_3_ *Me*_2_); 15.16 (Me); 10.07 (C_5_ *Me*_5_).

#### Compound **7**

Yield: 75.0 mg (87%). Anal. Calcd for C_34_H_40_F_6_N_4_ORhSb: C, 47.52; H, 4.69; N, 6.52. Found: C, 46.94; H, 4.94; N, 6.56. HRMS (μ-TOF): C_34_H_40_N_4_ORh [M-SbF_6_]^+^: calcd 623.2252, found 623.2294. IR (cm^–1^): ν­(C=N) 1598, 1583 (br); ν­(SbF_6_) 654 (s).
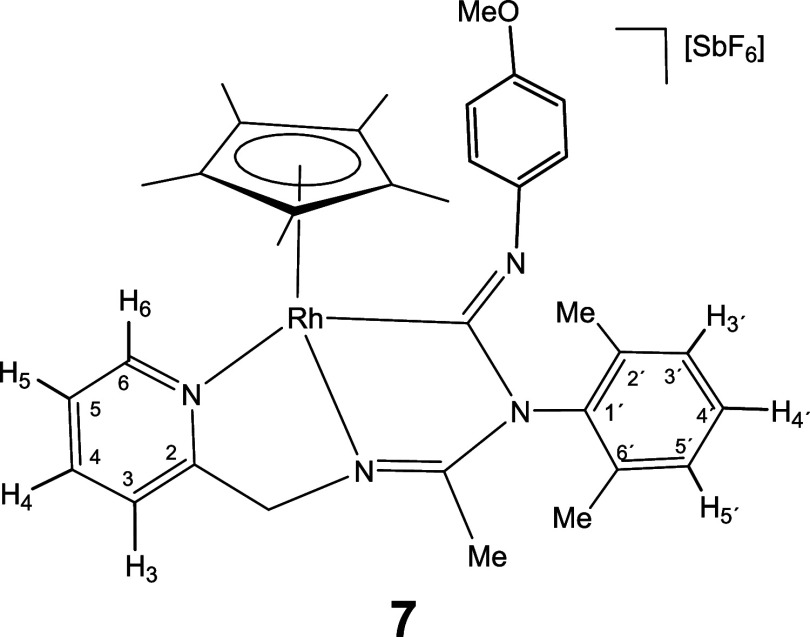


##### ^1^H NMR (300.13 MHz, CD_2_Cl_2_, RT, ppm)

δ = 8.62 (d, *J* = 5.5 Hz, 1H, H_6_); 7.98 (t, *J* = 7.7 Hz, 1H, H_4_); 7.72–7.49 (m, 2H, H_3_, H_5)_; 7.22 (d, *J* = 5.1 Hz, 2H, H_3′_, H_5′_); 7.04 (t, 1H, H_4′_); 6.95 (d, *J* = 8.8 Hz, 2H, H_Ar_); 6.71 (d, 2H, H_Ar_); 5.28, 5.22 (AB system, *J*(AB) = 16.7 Hz, 2H, CH_2_); 3.82 (s, 3H, OMe); 2.27, 1.69 (2 × s, 6H, C_6_H_3_ *Me*_2_); 2.01 (s, 3H, Me); 1.38 (s, 15H, C_5_Me_5_).

##### ^13^C­{^1^H} NMR (75.48 MHz, CD_2_Cl_2_, RT, ppm)

δ = 185.29 (d, *J* = 45.5 Hz, Rh–C=N); 171.56 (C=N); 163.79 (C_2_); 156.71 (C_Ar_); 152.75 (C_6_); 144.95 (C_Ar_); 140.21 (C_4_); 138.87, 137.09, 135.54 (C_1′_, C_2′_, C_6′_); 129.19, 129.15, 129.07 (C_3′_, C_4′_, C_5′_); 126.40 (C_5_); 123.38 (C_Ar_); 122.54 (C_3_); 114.63 (C_Ar_); 98.98 (d, *J* = 6.1 Hz, *C*_5_Me_5_); 60.17 (CH_2_); 56.08 (OMe); 19.17, 18.41 (C_6_H_3_ *Me*_2_); 15.93 (Me); 9.79 (C_5_ *Me*_5_).

#### Compound **8**

Yield: 75.9 mg (80%). Anal. Calcd for C_34_H_40_F_6_IrN_4_OSb: C, 43.05; H, 4.25; N, 5.90. Found: C, 43.07; H, 4.18; N, 5.90. HRMS (μ-TOF): C_34_H_40_IrN_4_O [M-SbF_6_]^+^: calcd 713.2831, found 713.2862. IR (cm^–1^): ν­(C=N) 1592, 1577 (br); ν­(SbF_6_) 657 (s).
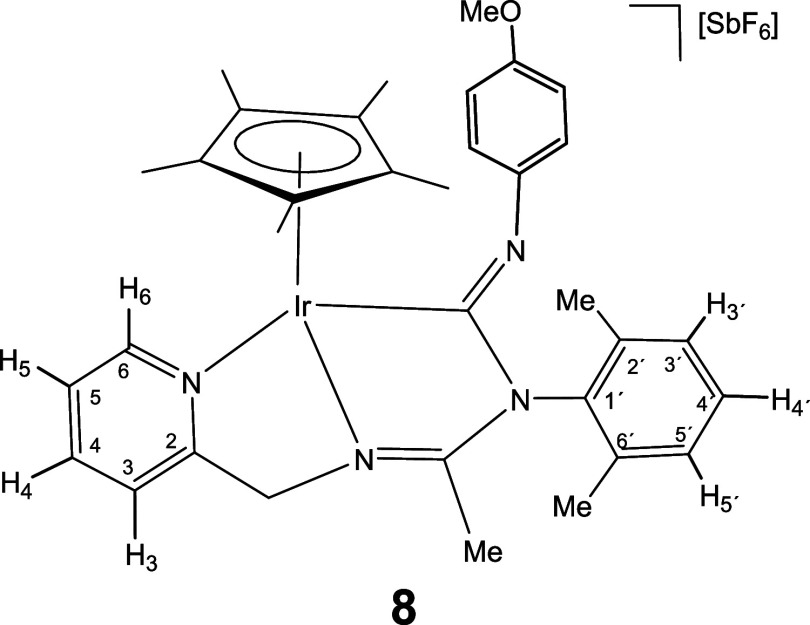


##### ^1^H NMR (300.13 MHz, CD_2_Cl_2_, RT, ppm)

δ = 8.67 (d, *J* = 6.5 Hz, 1H, H_6_); 7.98 (pt, 1H, H_4_); 7.69 (d, *J* = 5.4 Hz, 1H, H_3_); 7.54 (pt, 1H, H_5_); 7.21 (d, *J* = 4.7 Hz, 2H, H_3′_, H_5′_); 7.04 (t, 1H, H_4′_); 6.93 (d, *J* = 8.7 Hz, 2H, H_Ar_); 6.63 (d, 2H, H_Ar_); 5.38, 5.08 (AB system, *J*(AB) = 16.4 Hz, 2H, CH_2_); 3.81 (s, 3H, OMe); 2.20, 1.59 (2 × s, 6H, C_6_H_3_ *Me*_2_); 2.08 (s, 3H, Me); 1.41 (s, 15H, C_5_Me_5_).

##### ^13^C­{^1^H} NMR (75.48 MHz, CD_2_Cl_2_, RT, ppm)

δ = 172.69 (C=N); 170.30 (Ir–C=N); 165.01 (C_2_); 156.36 (C_Ar_); 152.62 (C_6_); 146.13 (C_Ar_); 140.27 (C_4_); 138.66, 136.97, 135.34 (C_1′_, C_2′_, C_6′_); 129.17, 129.08 (C_3′_, C_4′_, C_5′_); 126.64 (C_5_); 123.56 (C_Ar_); 122.23 (C_3_); 114.61 (C_Ar_); 92.41 (*C*_5_Me_5_); 61.59 (CH_2_); 56.18 (OMe); 18.98, 18.36 (C_6_H_3_ *Me*_2_); 15.31 (Me); 9.53 (C_5_ *Me*_5_).

#### Compound **9**

Yield: 76.5 mg (83%). Anal. Calcd for C_35_H_42_F_6_N_4_O_2_RhSSb: C, 45.62; H, 4.59; N, 6.08; S, 3.48. Found: C, 45.22; H, 4.49; N, 6.25; S, 3.47. HRMS (μ-TOF): C_35_H_42_N_4_O_2_RhS [M-SbF_6_]^+^: calcd 685.2078, found 685.2094. IR (cm^–1^): ν­(C=N) 1595, 1582 (br); ν­(SbF_6_) 654 (s).
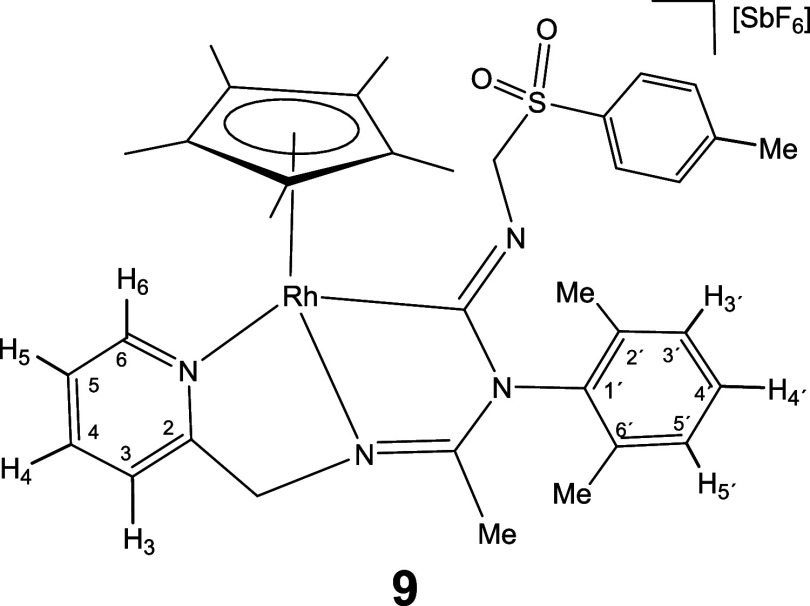


##### ^1^H NMR (300.13 MHz, CD_2_Cl_2_, RT, ppm)

δ = 8.49 (d, *J* = 5.2 Hz, 1H, H_6_); 7.93 (pt, 1H, H_4_); 7.61 (d, *J* = 7.7 Hz, 1H, H_3_); 7.54–7.06 (m, 8H, H_5_, H_3′_, H_4′_, H_5′_, H_Ar_); 5.32, 4.87 (AB system, *J*(AB) = 12.0 Hz, 2H, CH_2_S); 5.27, 5.21 (AB system, *J*(AB) = 16.2 Hz, 2H, CH_2_NRh); 2.38 (s, 3H, C_6_H_4_ *Me*); 2.07, 1.69 (2 × s, 6H, C_6_H_3_ *Me*_2_); 2.06 (s, 3H, Me); 1.70 (s, 15H, C_5_Me_5_).

##### ^13^C­{^1^H} NMR (75.48 MHz, CD_2_Cl_2_, RT, ppm)

δ = 197.30 (d, *J* = 44.7 Hz, Rh–C=N); 174.05 (C=N); 163.30 (C_2_); 154.40 (C_6_); 145.43 (C_Ar_); 140.31 (C_4_); 138.18, 137.14, 136.33, 135.39 (C_1′_, C_2′_, C_Ar_, C_6′_); 129.99 (C_Ar_); 129.62, 129.25, 129.10 (C_3′_, C_4′_, C_5′_); 128.84 (C_Ar_); 125.97 (C_5_); 122.43 (C_3_); 92.45 (d, *J* = 5.9 Hz, *C*_5_Me_5_); 82.16 (CH_2_S); 60.91 (CH_2_NRh); 21.87 (C_6_H_4_ *Me*); 18.93, 18.34 (C_6_H_3_ *Me*_2_); 16.12 (Me); 10.14 (C_5_ *Me*_5_).

#### Compound **10**

Yield: 87.9 mg (87%). Anal. Calcd for C_35_H_42_F_6_IrN_4_O_2_SSb: C, 41.59; H, 4.19; N, 5.54; S, 3.17. Found: C, 41.36; H, 4.12; N, 5.61; S, 3.29. HRMS (μ-TOF): C_35_H_42_IrN_4_O_2_S [M-SbF_6_]^+^: calcd 775.2652, found 775.2657. IR (cm^–1^): ν­(C=N) 1588, 1559 (br); ν­(SbF_6_) 654 (s).
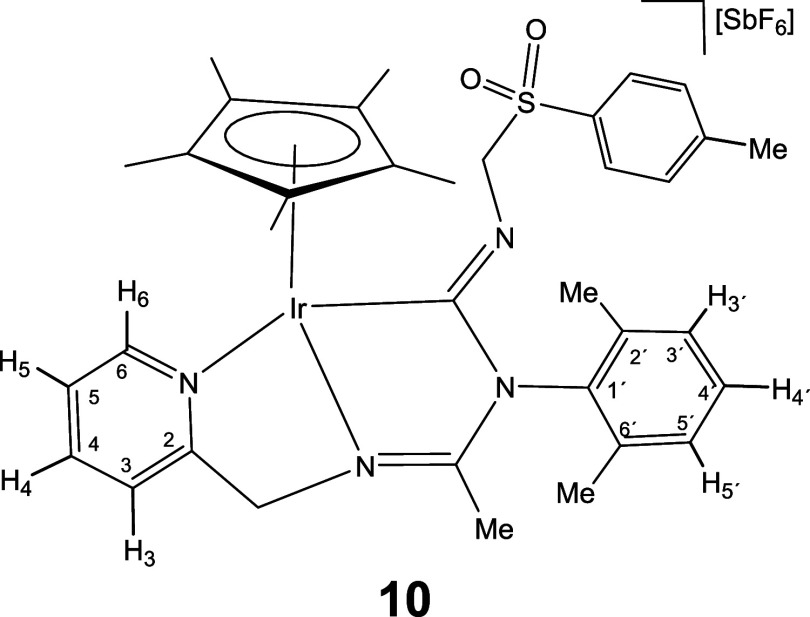


##### ^1^H NMR (300.13 MHz, CD_2_Cl_2_, RT, ppm)

δ = 8.58 (d, *J* = 6.6 Hz, 1H, H_6_); 7.92 (pt, 1H, H_4_); 7.84 (d, *J* = 7.8 Hz, 1H, H_3_); 7.48 (d, *J* = 8.2 Hz, 2H, H_Ar_); 7.40 (pt, 1H, H_5_); 7.30–7.05 (m, 5H, H_3′_, H_4′_, H_5′_, H_Ar_); 5.41, 5.06 (AB system, *J*(AB) = 16.2 Hz, 2H, CH_2_NIr); 5.28, 4.98 (AB system, *J*(AB) = 11.9 Hz, 2H, CH_2_S); 2.37 (s, 3H, C_6_H_4_ *Me*); 2.13 (s, 3H, Me); 2.03, 1.70 (2 × s, 6H, C_6_H_3_ *Me*_2_); 1.74 (s, 15H, C_5_Me_5_).

##### ^13^C­{^1^H} NMR (75.48 MHz, CD_2_Cl_2_, RT, ppm)

δ = 183.06 (Ir–C=N); 175.24 (C=N); 164.52 (C_2_); 154.74 (C_6_); 145.32 (C_Ar_); 140.41 (C_4_); 137.96, 137.03, 136.44, 135.19 (C_1′_, C_2′_, C_Ar_, C_6′_); 129.93 (C_Ar_); 129.57, 129.20, 129.07 (C_3′_, C_4′_, C_5′_); 128.82 (C_Ar_); 126.25 (C_5_); 122.13 (C_3_); 92.45 (*C*_5_Me_5_); 83.64 (CH_2_S); 62.28 (CH_2_NIr); 21.86 (C_6_H_4_ *Me*); 18.74, 18.25 (C_6_H_3_ *Me*_2_); 15.43 (Me); 9.88 (C_5_ *Me*_5_).

### Reaction of Complex 6 with Cyclohexyl Isocyanide

Under argon, at room temperature, to a solution in CH_2_Cl_2_ (6 mL) of [Cp*Ir­(κ^3^ *C*,*N′*,*N*″-**L­(CNCy)**)]­[SbF_6_] (**6**) (0.06 mmol), 15 μL (0.12 mmol) of cyclohexyl isocyanide were added. The mixture was stirred for 24 h under reflux and the resulting solution was vacuum-concentrated until *ca*. 0.5 mL. Slow addition of diethyl ether afforded an orange solid which was filtered off, washed with diethyl ether (3 × 3 mL) and vacuum-dried.

#### Compound **11**

Yield: 54.2 mg (86%). Anal. Calcd for C_40_H_55_F_6_IrN_5_Sb: C, 46.47; H, 5.36; N, 6.77. Found: C, 46.07; H, 5.03; N, 6.86. HRMS (μ-TOF): C_40_H_55_IrN_5_ [M-SbF_6_]^+^: calcd 798.4081, found 798.4088. IR (cm^–1^): ν­(C≡N) 2180 (s); ν­(C=N) 1600, 1562 (br); ν­(SbF_6_) 654 (s).
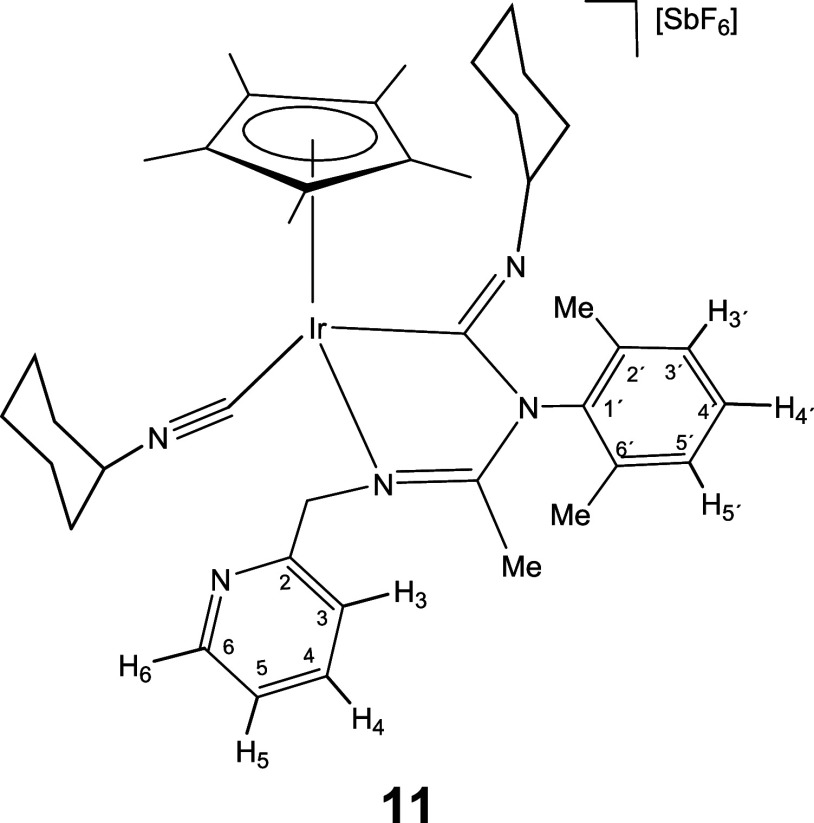


##### ^1^H NMR (300.13 MHz, CD_2_Cl_2_, RT, ppm)

δ = 8.50 (d, *J* = 5.0 Hz, 1H, H_6_); 7.77 (t, *J* = 7.7 Hz, 1H, H_4_); 7.32–7.04 (m, 5H, H_3_, H_5_, H_3′_, H_4′_, H_5′_); 5.47, 4.71 (AB system, *J*(AB) = 16.1 Hz, 2H, CH_2_); 3.51–3.14 (m, 1H, CH of Cy); 2.87–2.66 (m, 1H, CH of Cy); 2.09 (brs, 6H, C_6_H_3_ *Me*_2_); 1.99 (s, 3H, Me); 1.93 (s, 15H, C_5_Me_5_); 1.82–1.06 (m, 20H, CH_2_ of Cy).

##### ^13^C­{^1^H} NMR (75.48 MHz, CD_2_Cl_2_, RT, ppm)

δ = 170.20 (C=N); 156.74 (C_2_); 154.99 (Ir–C=N); 149.90 (C_6_); 137.64 (C_4_); 140.26, 137.33, 135.79 (C_1′_, C_2′_, C_6′_); 128.93, 128.70, 128.60 (C_3′_, C_4′_, C_5′_); 123.46 (C_5_); 122.79 (C_3_); 114.77 (Ir–C≡N); 97.61 (*C*_5_Me_5_); 65.29 (CH of Cy); 60.93 (CH_2_); 55.57 (CH of Cy); 36.36, 35.41, 33.85, 33.17, 26.26, 25.41, 25.18, 25.04, 24.05, 24.01 (10 × CH_2_ of Cy); 18.70, 18.01 (C_6_H_3_ *Me*_2_); 15.42 (Me); 10.37 (C_5_ *Me*_5_).

### Preparation and Characterization of Complexes **12–15**

Under argon, to a solution of [Cp*M­(κ^3^ *N*,*N′*,*N″*-**L**)]­[SbF_6_] (M = Rh, **1**; Ir, **2**) (0.10 mmol) in CH_2_Cl_2_ (6 mL), 0.30 mmol of the corresponding isocyanide were added. The mixture was stirred for 12 h (**12**) and 2 h (**14**) at RT or 48 h (**13**) and 8 h (**15**), under reflux. The resulting solution was vacuum-concentrated until *ca*. 0.5 mL. Addition of diethyl ether afforded a yellow (M = Rh) or pale yellow (M = Ir) solid which was filtered off, washed with diethyl ether (3 × 3 mL) and vacuum-dried.

#### Compound **12**

Yield: 78.4 mg (79%). Anal. Calcd for C_42_H_47_F_6_N_5_O_2_RhSb: C, 50.83; H, 4.77; N, 7.06. Found: C, 50.31; H, 4.57; N, 7.08. HRMS (μ-TOF): C_42_H_47_N_5_O_2_Rh [M-SbF_6_]^+^: calcd 756.2761, found 756.2779. IR (cm^–1^): ν­(C≡N) 2168 (s); ν­(C=N) 1624, 1604 (br); ν­(SbF_6_) 652 (s).
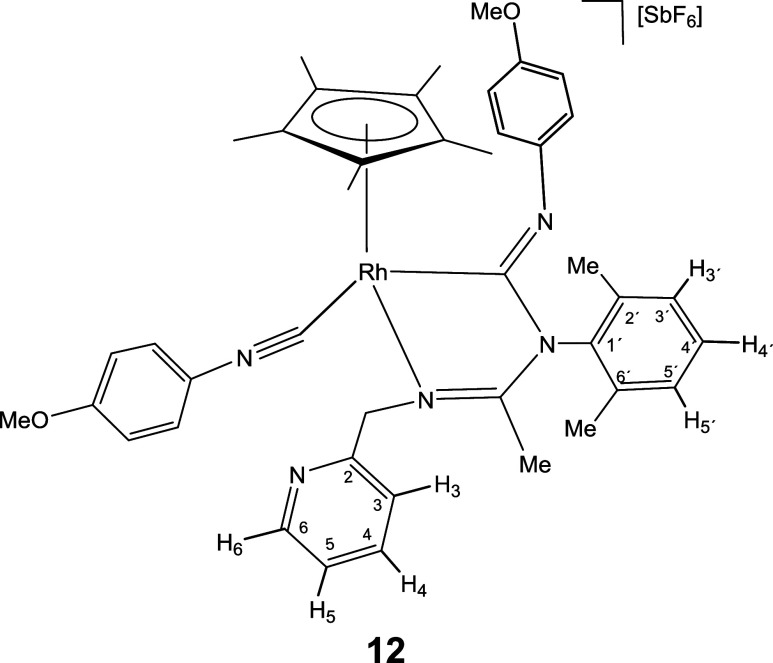


##### ^1^H NMR (300.13 MHz, CD_2_Cl_2_, RT, ppm)

δ = 8.30 (d, *J* = 4.8 Hz, 1H, H_6_); 7.63 (pt, 1H, H_4_); 7.30 (d, *J* = 7.9 Hz, 1H, H_3_); 7.27–6.87 (m, 8H, H_5_, H_3′_, H_4′_, H_5′_, H_Ar_); 6.74 (d, *J* = 9.0 Hz, 2H, H_Ar_); 6.66 (d, *J* = 9.0 Hz, 2H, H_Ar_); 5.35, 4.78 (AB system, *J*(AB) = 15.9 Hz, 2H, CH_2_); 3.87 (s, 3H, OMe); 3.70 (s, 3H, OMe); 2.31, 2.17 (2 × s, 6H, C_6_H_3_ *Me*_2_); 2.03 (s, 3H, Me); 1.65 (s, 15H, C_5_Me_5_).

##### ^13^C­{^1^H} NMR (75.48 MHz, CD_2_Cl_2_, RT, ppm)

δ = 175.19 (d, *J* = 36.8 Hz, Rh–C=N); 169.52 (C=N); 161.52 (C_Ar_); 156.31 (C_2_); 156.23 (C_Ar_); 149.97 (C_6_); 145.17 (d, *J* = 74.9 Hz, Rh–C≡N); 143.18 (C_Ar_); 140.44 (C_1′_); 137.63 (C_4_); 136.57, 136.03 (C_2′_, C_6′_); 129.28, 129.20, 129.10 (C_3′_, C_4′_, C_5′_); 128.04 (C_5_); 123.37 (C_Ar_); 122.89 (C_3_); 122.44, 115.56, 114.82 (3 × C_Ar_); 103.87 (d, *J* = 5.0 Hz, *C*_5_Me_5_); 58.99 (CH_2_); 56.42, 56.07 (2 × OMe); 18.67, 18.08 (C_6_H_3_ *Me*_2_); 16.48 (Me), 10.28 (C_5_ *Me*_5_).

#### Compound **13**

Yield: 93.0 mg (86%). Anal. Calcd for C_42_H_47_F_6_IrN_5_O_2_Sb·CH_2_Cl_2_: C, 44.26; H, 4.23; N, 6.00. Found: C, 44.15; H, 4.50; N, 6.15. HRMS (μ-TOF): C_42_H_47_IrN_5_O_2_Ir [M-SbF_6_]^+^: calcd 846.3353, found 846.3341. IR (cm^–1^): ν­(C≡N) 2158 (s); ν­(C=N) 1614, 1603 (br); ν­(SbF_6_) 652 (s).
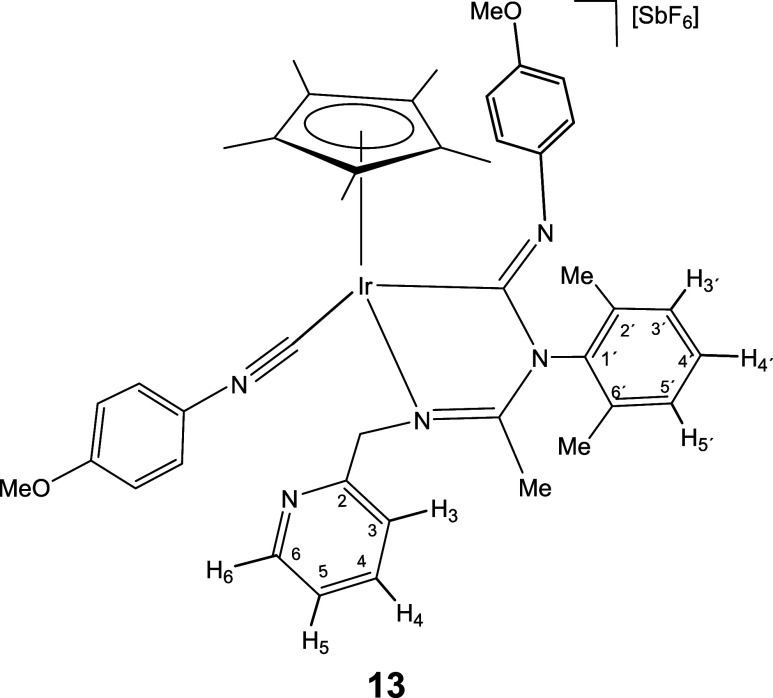


##### ^1^H NMR (300.13 MHz, CD_2_Cl_2_, RT, ppm)

δ = 8.29 (d, *J* = 4.7 Hz, 1H, H_6_); 7.63 (pt, 1H, H_4_); 7.30 (d, *J* = 7.3 Hz, 1H, H_3_); 7.28–6.86 (m, 8H, H_5_, H_3′_, H_4′_, H_5′_, H_Ar_); 6.72 (d, *J* = 9.1 Hz, 2H, H_Ar_); 6.64 (d, 2H, H_Ar_); 5.59, 4.80 (AB system, *J*(AB) = 16.0 Hz, 2H, CH_2_); 3.86 (s, 3H, OMe); 3.70 (s, 3H, OMe); 2.25, 2.20 (2 × s, 6H, C_6_H_3_ *Me*_2_); 2.14 (s, 3H, Me); 1.70 (s, 15H, C_5_Me_5_).

##### ^13^C­{^1^H} NMR (75.48 MHz, CD_2_Cl_2_, RT, ppm)

δ = 171.00 (C=N); 161.12 (C_Ar_); 157.77 (Ir–C=N); 156.02 (C_Ar_); 155.81 (C_2_); 149.99 (C_6_); 144.31, 139.94 (2 × C_Ar_); 137.69 (C_1′_); 136.51 (C_4_); 135.96, 129.25 (C_2′_, C_6′_); 129.21, 129.19, 129.09 (C_3′_, C_4′_, C_5′_); 128.01 (C_5_); 124.55 (Ir–C≡N); 123.44 (C_Ar_); 122.86 (C_3_); 122.51, 115.43, 114.78 (3 × C_Ar_); 99.03 (*C*_5_Me_5_); 60.81 (CH_2_); 56.37, 56.04 (2 × OMe); 18.55, 18.08 (C_6_H_3_ *Me*_2_); 15.83 (Me); 9.80 (C_5_ *Me*_5_).

#### Compound **14**

Yield: 97.2 mg (87%). Anal. Calcd for C_44_H_51_F_6_N_5_O_4_RhS_2_Sb: C, 47.33; H, 4.60; N, 6.27; S, 5.74. Found: C, 46.99; H, 4.49; N, 6.25; S, 5.94. HRMS (μ-TOF): C_44_H_51_N_5_O_4_RhS_2_ [M-SbF_6_]^+^: calcd 880.2432, found 880.2440. IR (cm^–1^): ν­(C≡N) 2182 (m); ν­(C=N) 1614, 1595 (br); ν­(SbF_6_) 657 (s).
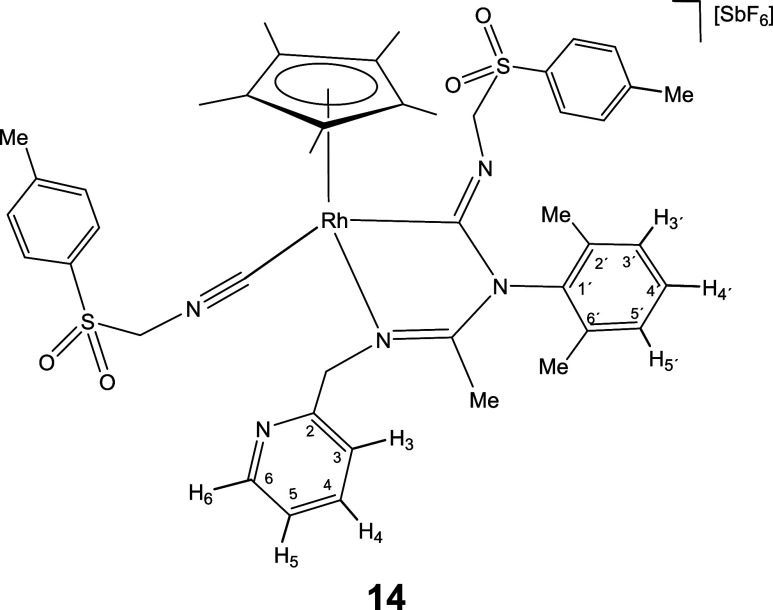


##### ^1^H NMR (300.13 MHz, CD_2_Cl_2_, RT, ppm)

δ = 8.48 (d, *J* = 4.1 Hz, 1H, H_6_); 7.79 (d, *J* = 8.2 Hz, 2H, H_Ar_); 7.72 (t, *J* = 7.8 Hz, 1H, H_4_); 7.47 (d, *J* = 8.1 Hz, 2H, H_Ar_); 7.38 (d, *J* = 8.1 Hz, 2H, H_Ar_); 7.34–7.06 (m, 7H, H_3_, H_5_, H_3′_, H_4′_, H_5′_, H_Ar_); 5.29, 4.72 (AB system, *J*(AB) = 15.6 Hz, 2H, CH_2_NRh); 5.12, 4.33 (AB system, *J*(AB) = 14.7 Hz, 2H, SCH_2_N≡C); 5.02, 4.19 (AB system, *J*(AB) = 12.2 Hz, 2H, SCH_2_N=C); 2.49 (s, 3H, C_6_H_4_ *Me*); 2.38 (s, 3H, C_6_H_4_ *Me*); 2.21, 2.07 (2 × s, 6H, C_6_H_3_ *Me*_2_); 2.05 (s, 3H, Me); 1.88 (s, 15H, C_5_Me_5_).

##### ^13^C­{^1^H} NMR (75.48 MHz, CD_2_Cl_2_, RT, ppm)

δ = 188.46 (d, *J* = 35.6 Hz, Rh–C=N); 170.40 (C=N); 156.49 (C_2_); 149.98 (C_6_); 148.05 (C_Ar_); 145.58 (d, *J* = 71.7 Hz, Rh–C≡N); 145.39 (C_Ar_); 139.70 (C_1′_); 137.98 (C_4_); 137.16, 135.86 (C_2′_, C_6′_); 135.96, 133.11, 131.30, 129.90 (4 × C_Ar_); 129.53, 129.10 (C_3′_, C_4′_, C_5′_); 129.21, 129.02 (2 × C_Ar_); 123.80 (C_5_); 123.32 (C_3_); 104.41 (d, *J* = 4.8 Hz, *C*_5_Me_5_); 80.64 (S*C*H_2_N=C); 63.81 (S*C*H_2_N≡C); 59.12 (CH_2_NRh); 22.13 (C_6_H_4_ *Me*); 21.88 (C_6_H_4_ *Me*); 18.71, 18.10 (C_6_H_3_ *Me*_2_); 16.47 (Me); 10.54 (C_5_ *Me*_5_).

#### Compound **15**

Yield: 104.9 mg (87%). Anal. Calcd for C_44_H_51_F_6_IrN_5_O_4_S_2_Sb·H_2_O: C, 43.18; H, 4.36; N, 5.72; S, 5.24. Found: C, 42.73; H, 4.32; N, 5.59; S, 5.34. HRMS (μ-TOF): C_44_H_51_IrN_5_O_4_S_2_ [M-SbF_6_]^+^: calcd 970.3006, found 970.3010. IR (cm^–1^): ν­(C≡N) 2172 (m); ν­(C=N) 1602, 1595 (br); ν­(SbF_6_) 657 (s).
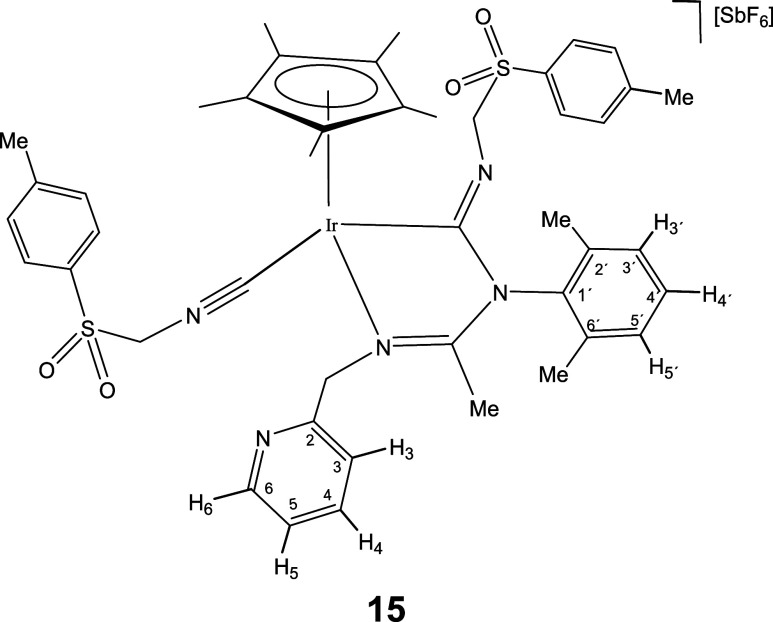


##### ^1^H NMR (300.13 MHz, CD_2_Cl_2_, RT, ppm)

δ = 8.46 (d, *J* = 4.3 Hz, 1H, H_6_); 7.78 (d, *J* = 8.3 Hz, 2H, H_Ar_); 7.71 (t, *J* = 7.6 Hz, 1H, H_4_); 7.46 (d, *J* = 8.1 Hz, 2H, H_Ar_); 7.39 (d, *J* = 8.3 Hz, 2H, H_Ar_); 7.37–7.11 (m, 7H, H_3_, H_5_, H_3′_, H_4′_, H_5′_, H_Ar_); 5.52, 4.73 (AB system, *J*(AB) = 15.5 Hz, 2H, CH_2_NIr); 5.17, 4.34 (AB system, *J*(AB) = 14.4 Hz, 2H, SCH_2_N≡C); 4.98, 4.30 (AB system, *J*(AB) = 12.0 Hz, 2H, SCH_2_N=C); 2.48 (s, 3H, C_6_H_4_ *Me*); 2.38 (s, 3H, C_6_H_4_ *Me*); 2.24, 2.03 (2 × s, 6H, C_6_H_3_ *Me*_2_); 2.07 (s, 3H, Me); 1.95 (s, 15H, C_5_Me_5_).

##### ^13^C­{^1^H} NMR (75.48 MHz, CD_2_Cl_2_, RT, ppm)

δ = 172.20 (C=N); 171.05 (Ir–C=N); 156.05 (C_2_); 149.92 (C_6_); 147.89, 145.30 (2 × C_Ar_); 139.16 (C_1′_); 138.18 (C_4_); 137.19, 136.16 (C_2′_, C_6′_); 135.77, 131.23, 131.30, 129.90 (4 × C_Ar_); 129.54, 129.48, 129.08 (C_3′_, C_4′_, C_5′_); 129.20, 129.03 (2 × C_Ar_); 126.06 (Ir–C≡N); 123.80 (C_5_); 123.35 (C_3_); 99.66 (*C*_5_Me_5_); 82.18 (S*C*H_2_N=C); 63.91 (S*C*H_2_N≡C); 60.86 (CH_2_NIr); 22.11 (C_6_H_4_ *Me*); 21.87 (C_6_H_4_ *Me*); 18.58, 18.10 (C_6_H_3_ *Me*_2_); 15.86 (Me); 10.15 (C_5_ *Me*_5_).

### Preparation and Characterization of Complex **16**

Under argon, at room temperature, to a suspension of [Cp*Ir­(κ^3^ *N*,*N′*,*N*″-**L**)]­[SbF_6_] (**2**) (81.5 mg, 0.10 mmol) in CH_2_Cl_2_ (6 mL), 10.6 μL (0.10 mmol) of CN^*t*^Bu were added. An instantaneous color change, from pale yellow to intense yellow, was observed and after 15 min of stirring the resulting solution was vacuum-concentrated until *ca*. 0.5 mL. Slow addition of diethyl ether afforded a yellow solid which was filtered off, washed with diethyl ether (3 × 3 mL) and vacuum-dried.

#### Compound **16**

Yield: 74.2 mg (91%). Anal. Calcd for C_31_H_42_F_6_N_4_IrSb: C, 41.43; H, 4.71; N, 6.23. Found: C, 41.26; H, 4.79; N, 6.32. HRMS (μ-TOF): C_31_H_42_N_4_IrSbF_6_ [M-SbF_6_]^+^: calcd 664.3111, found 664.3094. IR (cm^–1^): ν­(C≡N) 2189 (s); ν­(C=N) 1574, 1556 (br); ν­(SbF_6_) 654 (s).
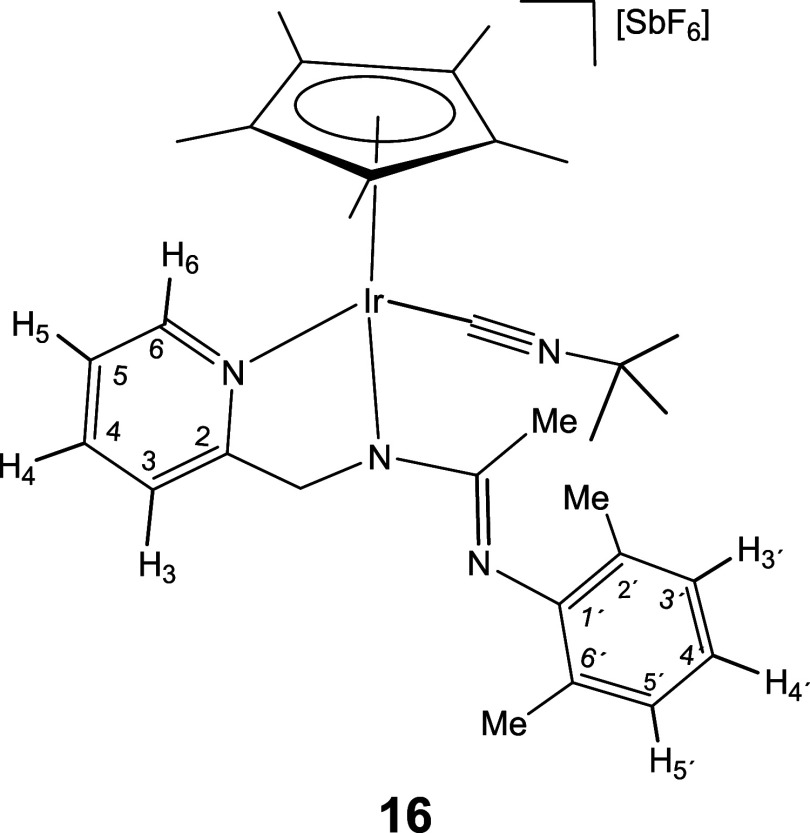


##### ^1^H NMR (300.13 MHz, CD_2_Cl_2_, RT, ppm)

δ = 8.47 (d, *J* = 6.4 Hz, 1H, H_6_); 7.94 (pt, 1H, H_4_); 7.64 (d, *J* = 7.7 Hz, 1H, H_3_); 7.42 (pt, 1H, H_5_); 6.94 (dd, *J* = 11.6, 7.4 Hz, 2H, H_3′_, H_5′_); 6.70 (pt, 1H, H_4′_); 6.11, 4.75 (2 × d, *J* = 19.2 Hz, 2H, CH_2_); 2.07, 1.90 (2 × s, 6H, C_6_H_3_ *Me*_2_); 1.73 (s, 15H, C_5_Me_5_); 1.56 (s, 3H, Me); 1.47 (s, 9H, CMe_3_).

##### ^13^C­{^1^H} NMR (75.48 MHz, CD_2_Cl_2_, RT, ppm)

δ = 167.10 (C_2_); 159.29 (C=N); 152.27 (C_6_); 151.96 (C_1′_); 140.02 (C_4_); 129.53, 129.17 (C_2′_, C_6′_); 127.64 (C_3′_, C_5′_); 125.74 (C_5_); 121.98 (C_3_); 120.46 (C_4′_); 116.69 (Ir–C≡N); 94.92 (*C*_5_Me_5_); 61.30 (CH_2_); 59.45 (*C*Me_3_); 30.18 (C*Me*_3_); 21.14 (Me); 19.03, 18.59 (C_6_H_3_ *Me*_2_); 9.21 (C_5_ *Me*_5_).

### Preparation and Characterization of Complex **17**

Under argon, at room temperature, to a suspension of [Cp*Ir­(CN^*t*^Bu)­(κ^2^ *N,N′*-**L**)]­[SbF_6_] (**16**) (89.8 mg, 0.10 mmol) in CH_2_Cl_2_ (6 mL), HSbF_6_·6H_2_O (8.8 μL, 0.10 mmol) was added. After 30 min of stirring, the resulting solution was vacuum-concentrated until *ca*. 1 mL. Addition of diethyl ether afforded a yellow solid which was filtered off, washed with the precipitant (3 × 3 mL) and vacuum-dried. The isolated solid consists of a mixture of two isomers in a 72:28 molar ratio.

#### Compound **17**

Yield: 88.6 mg (78%). Anal. Calcd for C_31_H_43_F_12_N_4_IrSb_2_ ·CH_2_Cl_2:_ C, 31.49; H, 3.72; N, 4.59. Found: C, 31.03; H, 3.70; N, 4.60. HRMS (μ-TOF): C_31_H_42_N_4_Ir [M-(SbF_6_)_2_-H]^+^: calcd 663.3039, found 663.3040. IR (cm^–1^): ν­(NH) 3355 (w); ν­(C≡N) 2208 (s); ν­(C=N) 1614 (br); ν­(SbF_6_) 652 (s).
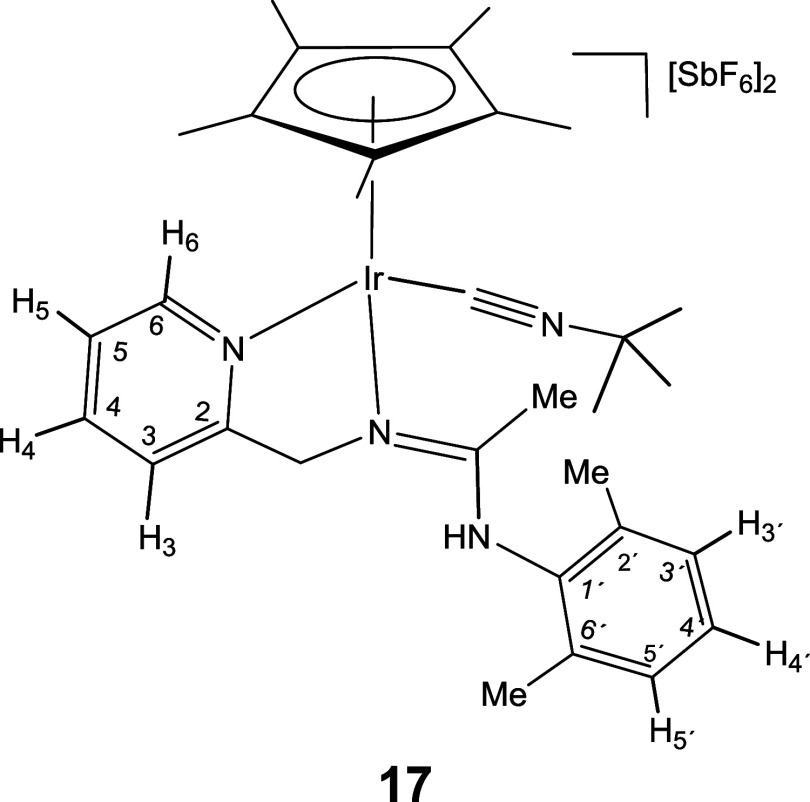


##### Major Isomer

^1^H NMR (300.13 MHz, acetone-*d*_6_, RT, ppm): δ = 8.94 (d, *J* = 6.8 Hz, 1H, H_6_); 8.27 (pt, 1H, H_4_); 7.88 (d, *J* = 7.8 Hz, 1H, H_3_); 7.72 (pt, 1H, H_5_); 7.34–6.94 (m, 3H, H_3′_, H_4′_, H_5′_); 5.90, 5.31 (2 × d, *J* = 18.0 Hz, 2H, CH_2_); 2.36, 2.17 (2 × s, 6H, C_6_H_3_ *Me*_2_); 2.18 (s, 3H, Me); 1.96 (s, 15H, C_5_Me_5_); 1.60 (s, 9H, CMe_3_).

##### ^13^C­{^1^H} NMR (75.48 MHz, Acetone-*d*_6_, RT, ppm)

δ = 166.12 (C_2_); 164.28 (C=N); 153.49 (C_6_); 142.21 (C_4_); 137.33, 137.10, 136.45 (C_1′_, C_2′_, C_6′_); 129.83, 129.77, 129.73 (C_3′_, C_4′_, C_5′_); 127.84 (C_5_); 123.73 (C_3_); 98.09 (Ir–C≡N); 97.34 (*C*_5_Me_5_); 61.95 (CH_2_); 61.02 (*C*Me_3_); 30.13 (C*Me*_3_); 24.61 (Me); 18.45, 18.14 (C_6_H_3_ *Me*_2_); 9.18 (C_5_ *Me*_5_).

### Preparation and Characterization of Complexes **18–21**

Under argon, at room temperature, to a solution of [Cp*M­(κ^3^ *N*,*N′*,*N″-* **L**)]­[SbF_6_] (M = Rh, **1**; Ir, **2**) (0.10 mmol) in THF (5 mL), HC≡CR (R = Ph, COOEt) (0,10 mmol) was added. The solution was stirred for 1 h and then concentrated until *ca*. 0.5 mL. Addition of diethyl ether afforded a yellow solid which was filtered off, washed with the precipitant (3 × 3 mL) and vacuum-dried. The isolated solid **19** consists of a mixture of two isomers in a 87/13 molar ratio.

#### Compound **18**

Yield: 70.4 mg (85%). Anal. Calcd for C_34_H_39_F_6_N_3_RhSb: C, 49.30; H, 4.75; N, 5.07. Found: C, 48.97; H, 4.73; N, 5.02. HRMS (μ-TOF): C_34_H_39_N_3_Rh [M-SbF_6_]^+^: calcd 592.2194, found 592.2198. IR (cm^–1^): ν­(NH) 3284 (w); ν­(C≡C) 2104 (s); ν­(C=N) 1625, 1615 (s); ν­(SbF_6_) 654 (s).
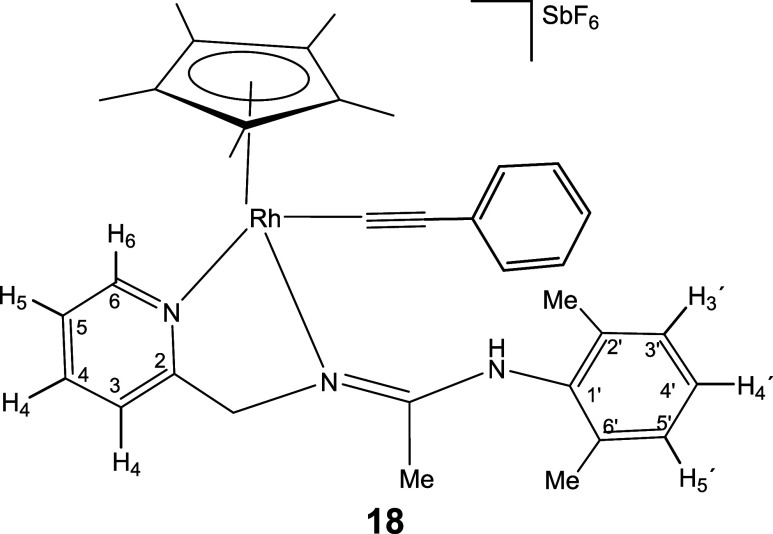


##### ^1^H NMR (300.13 MHz, CD_2_Cl_2_, RT, ppm)

δ = 8.45 (d, *J* = 6.5 Hz, 1H, H_6_); 7.98 (pt, 1H, H_4_); 7.72 (s, 1H, NH); 7.63 (d, *J* = 7.7 Hz, 1H, H_3_); 7.50 (pt, 1H, H_5_); 7.29–6.97 (m, 8H,, H_3′_, H_4′_, H_5′_, H_Ar_); 5.16, 4.93 (AB system, *J*(AB) = 17.3 Hz, 2H, CH_2_); 2.35, 2.11 (2 × s, 6H, C_6_H_3_ *Me*_2_); 2.05 (s, 3H, Me); 1.77 (s, 15H, C_5_Me_5_).

##### ^13^C­{^1^H} NMR (75.48 MHz, CD_2_Cl_2_, RT, ppm)

δ = 167.37 (C=N); 161.29 (C_2_); 152.19 (C_6_); 139.93 (C_4_); 137.79, 136.46, 135.85 (C_1′_, C_2′_, C_6′_); 131.80, 129.37, 128.73, 128.50, 127.92, 126.37, 125.95 (6 × C_Ar_, C_3′_, C_4′_, C_5′_); 125.95 (C_5_); 122.43 (C_3_); 107.95 (d, *J* = 9.8 Hz, RhC*C*); 105.47 (d, *J* = 57.2 Hz, Rh*C*C); 98.19 (d, *J* = 6.5 Hz, *C*_5_Me_5_); 61.36 (CH_2_); 19.38, 19.22 (C_6_H_3_ *Me*_2_); 15.79 (Me); 9.89 (C_5_ *Me*_5_).

#### Compound **19**

Yield: 75.2 mg (82%). Anal. Calcd for C_34_H_39_F_6_IrN_3_Sb: C, 44.50; H, 4.28; N, 4.58. Found: C, 44.28; H, 4.34; N, 4.60. HRMS (μ-TOF): C_34_H_39_IrN_3_ [M-SbF_6_]^+^: calcd 682.2773, found 682.2770. IR (cm^–1^): ν­(NH) 3251 (w); ν­(C≡C) 2106 (s); ν­(C=N) 1622 (s); ν­(SbF_6_) 654 (s).
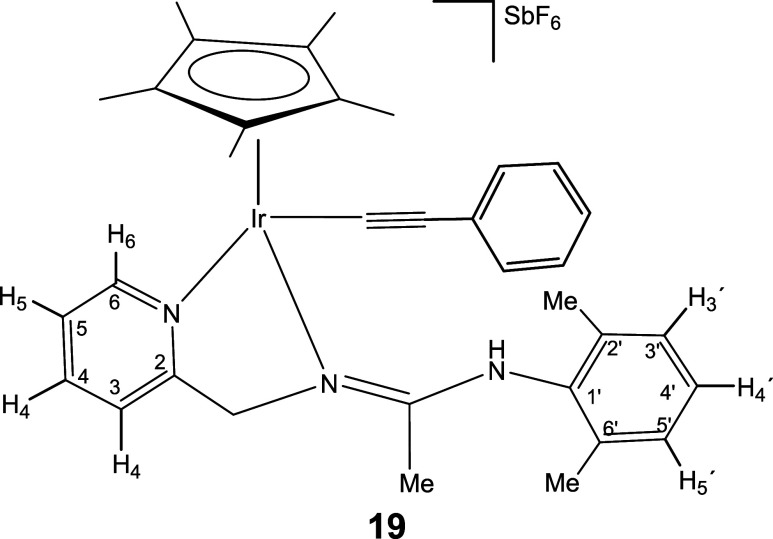


##### Major Isomer, 87%. ^1^H NMR (300.13 MHz, CD_2_Cl_2_, RT, ppm)

δ = 8.49 (d, *J* = 6.6 Hz, 1H, H_6_); 7.98 (t, *J* = 7.8 Hz, 1H, H_4_); 7.74 – 7.64 (m, 2H, NH, H_3_); 7.44 (pt, 1H, H_5_); 7.26–6.97 (m, 8H, H_3′_, H_4′_, H_5′_, H_Ar_); 5.42, 4.80 (AB system, *J*(AB) = 17.2 Hz, 2H, CH_2_); 2.35 (s, 3H, C_6_H_3_ *Me*_2_); 2.12 (brs, 6H, C_6_H_3_ *Me*_2_, Me); 1.79 (s, 15H, C_5_Me_5_).

##### ^13^C­{^1^H} NMR (75.48 MHz, CD_2_Cl_2_, RT, ppm)

δ = 166.41 (C=N); 162.29 (C_2_); 151.98 (C_6_); 139.93 (C_4_); 137.94, 136.42, 135.63 (C_1′_, C_2′_, C_6′_); 132.22, 129.43, 129.41, 128.86, 128.43, 126.26 (5 × C_Ar_, C_3′_, C_4′_, C_5′_); 126.18 (C_5_); 121.98 (C_3_); 105.26 (C_Ar_); 91.15 (*C*_5_Me_5_); 90.34 (IrC*C*); 89.28 (Ir*C*C); 63.38 (CH_2_); 19.35, 19.29 (C_6_H_3_ *Me*_2_); 15.38 (Me); 9.63 (C_5_ *Me*_5_).

##### Minor Isomer, 13%. ^1^H NMR (300.13 MHz, CD_2_Cl_2_, RT, ppm)

δ = 1.71 (s, 15H, C_5_Me_5_).

##### ^13^C­{^1^H} NMR (75.48 MHz, CD_2_Cl_2_, RT, ppm)

δ = 9.46 (C_5_ *Me*_5_).

#### Compound **20**

Yield: 70.1 mg (85%). Anal. Calcd for C_31_H_39_F_6_N_3_O_2_RhSb: C, 45.17; H, 4.77; N, 5.10. Found: C, 45.00; H, 4.54; N, 5.10. HRMS (μ-TOF): C_31_H_39_N_3_O_2_Rh [M-SbF_6_]^+^: calcd 588.2092, found 588.2075. IR (cm^–1^): ν­(NH) 3289 (w); ν­(C≡C) 2101 (s); ν­(C=O) 1674 (s); ν­(C=N) 1625, 1610 (s); ν­(SbF_6_) 654 (s).
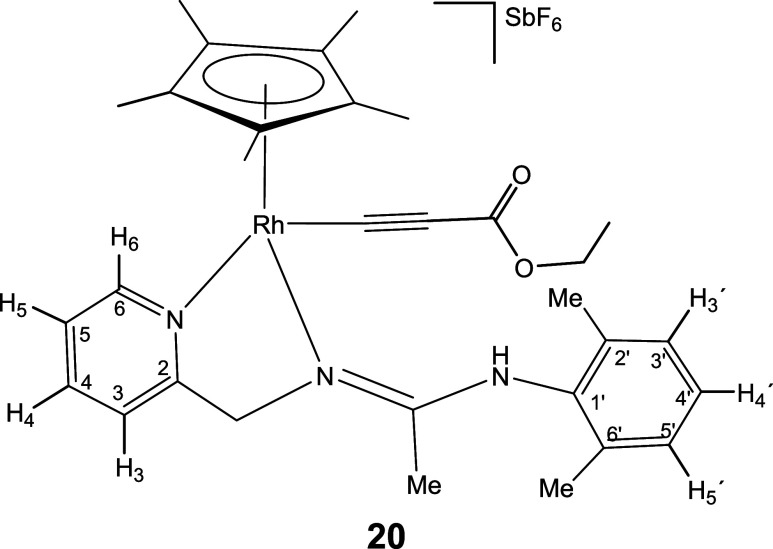


##### ^1^H NMR (300.13 MHz, CD_2_Cl_2_, RT, ppm)

δ = 8.40 (d, *J* = 6.5 Hz, 1H, H_6_); 7.98 (pt, 1H, H_4_); 7.63 (d, *J* = 7.7 Hz, 1H, H_3_); 7.52 (pt, 1H, H_5_); 7.25–7.03 (m, 4H, NH, H_3′_, H_4′_, H_5′_); 5.14, 4.90 (AB system, *J*(AB) = 17.3 Hz, 2H, CH_2_); 4.03 (q, *J* = 7.1 Hz, 2H, OC*H*_2_CH_3_); 2.34, 2.21 (2 × s, 6H, C_6_H_3_ *Me*_2_); 2.05 (s, 3H, Me); 1.74 (s, 15H, Me, C_5_Me_5_); 1.18 (t, *J* = 7.1 Hz, 3H, OCH_2_C*H*_***3***_ **)**.

##### ^13^C­{^1^H} NMR (75.48 MHz, CD_2_Cl_2_, RT, ppm)

δ = 167.65 (C=N); 161.28 (C_2_); 153.85 (C=O); 152.01 (C_6_); 140.29 (C_4_); 138.12, 136.53, 135.45 (C_1′_, C_2′_, C_6′_); 129.51, 129.45, 129.02 (C_3′_, C_4′_, C_5′_); 126.24 (C_5_); 122.76 (C_3_); 112.89 (d, *J* = 58.4 Hz, Rh*C*C); 101.14 (d, *J* = 10.2 Hz, RhC*C*); 99.28 (d, *J* = 6.5 Hz, *C*_5_Me_5_); 61.54 (CH_2_); 61.22 (O*CH*_2_CH_**3**_); 19.40, 19.28 (C_6_H_3_ *Me*_2_); 15.87 (Me); 14.50 (OCH_2_ *CH*_***3***_); 9.84 (C_5_ *Me*_5_).

#### Compound **21**

Yield: 78.6 mg (86%). Anal. Calcd for C_31_H_39_F_6_IrN_3_O_2_Sb: C, 40.76; H, 4.30; N, 4.60 Found: C, 40.71; H, 4.18; N, 4.63. HRMS (μ-TOF): C_31_H_39_IrN_3_O_2_ [M-SbF_6_]^+^: calcd 678.2666, found 678.2684. IR (cm^–1^): ν­(NH) 3284 (w); ν­(C≡N) 2101 (s); ν­(C=O) 1679 (s); ν­(C=N) 1625 (s); ν­(SbF_6_) 657 (s).
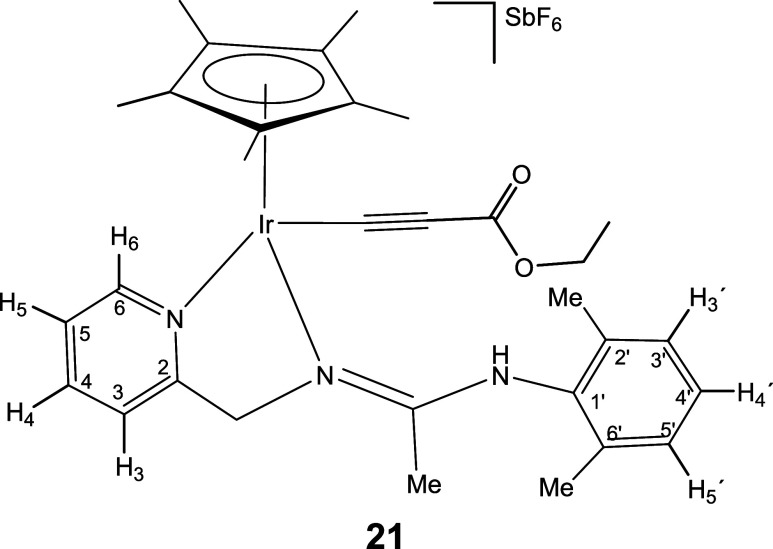


##### ^1^H NMR (300.13 MHz, CD_2_Cl_2_, RT, ppm)

δ = 8.44 (d, *J* = 6.5 Hz, 1H, H_6_); 8.00 (pt, 1H, H_4_); 7.71 (d, *J* = 7.8 Hz, 1H, H_3_); 7.47 (pt, 1H, H_5_); 7.28–6.94 (m, 4H, NH, H_3′_, H_4′_, H_5′_); 5.41, 4.81 (AB system, *J*(AB) = 17.4 Hz, 2H, CH_2_); 4.02 (q, *J* = 7.1 Hz, 2H, OC*H*_2_CH_**3**_); 2.34, 2.23 (2 × s, 6H, C_6_H_3_ *Me*_2_); 2.13 (s, 3H, Me); 1.77 (s, 15H, C_5_Me_5_); 1.18 (t, *J* = 7.1 Hz, 3H, OCH_2_ *CH*_***3***_).

##### ^13^C­{^1^H} NMR (75.48 MHz, CD_2_Cl_2_, RT, ppm)

δ = 166.81 (C=N); 162.39 (C_2_); 154.55 (C=O); 151.92 (C_6_); 140.42 (C_4_); 138.22, 136.46, 135.25 (C_1′_, C_2′_, C_6′_); 129.57, 129.55, 129.17 (C_3′_, C_4′_, C_5′_); 126.54 (C_5_); 122.34 (C_3_); 99.11 (IrC*C*); 95.96 (Ir*C*C); 92.49 (*C*_5_Me_5_); 63.55 (CH_2_); 61.18 (O*CH*_2_CH_**3**_); 19.36, 19.35 (C_6_H_3_ *Me*_2_); 15.45 (Me); 14.54 (OCH_2_ *CH*_***3***_); 9.57 (C_5_ *Me*_5_).

### Preparation and Characterization of Complex **22**

Under argon, at room temperature, to a solution of [Cp*Ir­(κ^3^ *N*,*N*′,*N*″*-* **L**)]­[SbF_6_] (**2**) (81.5 mg, 0.10 mmol) in THF (6 mL), dimethyl acetylenedicarboxylate (24.5 μL, 0.20 mmol) was added. The mixture was heated under reflux for 20 h and a yellow solid precipitated. The resulting suspension was vacuum-concentrated until *ca*. 0.5 mL and diethyl ether was added (3 mL). The solid obtained was filtered off, washed with the precipitant (3 × 3 mL) and vacuum-dried.

Yield: 79.5 mg (83%) Anal. Calcd for C_32_H_39_F_6_IrN_3_O_2_Sb: C, 40.14; H, 4.10; N, 4.39. Found: C, 39.78; H, 3.86; N, 4.41. HRMS (μ-TOF): C_32_H_39_IrN_3_O_2_ [M-SbF_6_]^+^: calcd 722.2570, found 722.2585. IR (cm^–1^): ν­(C=O) 1725 (s), 1685 (s); ν­(C=N) 1610 (br); ν­(SbF_6_) 657 (s).
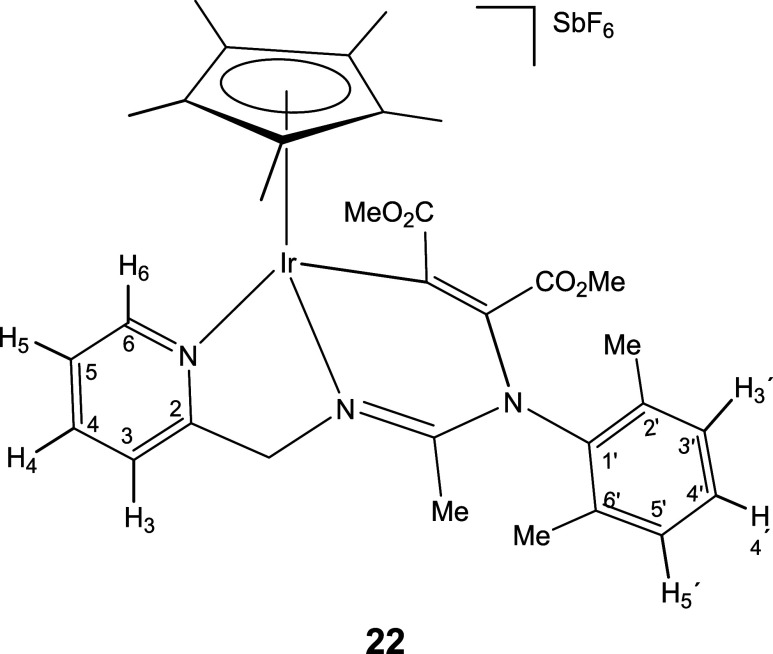


#### ^1^H NMR (300.13 MHz, CD_2_Cl_2_, RT, ppm)

δ = 8.66 (d, *J* = 6.5 Hz, 1H, H_6_); 7.94 (pt, 1H, H_4_); 7.68 (d, *J* = 7.7 Hz, 1H, H_3_); 7.43 (pt, 1H, H_5_); 7.30–7.03 (m, 3H, H_3′_, H_4′_, H_5′)_; 5.60, 4.99 (AB system, *J*(AB) = 15.7 Hz, 2H, CH_2_); 3.72, 3.19 (2 × s, 6H, 2 × OMe); 2.32, 1.90 (2 × s, 6H, C_6_H_3_ *Me*_2_); 2.06 (s, 3H, Me); 1.61 (s, 15H, C_5_Me_5_).

#### ^13^C­{^1^H} NMR (75.48 MHz, CD_2_Cl_2_, RT, ppm)

δ = 173.67, 164.20 (2 × C=O); 159.34 (C_2_); 158.85 (C=N); 153.04 (C_6_); 140.20 (C_4_); 139.03, 138.02, 137.08 (C_1′_, C_2′_, C_6′_); 134.57, 114.56 (C=C); 130.19, 129.94, 129.23 (C_3′_, C_4′_, C_5′_); 125.75 (C_5_); 121.60 (C_3_); 90.35 (*C*_5_Me_5_); 66.59 (CH_2_); 52.51, 51.99 (2 × OMe); 19.98 (Me); 19.20, 18.14 (C_6_H_3_ *Me*_2_); 9.53 (C_5_ *Me*_5_).

### Crystal Structure Determination of Complexes **3**, **8**, **11**, **16**, **17**, and **22**

Suitable crystals for the X-ray experiments were obtained for **3**, **8**, **11**, **16**, **17**, and **22** complexes from solutions of THF/diethyl ether (**3**), CH_2_Cl_2_/MeOH/diethyl ether (**8, 11, 17** and **22**), or CH_2_Cl_2_/diethyl ether (**16**). Intensity data were measured at low temperature 100(2) K on a Bruker D8 Venture diffractometer, equipped with graphite-monochromated Mo Kα radiation (λ = 0.71073 Å) using narrow frames (Δω = 0.3°). Data were integrated and corrected for Lorentz and polarization effects with SAINT program^31^ included in APEX4 package. Semiempirical absorption corrections were performed with SADABS program^32^ Structures were solved by direct methods with SHELXS,^33^ completed by reiterative difference Fourier synthesis and refined by full-matrix least-squares on *F* ^2^ with SHELXL program^34^ included in Olex2 package.^35^ Hydrogen atoms were included in the models in calculated positions and refined with a riding model. Special refinement details concerning disorder or restraints are mentioned below.

#### Crystal Data for Complex **3**: C_28_H_33_F_6_N_3_O_2_RhSb

*M*_r_ = 782.23; yellow prism, 0.110 × 0.170 × 0.180 mm^3^; monoclinic *P*2_1_/*c*; *a* = 11.4053(5), *b* = 12.8144(5), *c* = 20.8288(9) Å, β = 101.5850(10)°; *V* = 2982.2(2) Å^3^, *Z* = 4, *D*_c_ = 1.742 g/cm^3^; μ = 1.527 cm^–1^; min and max. absorption correction factors: 0.6930 and 0.7465; 2θ_max_ = 66.34°; 104,548 reflections measured, 11,358 unique; *R*_int_ = 0.0218; number of data/restraint/parameters 11,358:13:411; *R*_1_ = 0.0286 [10,677 reflections, *I* > 2σ­(*I*)], *wR*2 = 0.0710 (all data); largest difference peak 1.924 e·Å^–3^. Four fluorine atoms of SbF_6_ and the C_5_H_4_N ligand have been found to be disordered. They have been included in the model in two sets of positions. Some restraints have been used in the refinement of the C_5_H_4_N ring geometry, as major and minor component bond lengths have been considered to be similar.

#### Crystal Data for Complex **8**: C_34_H_40_F_6_IrN_4_OSb

*M*_r_ = 948.65; yellow plate, 0.040 × 0.100 × 0.100 mm^3^; monoclinic *P*2_1_/*c*; *a* = 13.9723(4) Å, *b* = 16.7346(5) Å, *c* = 14.6200(4) Å, β = 96.1300(10)°; *V* = 3398.92(17) Å^3^, *Z* = 4, *D*_c_ = 1.854 g/cm^3^; μ = 4.773 cm^–1^; min and max. absorption correction factors: 0.6166 and 0.7461; 2θ_max_ = 61.094°; 146,105 reflections measured, 10,382 unique; *R*_int_ = 0.0354; number of data/restraint/parameters 10,382:0:433; *R*_1_ = 0.0153 [9822 reflections, *I* > 2σ­(*I*)], *wR*2 = 0.0367 (all data); largest difference peak 1.158 e·Å^–3^.

#### Crystal Data for Complex **11**

C_40_H_55_F_6_IrN_5_Sb; *M*_r_ = 1033.84; colorless block, 0.050 × 0.060 × 0.120 mm^3^; orthorhombic *Pbca*; *a* = 15.8752(6), *b* = 16.7200(6), *c* = 31.2164(10) Å; *V* = 8285.9(5) Å^3^, *Z* = 8, *D*_c_ = 1.657 g/cm^3^; μ = 3.922 cm^–1^; min and max. absorption correction factors: 0.6405 and 0.7457; 2θ_max_ = 56.632°; 259,620 reflections measured, 10,305 unique; *R*_int_ = 0.0465; number of data/restraint/parameters 10,305:0:486; *R*_1_ = 0.0184 [9441 reflections, *I* > 2σ­(*I*)], *wR*2 = 0.0429 (all data); largest difference peak 0.442 e·Å^–3^. Iridium atom has been anharmonically refined.^35^

#### Crystal Data for Complex **16**

##### C_31_H_42_F_6_IrN_4_Sb·CH_2_Cl_2_

*M*_r_ = 983.56; yellow plate, 0.050 × 0.150 × 0.200 mm^3^; monoclinic *P*2_1_/*n*; *a* = 14.3485(5), *b* = 14.8188(6), *c* = 17.7801(7) Å, β = 108.9400(10)°; *V* = 3575.9(2) Å^3^, *Z* = 4, *D*_c_ = 1.827 g/cm^3^; μ = 4.682 cm^–1^; min and max. absorption correction factors: 0.4977 and 0.7457; 2θ_max_ = 56.598°; 111,130 reflections measured, 8873 unique; *R*_int_ = 0.0376; number of data/restraint/parameters 8873:0:473; *R*_1_ = 0.0183 [8631 reflections, *I* > 2σ­(*I*)], *wR*2 = 0.0441 (all data); largest difference peak 2.096 e·Å^–3^. Four fluorine atoms of SbF_6_ and a chlorine atom of CH_2_Cl_2_ have been found to be disordered. They have been included in the model in two sets of positions and refined with complementary occupancy factors.

#### Crystal Data for Complex **17**

C_31_H_43_F_12_IrN_4_Sb_2_; *M*_r_ = 1135.39; yellow prism, 0.12 × 0.15 × 0.15 mm^3^; monoclinic *P*2_1_/*n*; *a* = 11.0094(4), *b* = 37.7073(14), *c* = 18.7066(7) Å, β = 102.9810(10)°; *V* = 7567.3(2) Å^3^, *Z* = 8, *D*_c_ = 1.993 g/cm^3^; μ = 5.013 cm^–1^; min and max. absorption correction factors: 0.6320 and 0.7457; 2θ_max_ = 56.584°; 185,139 reflections measured, 18,763 unique; *R*_int_ = 0.0387; number of data/restraint/parameters 18,763:1:1031; *R*_1_ = 0.0193 [18,290 reflections, *I* > 2σ­(*I*)], *wR*2 = 0.0416 (all data); largest difference peak 0.925 e·Å^–3^. Asymmetric unit contains two chemically equivalent molecules. Hydrogens of NH fragments have been included in the model in observed positions and refined with a geometrical restraint in one N–H bond length.

#### Crystal Data for Complex **22**

C_32_H_39_F_6_IrN_3_O_4_Sb; *M*_r_ = 957.61; yellow prism, 0.065 × 0.120 × 0.155 mm^3^; monoclinic *P*2_1_/*c*; *a* = 8.1816(5), *b* = 22.3579(14), *c* = 18.3258(11) Å, β = 92.329(2)°; *V* = 3349.4(4) Å^3^, *Z* = 4, *D*_c_ = 1.899 g/cm^3^; μ = 4.850 cm^–1^; min and max. absorption correction factors: 0.6292 and 0.7457; 2θ_max_ = 56.622°; 124,273 reflections measured, 8308 unique; *R*_int_ = 0.0305; number of data/restraint/parameters 8308:0:452; *R*_1_ = 0.0139 [8291 reflections, *I* > 2σ­(*I*)], *wR*2 = 0.0329 (all data); largest difference peak 0.388 e·Å^–3^. One of the CO_2_Me fragments have been found to be disordered. Concerned atoms have been included in the model in two sets of positions and refined with complementary occupancy factors.

## Supplementary Material



## Notes

^1^H and ^13^C­{^1^H} NMR spectra for the complexes **3**-**22** and relevant NOE interactions (PDF)
