# Supplementary material for: Activation of CO, Isocyanides, and Alkynes by Frustrated Lewis Pairs Based on Cp*M/N (M = Rh, Ir) Couples
Source: Inorg Chem. 2025 May 19;64(21):10435–51. doi: 10.1021/acs.inorgchem.5c00332 (PMC12135047; doi:10.1021/acs.inorgchem.5c00332)
Supplement: Supplementary file 1 [file ic5c00332_si_001.pdf]

Supporting Information

Activation of CO, Isocyanides and Alkynes by Frustrated Lewis Pairs Based on Cp*M/N (M = Rh, Ir) Couples

Carlos Ferrer-Bru,[a] Joaquina Ferrer,[,a] Fernando J. Lahoz,*[,a] Pilar García-Orduña[a] and Daniel Carmona*[,a]*

[a] *Instituto de Síntesis Química y Catálisis Homogénea (ISQCH), CSIC - Universidad de Zaragoza, Departamento de Química Inorgánica, Pedro Cerbuna 12, 50009 Zaragoza, Spain. E-mail: jfecer@unizar.es (J. F.), dcarmona@unizar.es (D. C.)*

Table of Contents

1. ^{1}H and $^{13}C\{^{1}H\}$ NMR spectra for the complexes 3-22 **S2**

2. Relevant NOE interactions **S22**

1. ^{1}H and ^{13}C{^{1}H} NMR spectra for the complexes 3-22

Fig. S1. ^{1}H-NMR (THF-d_8, RT) spectrum of **3**

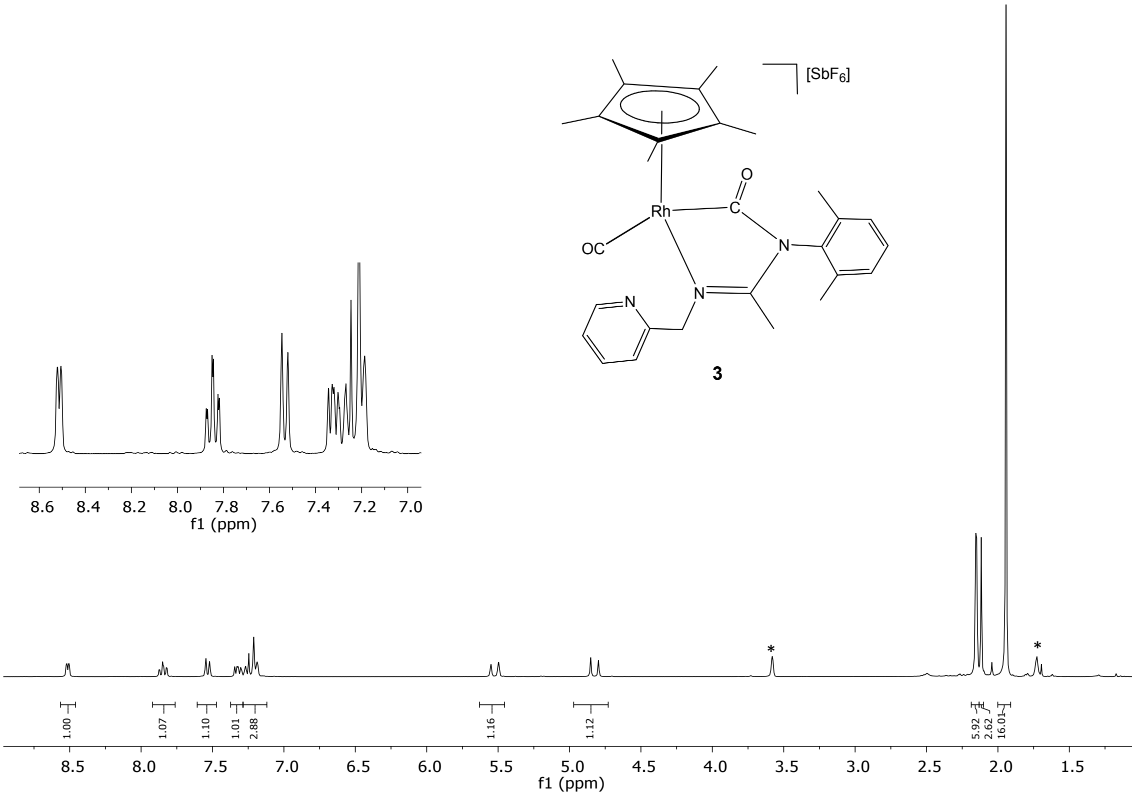

Fig. S2. ^{13}C{^{1}H}-NMR (THF-d_8, RT) spectrum of **3**

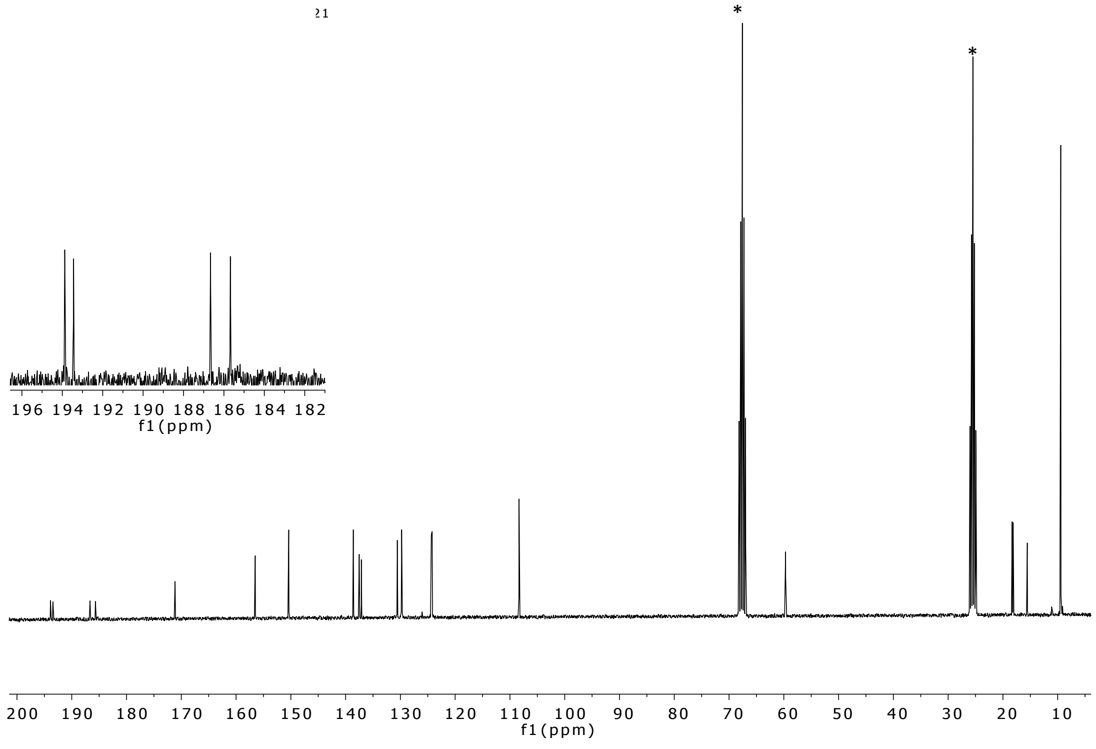

Fig. S3. ^{1}H-NMR (THF-d_8, RT) spectrum of **4**

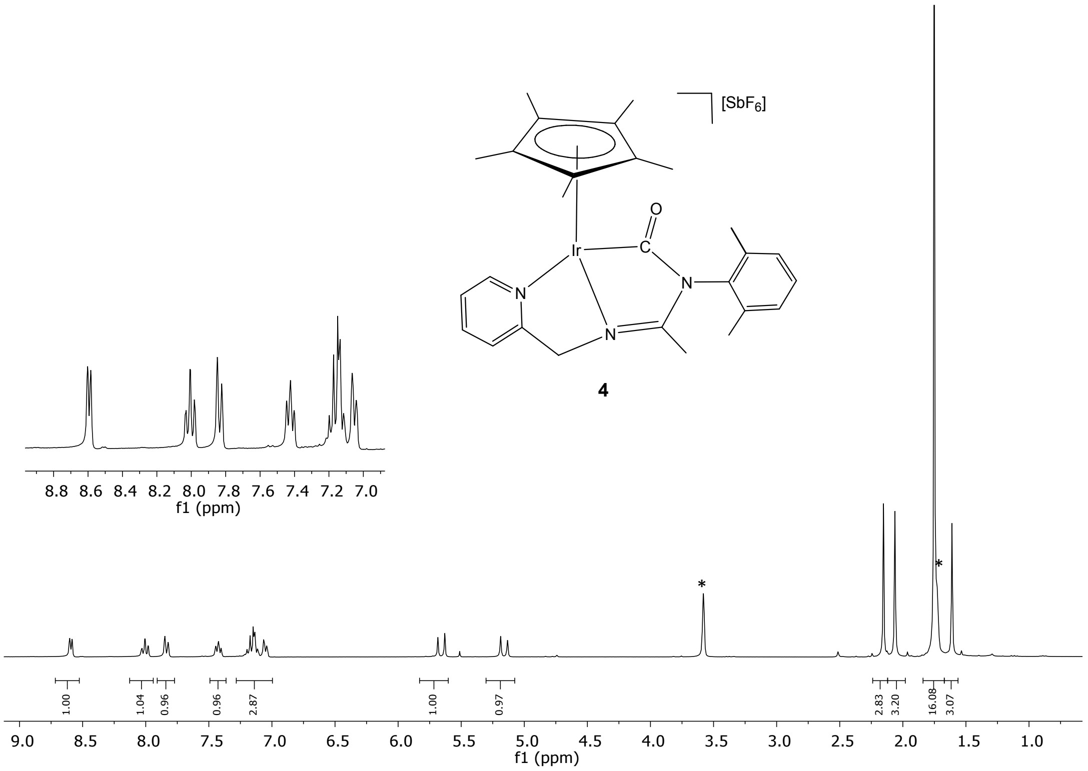

Fig. S4. ^{13}C{^{1}H}-NMR (THF-d_8, RT) spectrum of **4**

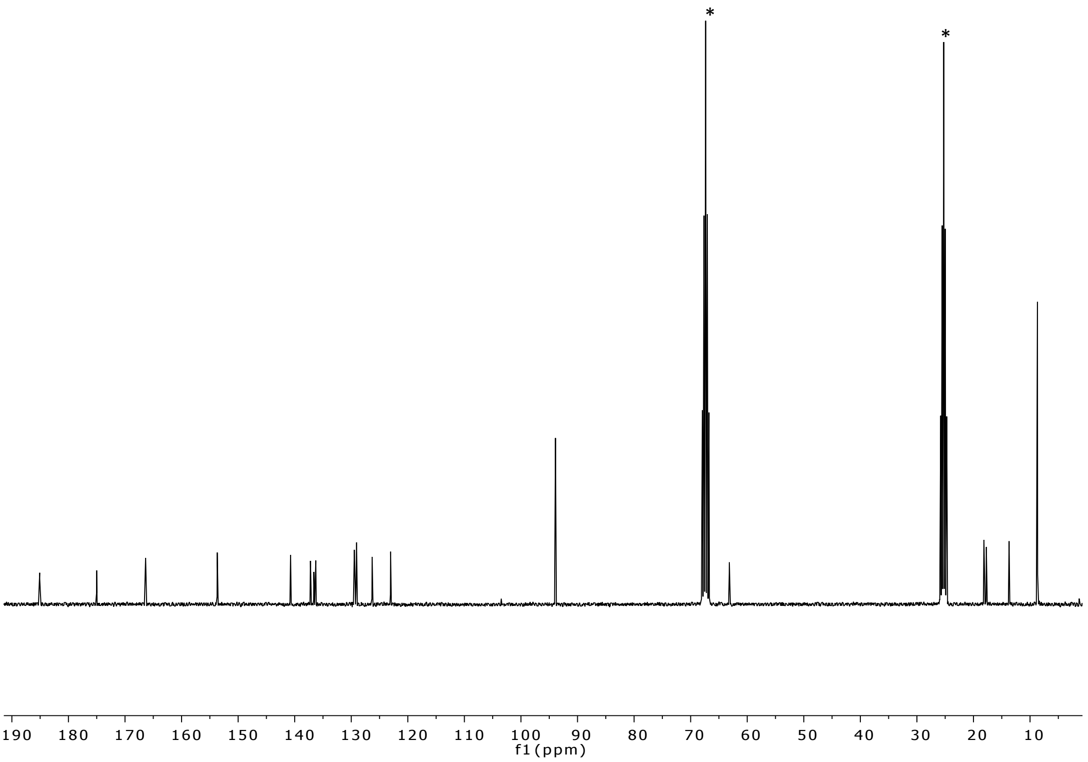

Fig. S5. ^{1}H-NMR (CD_2Cl_2, RT) spectrum of **5**

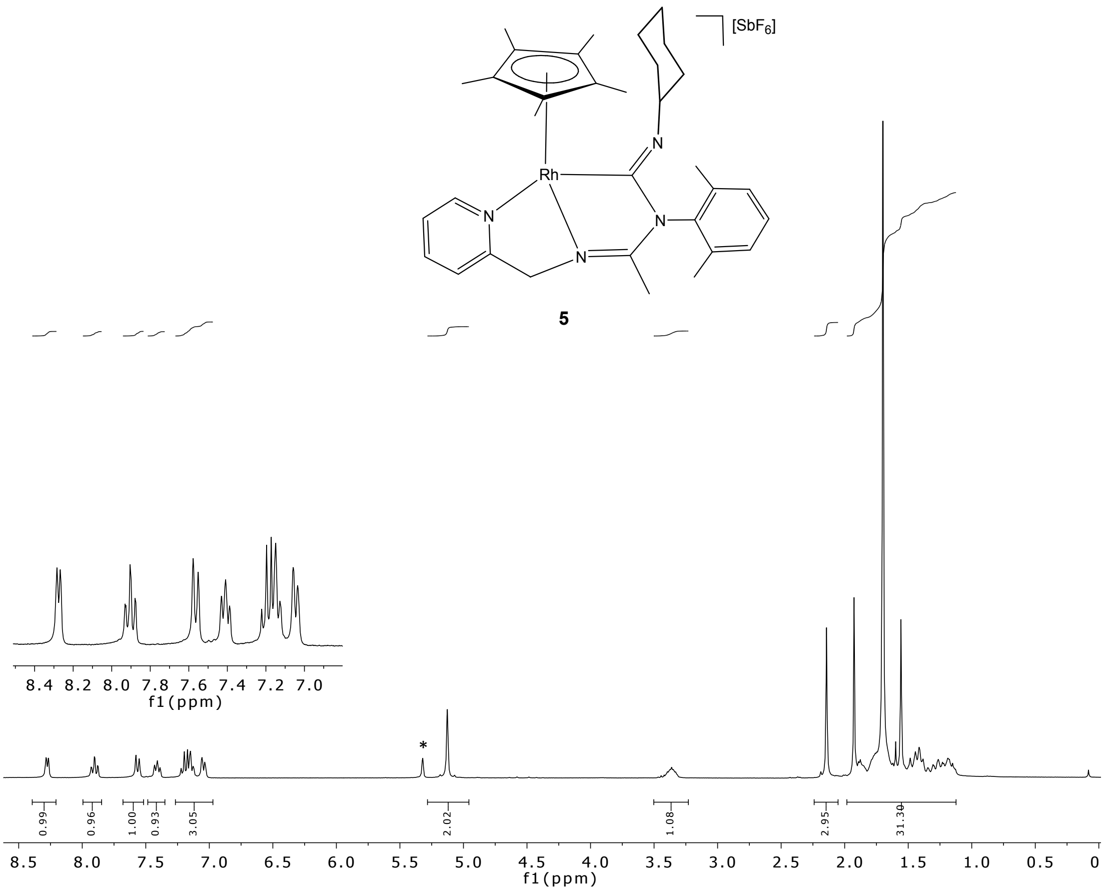

Fig. S6. $^{13}C\{^{1}H\}$-NMR (CD_2Cl_2, RT) spectrum of **5**

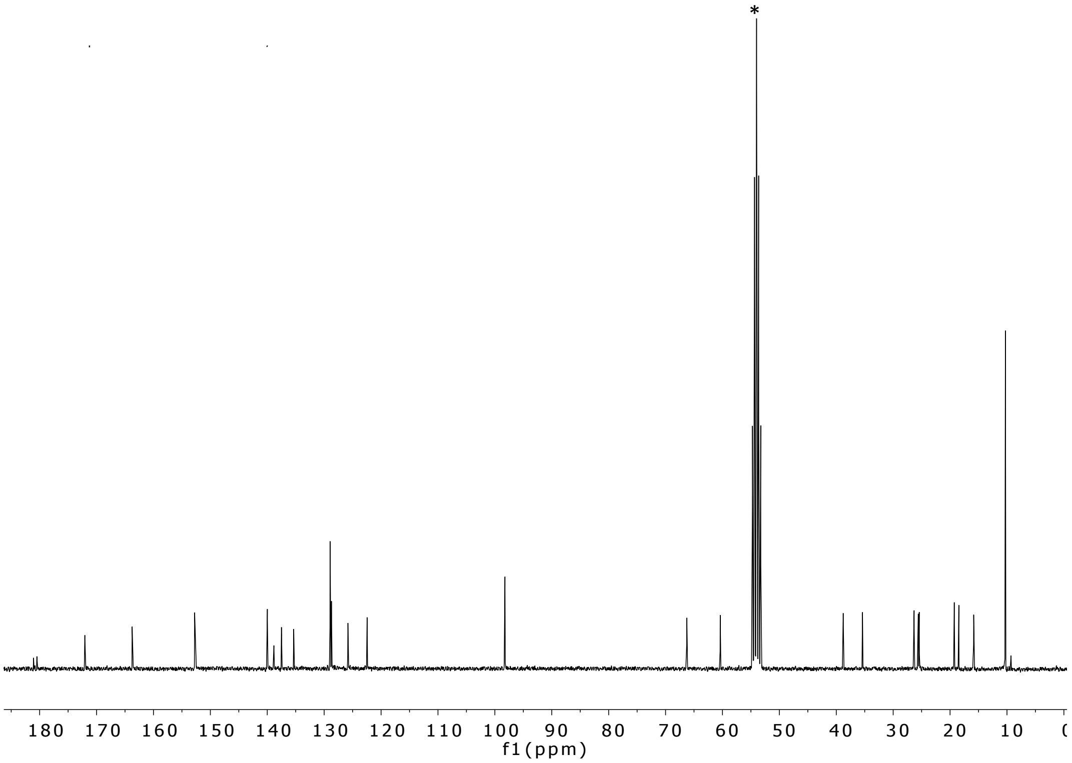

Fig. S7. ^{1}H-NMR (CD_2Cl_2, RT) spectrum of **6**

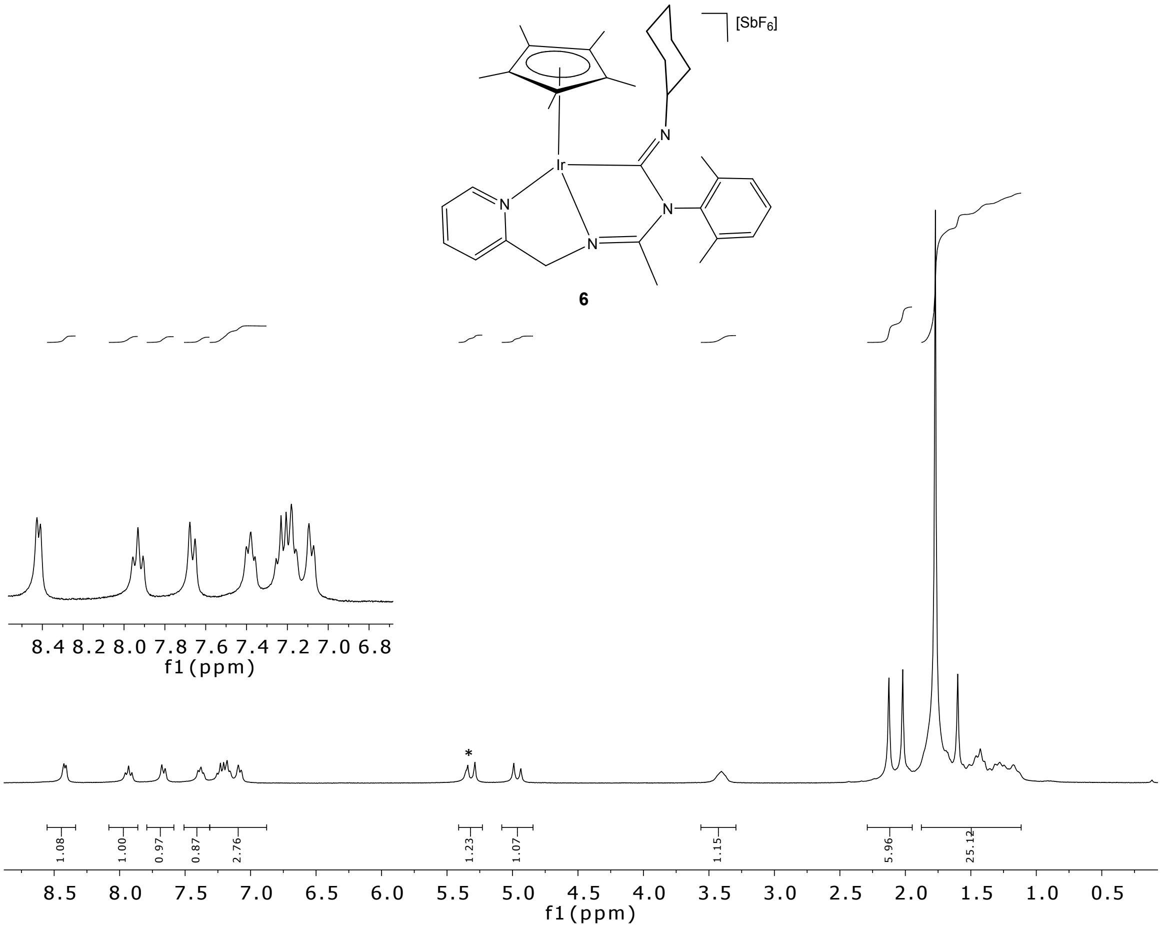

Fig. S8. $^{13}C\{^{1}H\}$-NMR (CD_2Cl_2, RT) spectrum of **6**

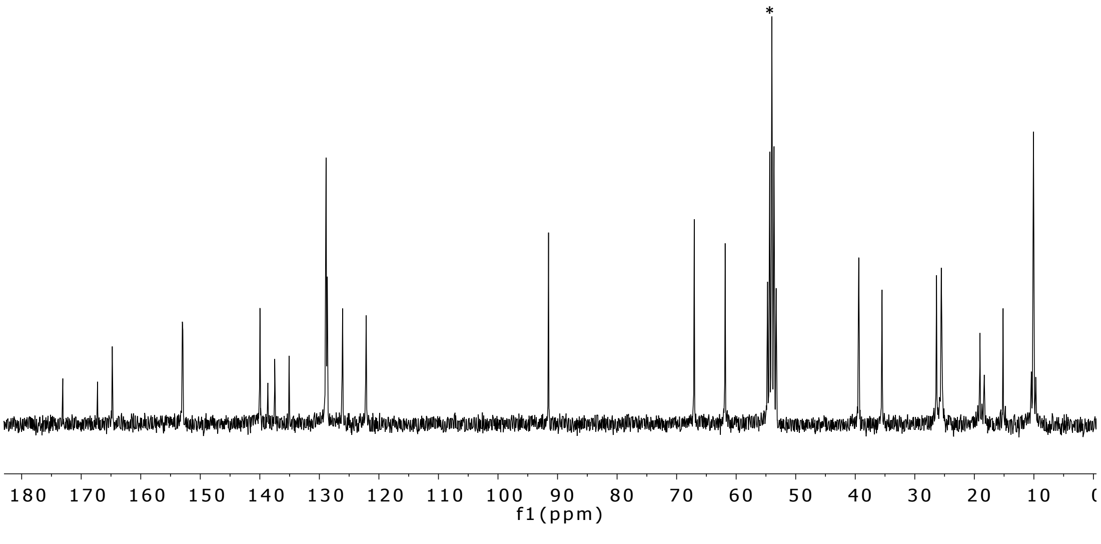

Fig. S9. ^{1}H-NMR (CD_2Cl_2, RT) spectrum of **7**

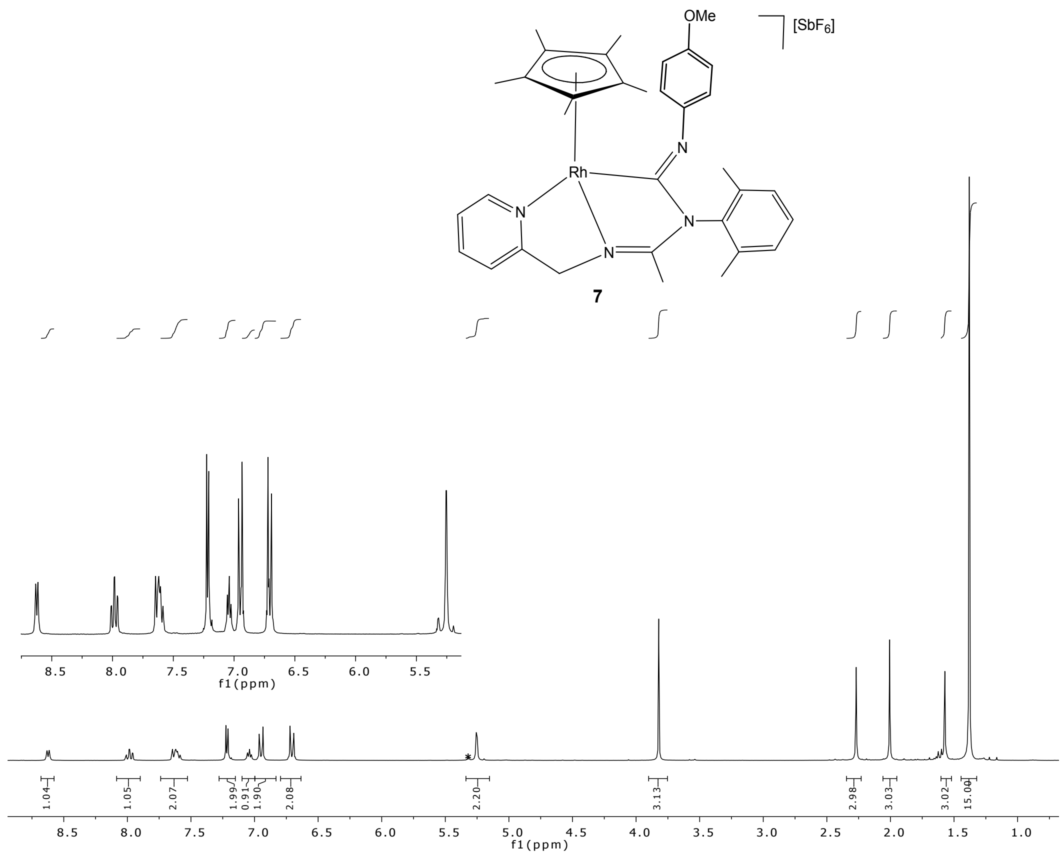

Fig. S10. ^{13}C{^{1}H}-NMR (CD_2Cl_2, RT) spectrum of **7**

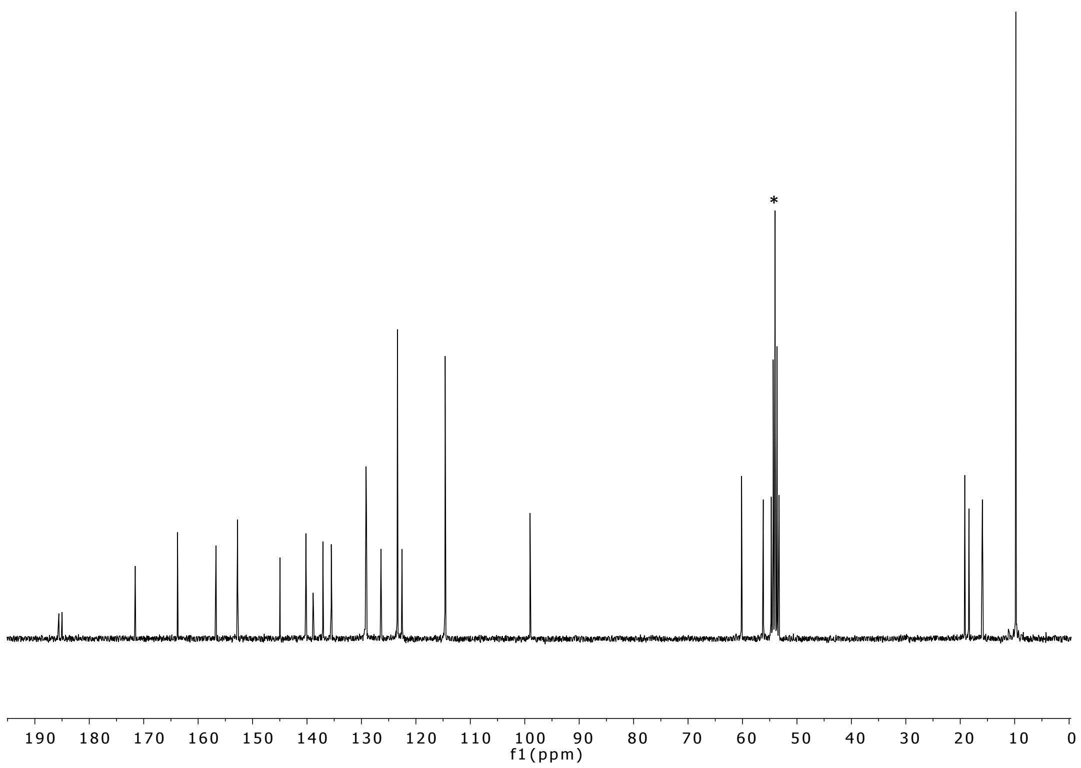

Fig. S11. ^{1}H-NMR (CD_2Cl_2, RT) spectrum of **8**

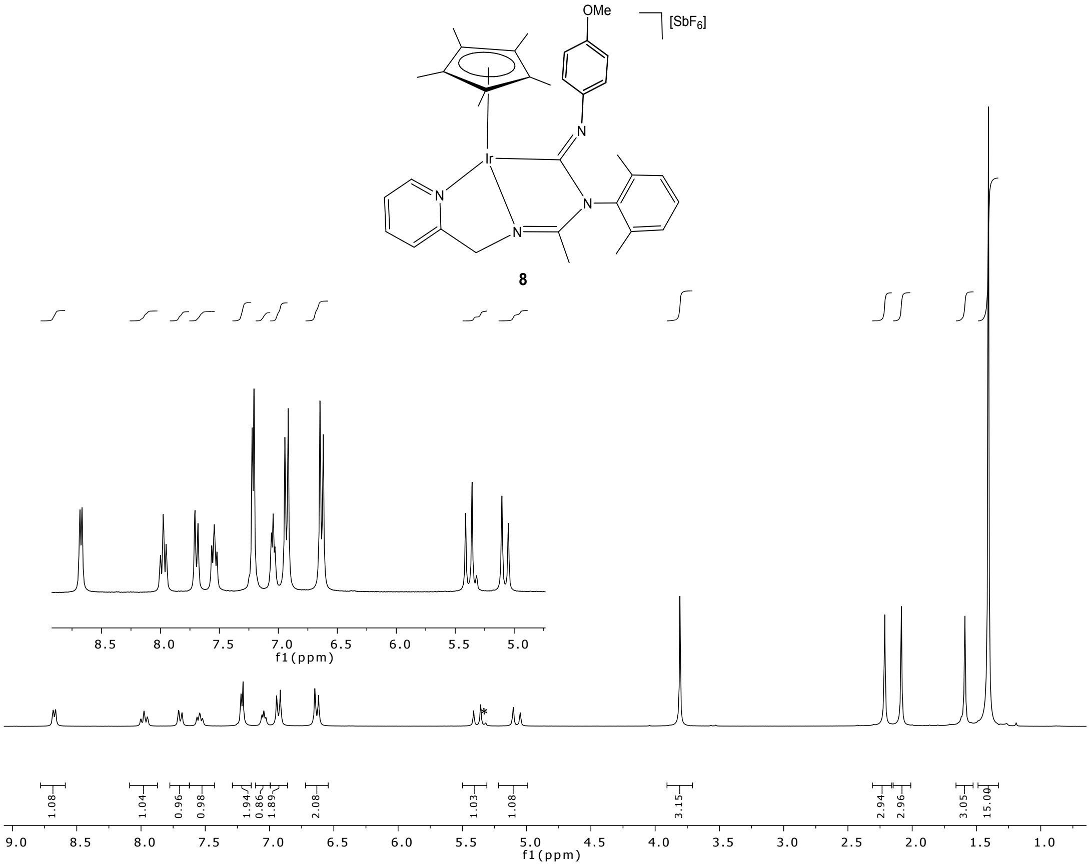

Fig. S12. $^{13}C\{^{1}H\}$-NMR (CD_2Cl_2, RT) spectrum of **8**

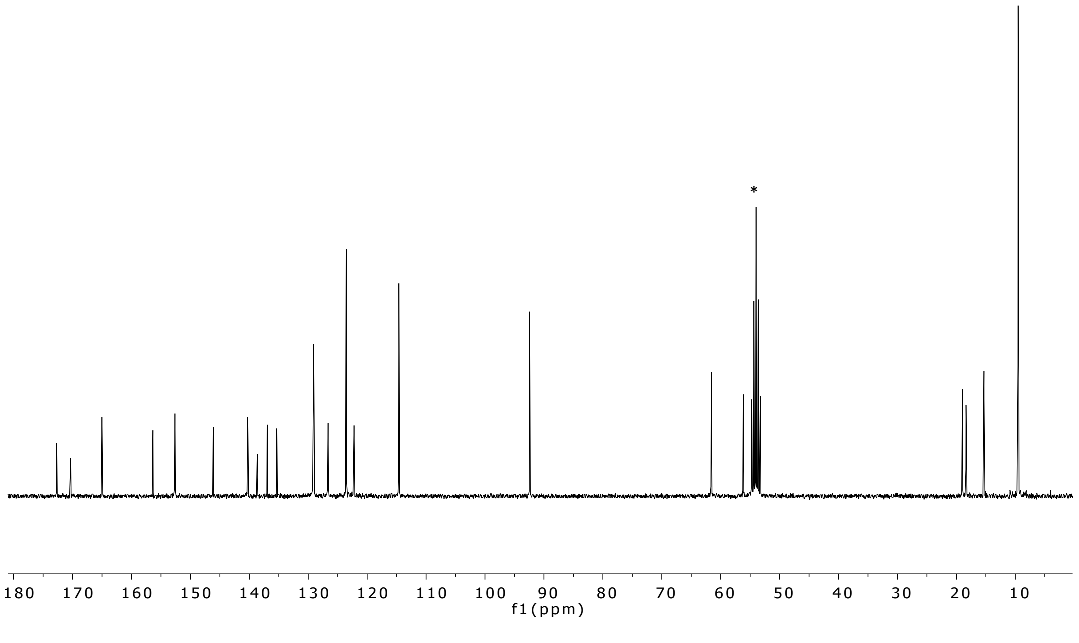

Fig. S13. ^{1}H-NMR (CD_2Cl_2, RT) spectrum of **9**

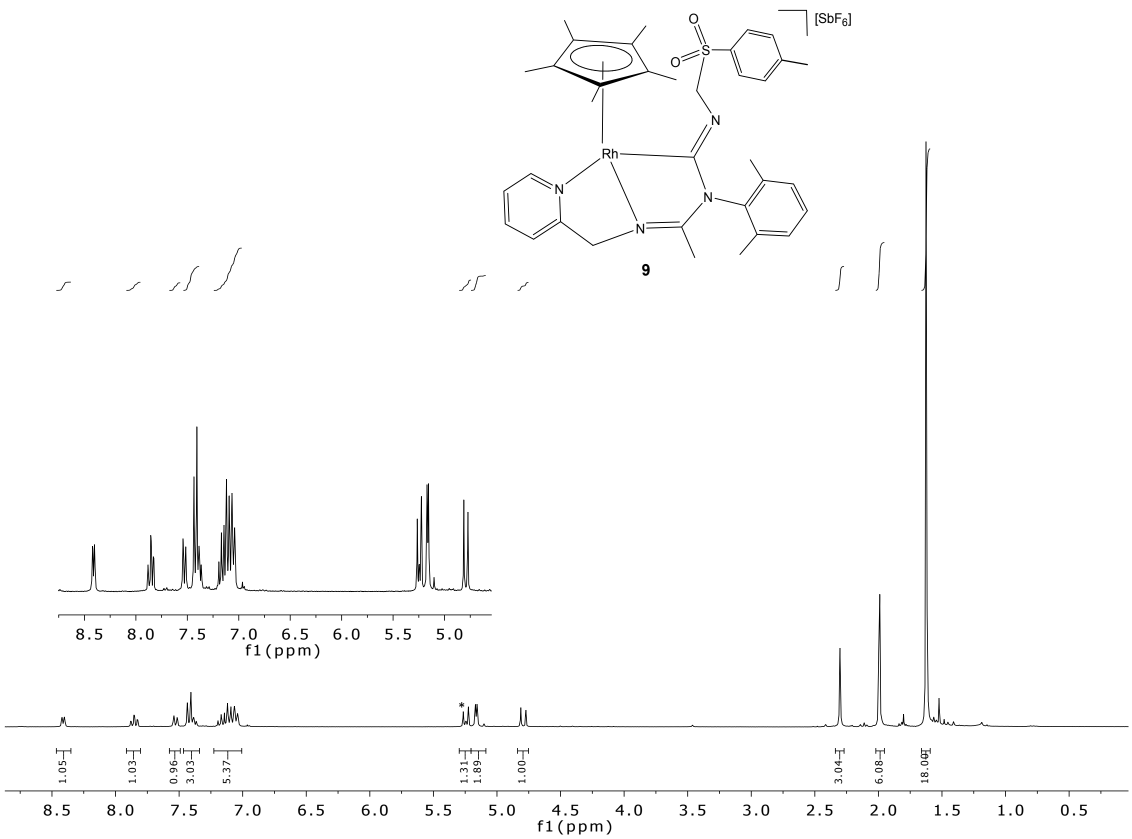

Fig. S14. ^{13}C{^{1}H}-NMR (CD_2Cl_2, RT) spectrum of **9**

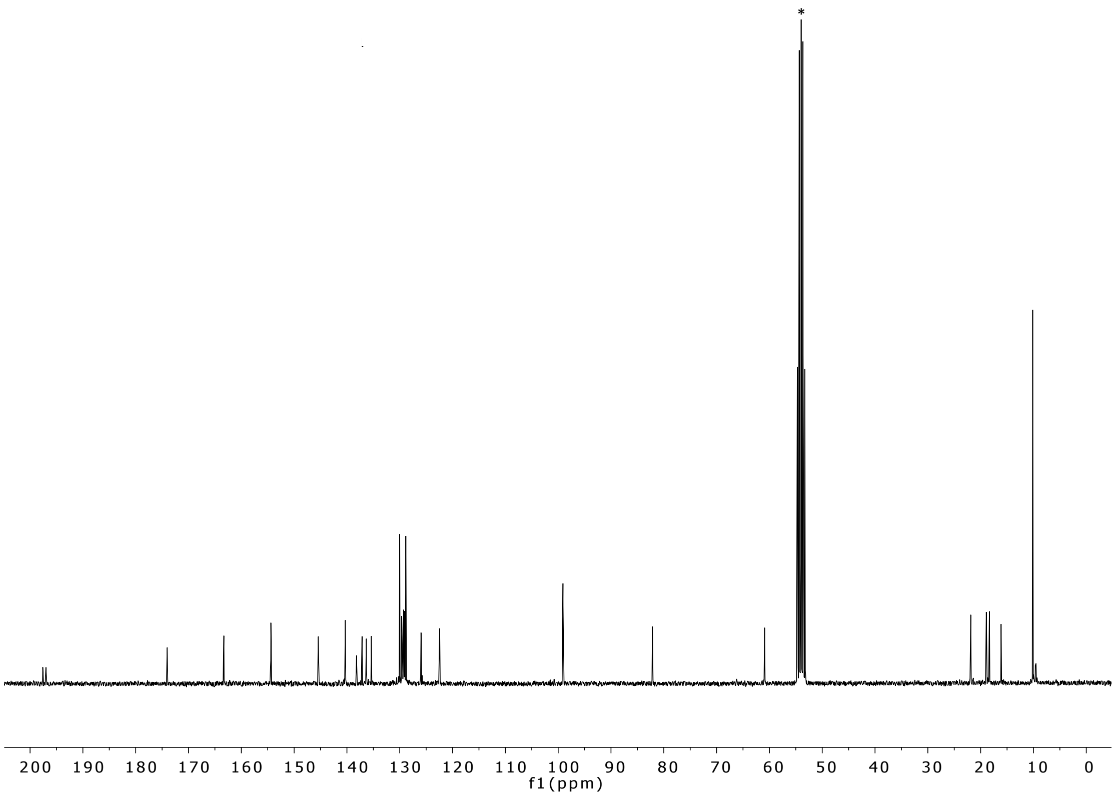

Fig. S15. ^{1}H-NMR (CD_2Cl_2, RT) spectrum of **10**

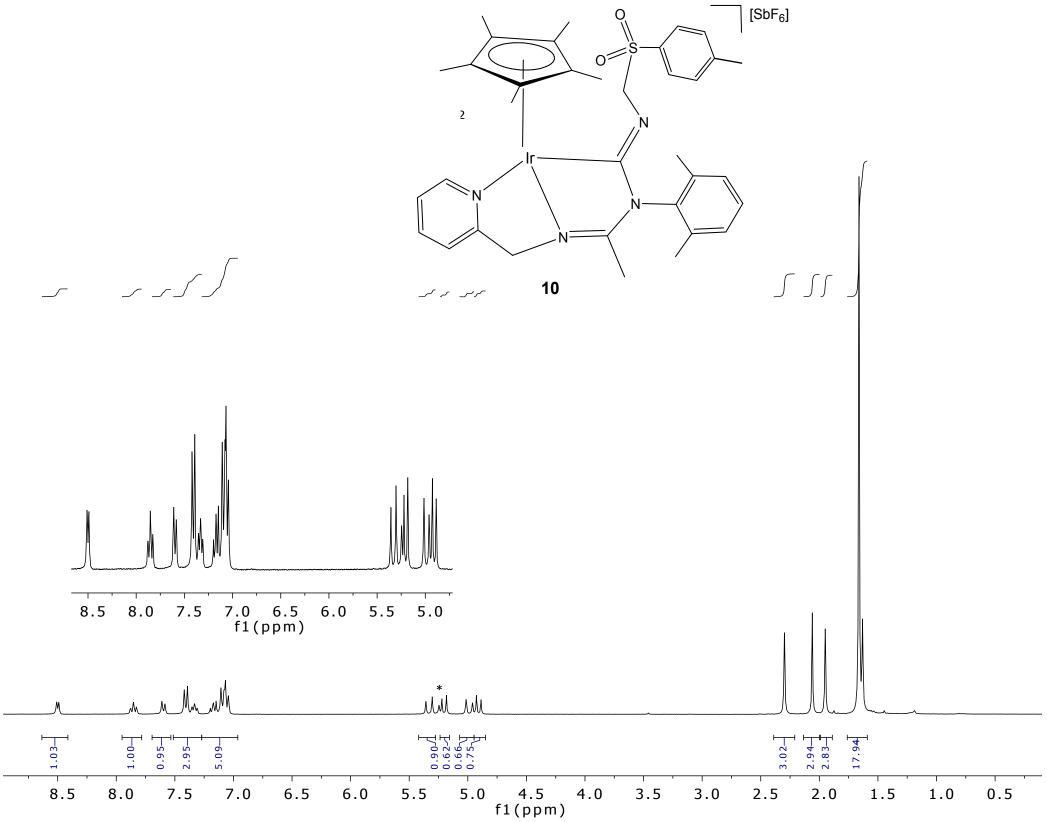

Fig. S16. ^{13}C{^{1}H}-NMR (CD_2Cl_2, RT) spectrum of **10**

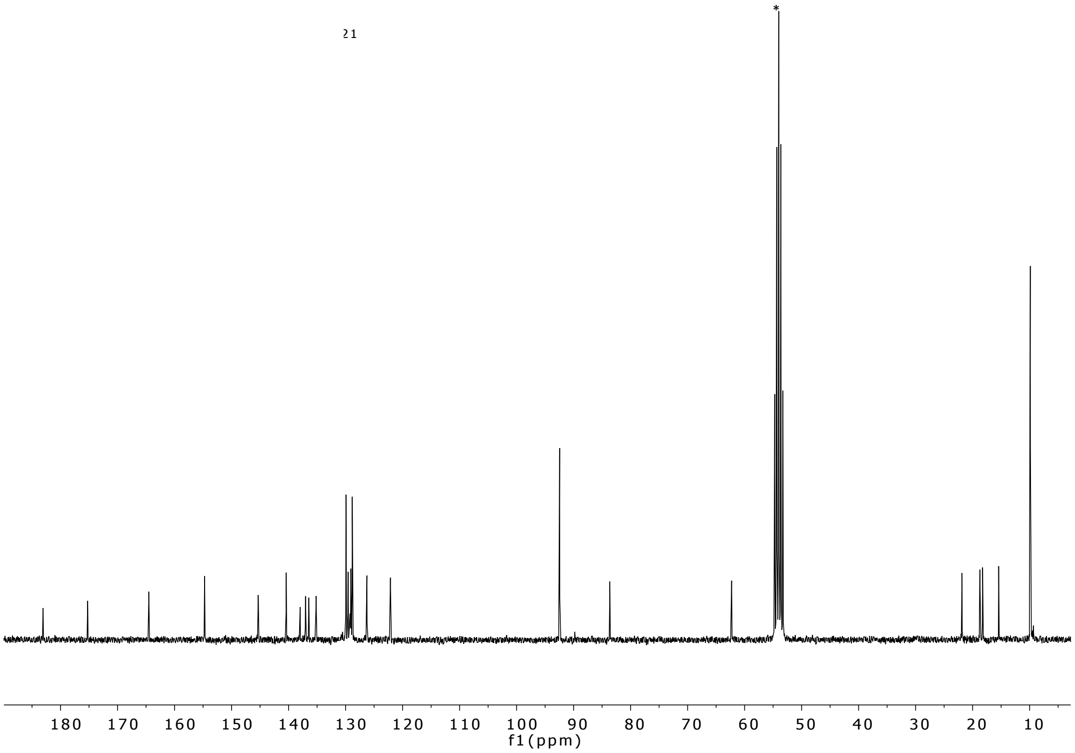

Fig. S17. ^{1}H-NMR (CD_2Cl_2, RT) spectrum of **11**

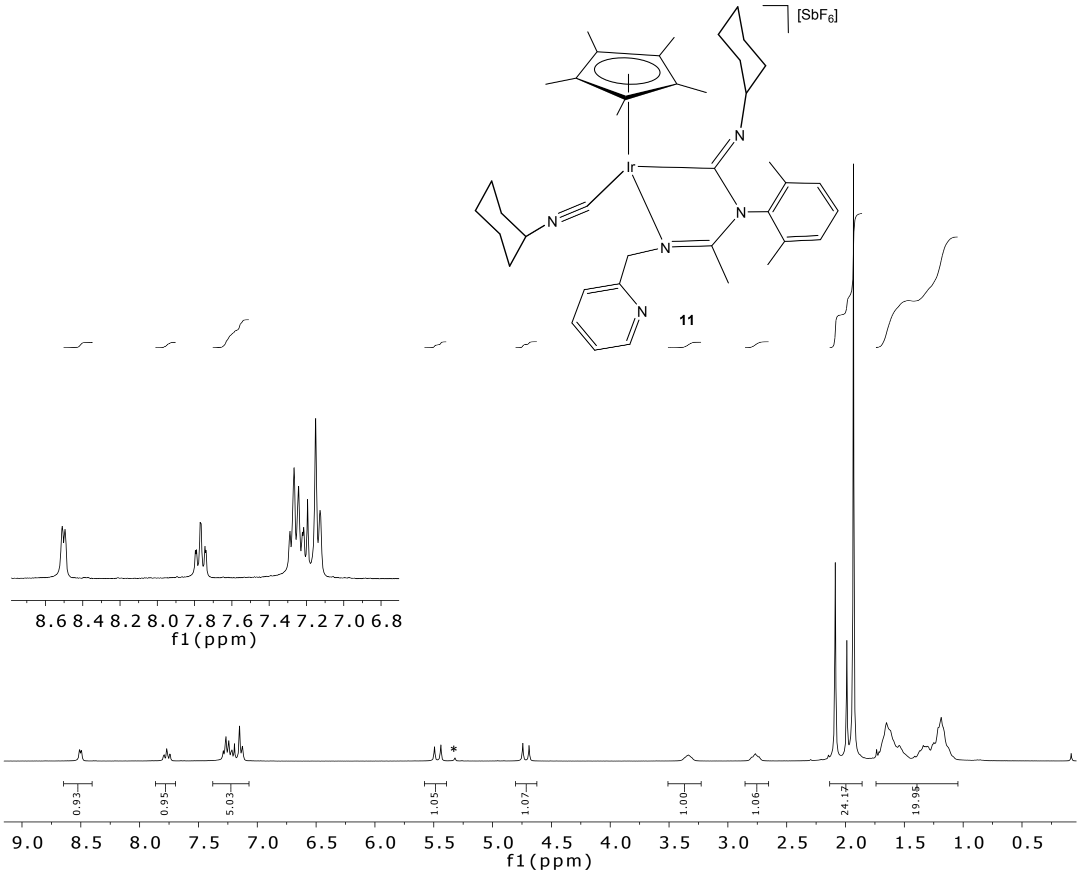

Fig. S18. ^{13}C{^{1}H}-APT (CD_2Cl_2, RT) spectrum of **11**

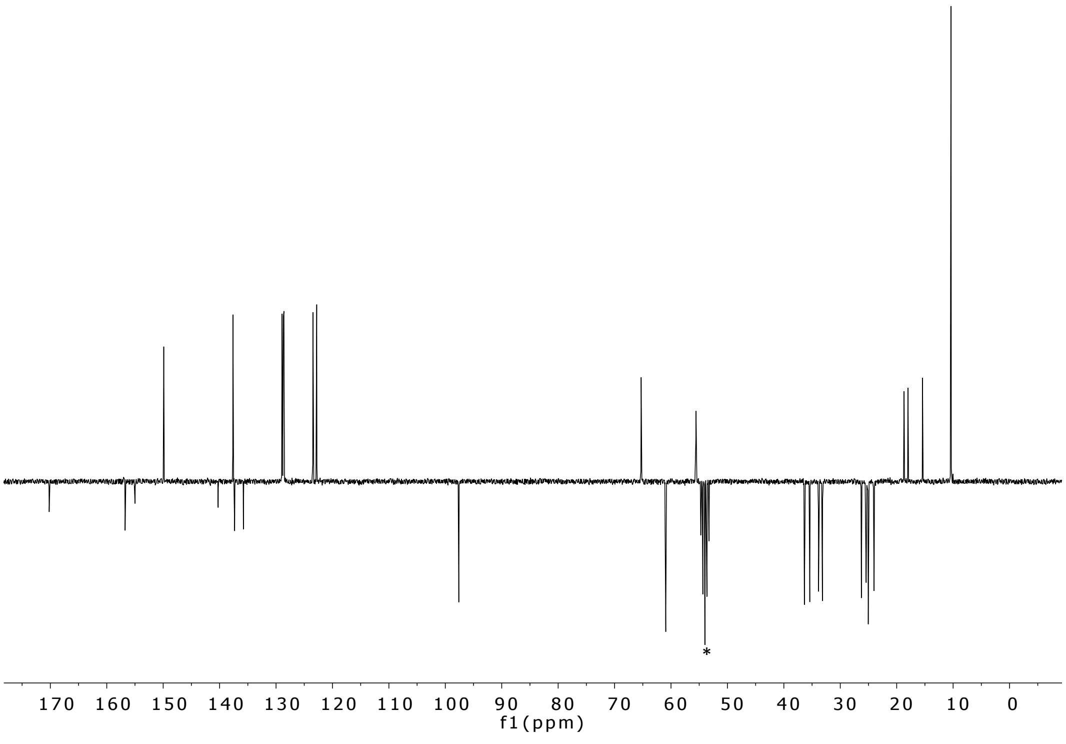

Fig. S19. ^{1}H-NMR (CD_2Cl_2, RT) spectrum of **12**

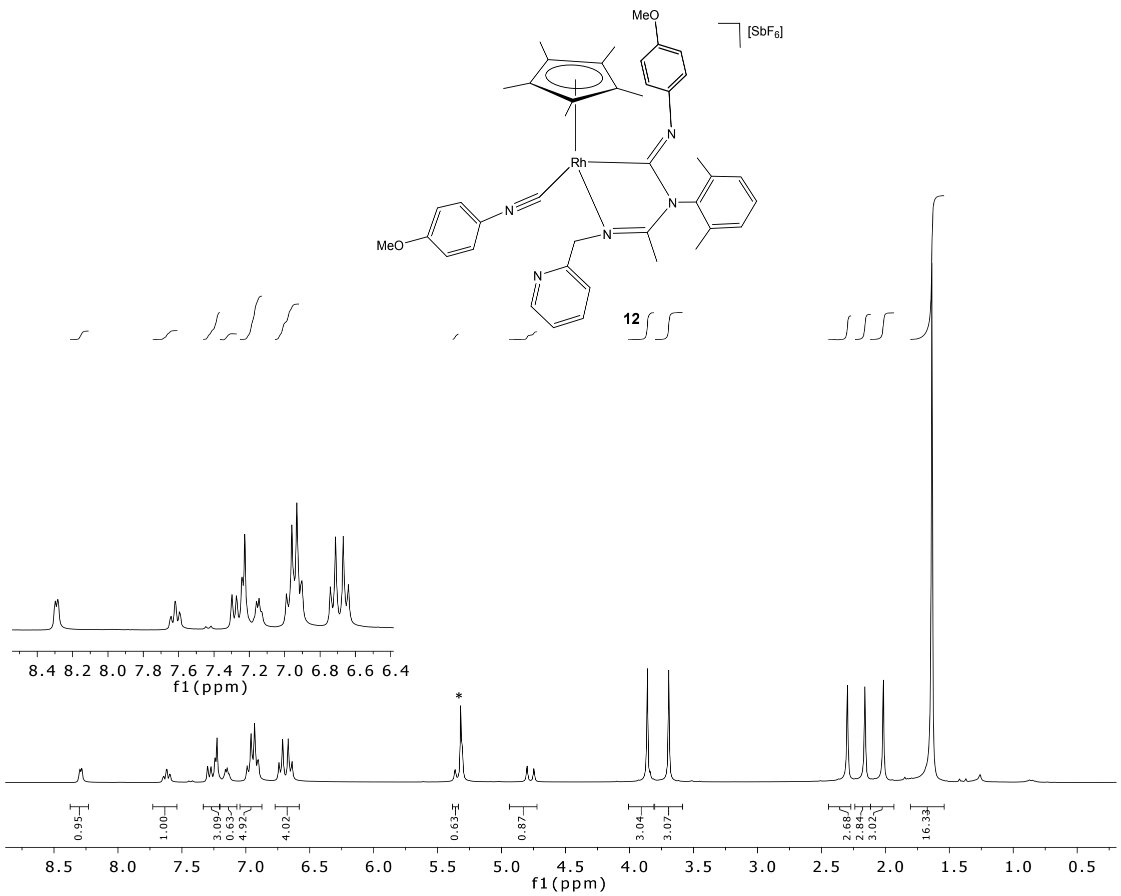

Fig. S20. ^{13}C{^{1}H}-NMR (CD_2Cl_2, RT) spectrum of **12**

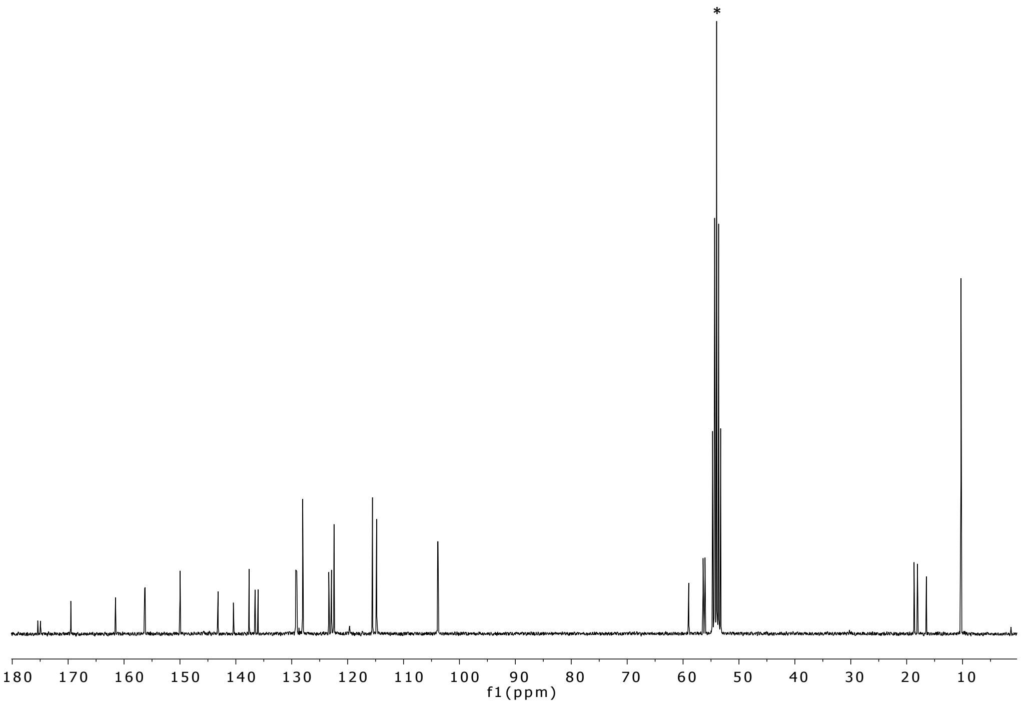

Fig. S21. ^{1}H-NMR (CD_2Cl_2, RT) spectrum of **13**

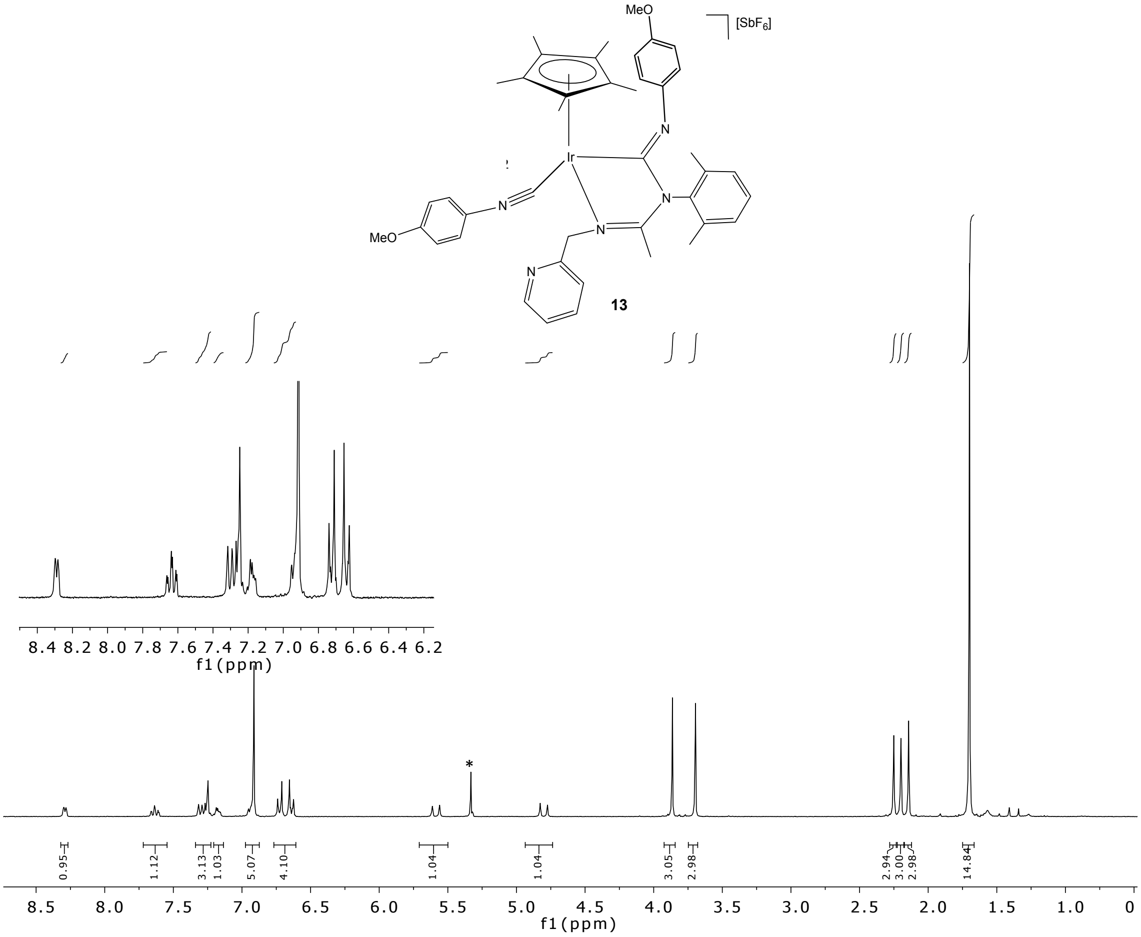

Fig. S22. $^{13}C\{^{1}H\}$-NMR (CD_2Cl_2, RT) spectrum of **13**

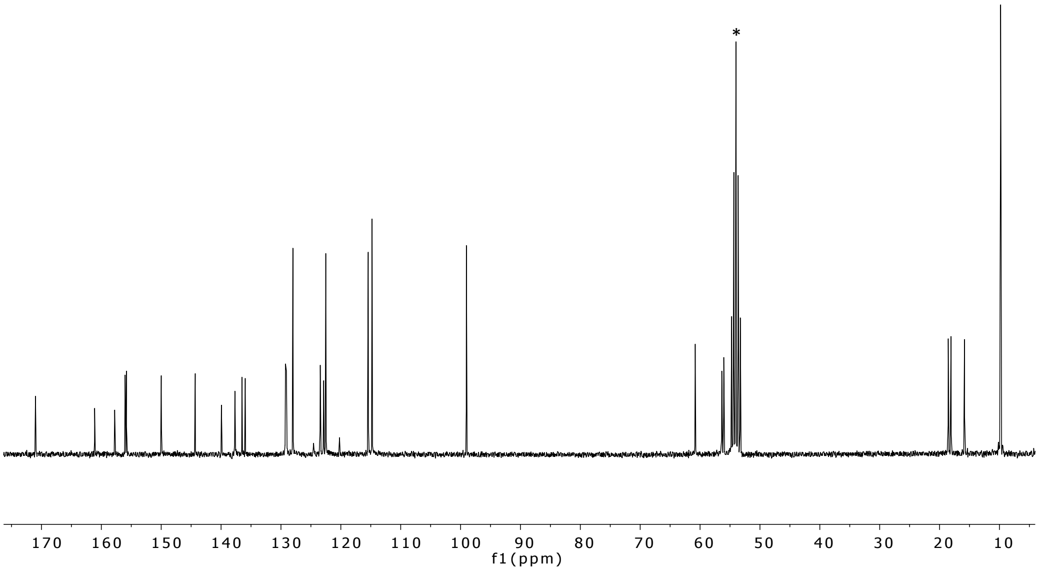

Fig. S23. ^{1}H-NMR (CD_2Cl_2, RT) spectrum of **14**

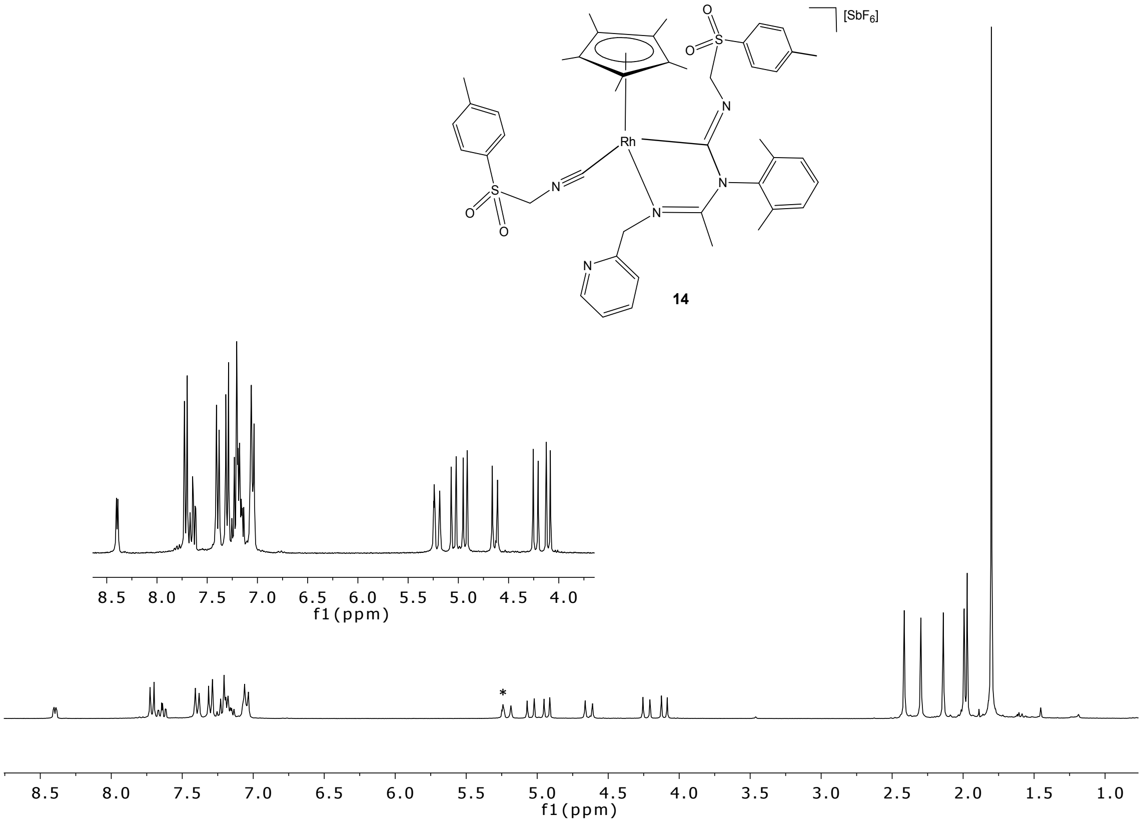

Fig. S24. ^{13}C{^{1}H}-NMR (CD_2Cl_2, RT) spectrum of **14**

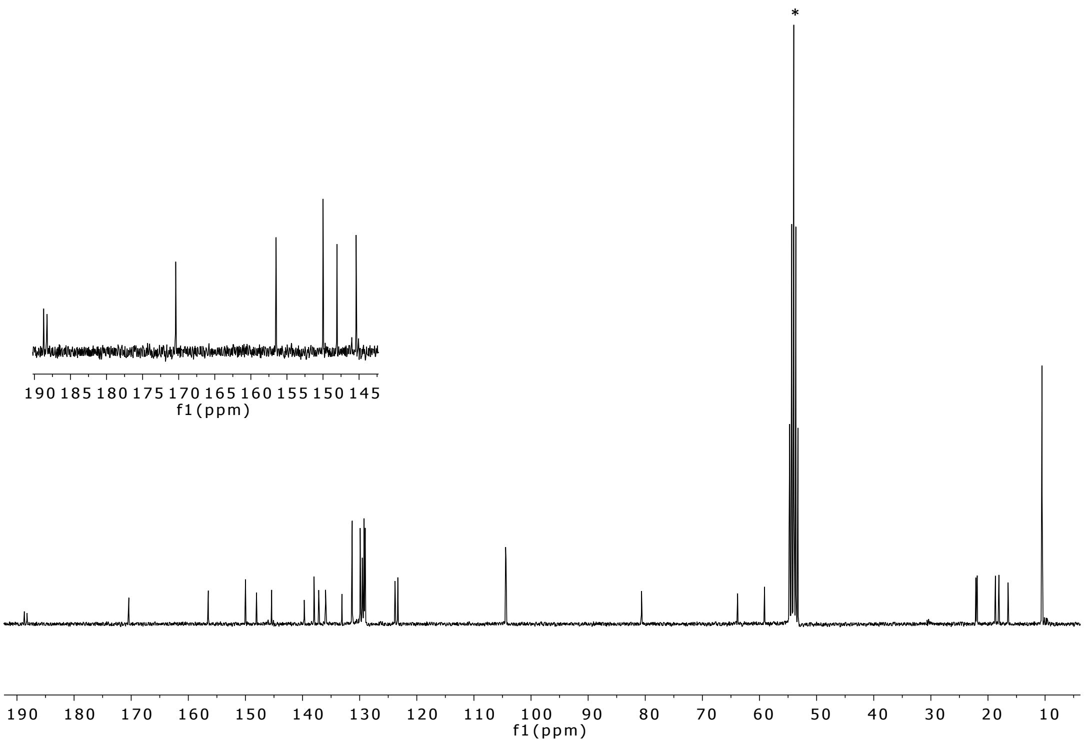

Fig. S25. ^{1}H-NMR (CD_2Cl_2, RT) spectrum of **15**

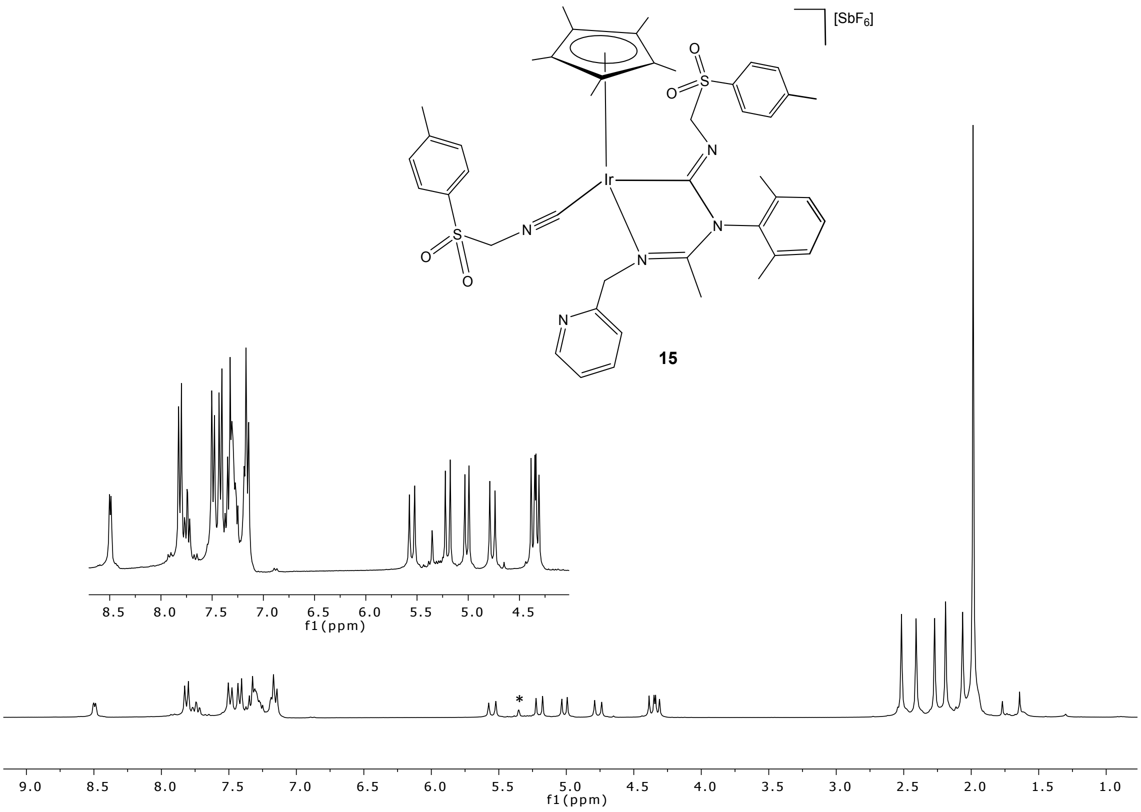

Fig. S26. ^{13}C{^{1}H}-APT (CD_2Cl_2, RT) spectrum of **15**

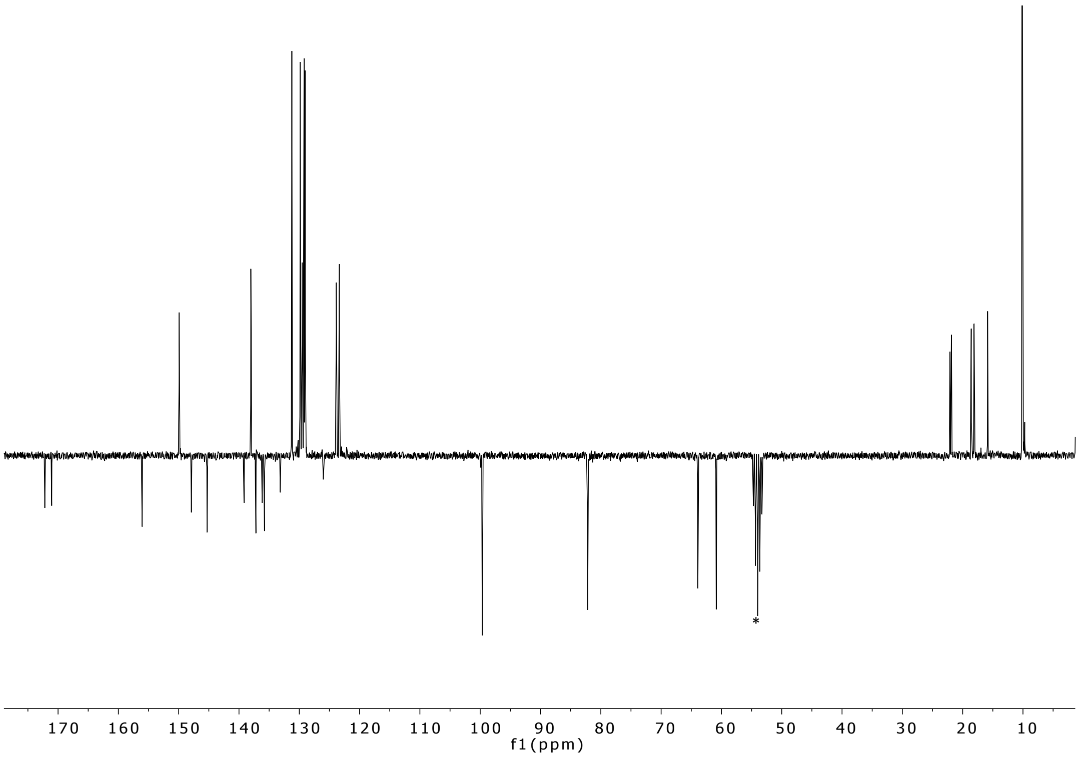

Fig. S27. ^{1}H-NMR (CD_2Cl_2, RT) spectrum of **16**

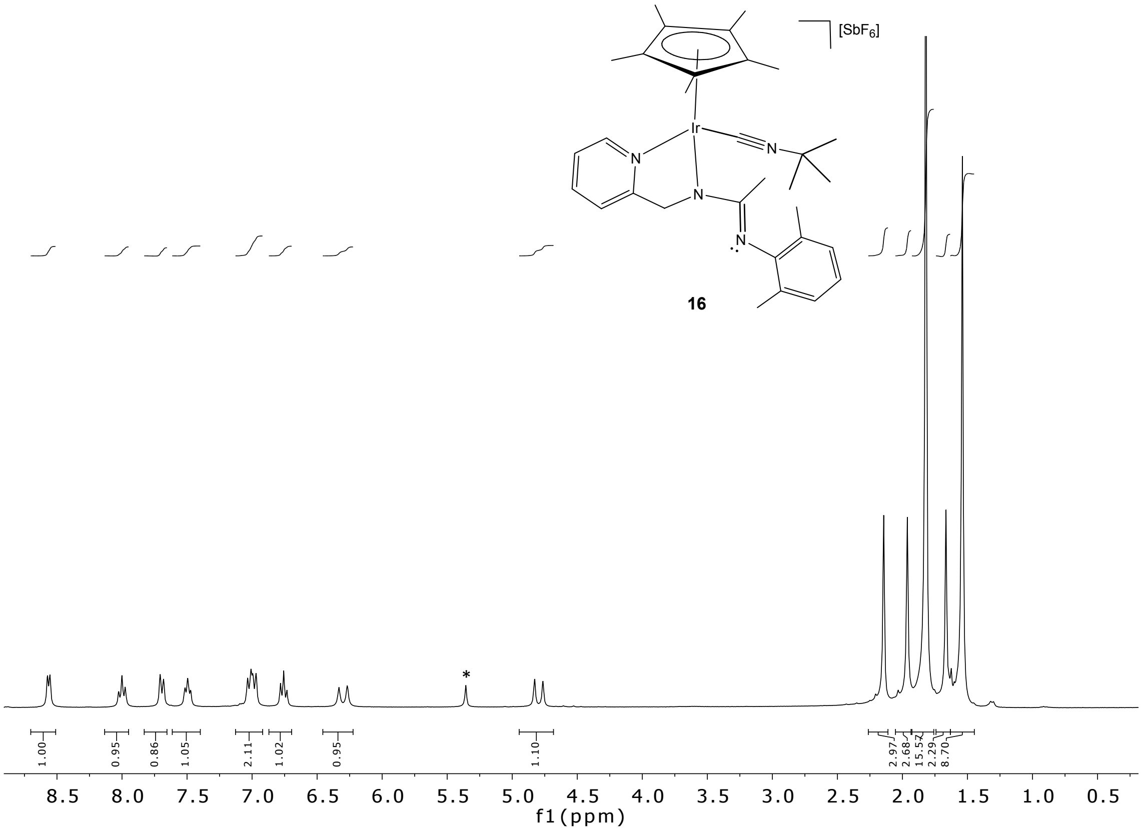

Fig. S28. ^{13}C{^{1}H}-NMR (CD_2Cl_2, RT) spectrum of **16**

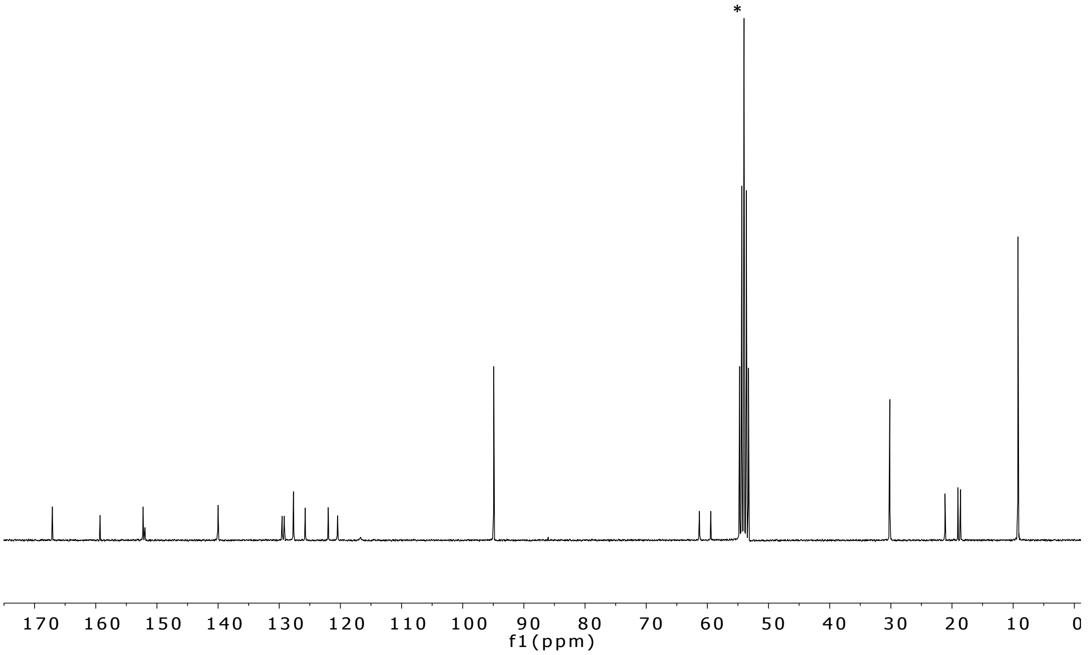

Fig. S29. ^{1}H-NMR (acetone-d_6, RT) spectrum of **17**

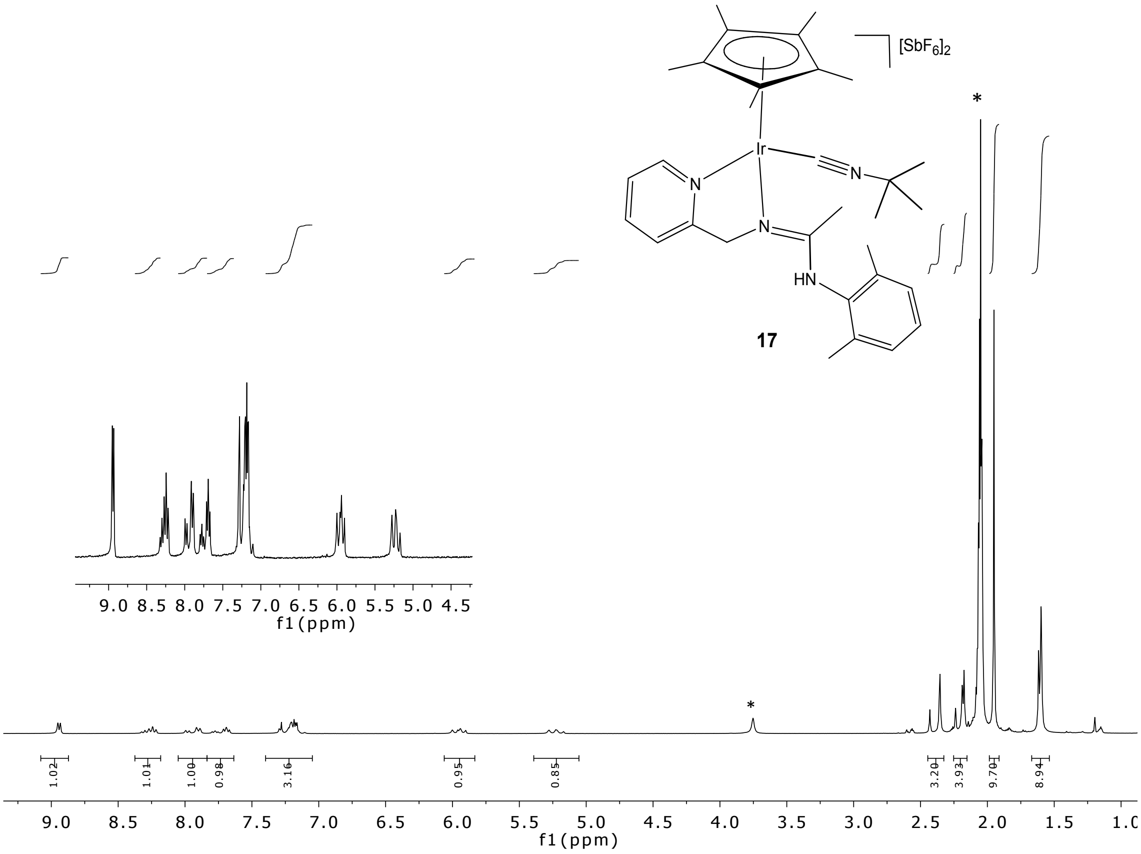

Fig. S30. ^{13}C{^{1}H}-NMR (acetone-d_6, RT) spectrum of **17**

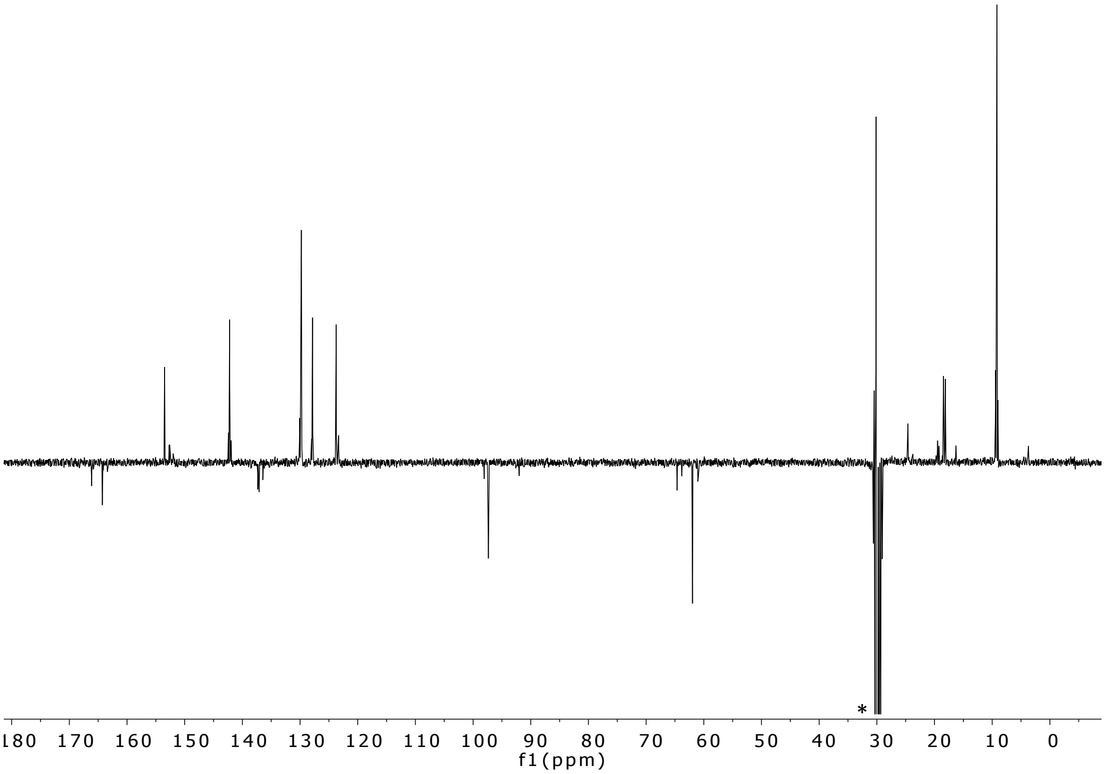

Fig. S31. ^{1}H-NMR (CD_2Cl_2, RT) spectrum of **18**

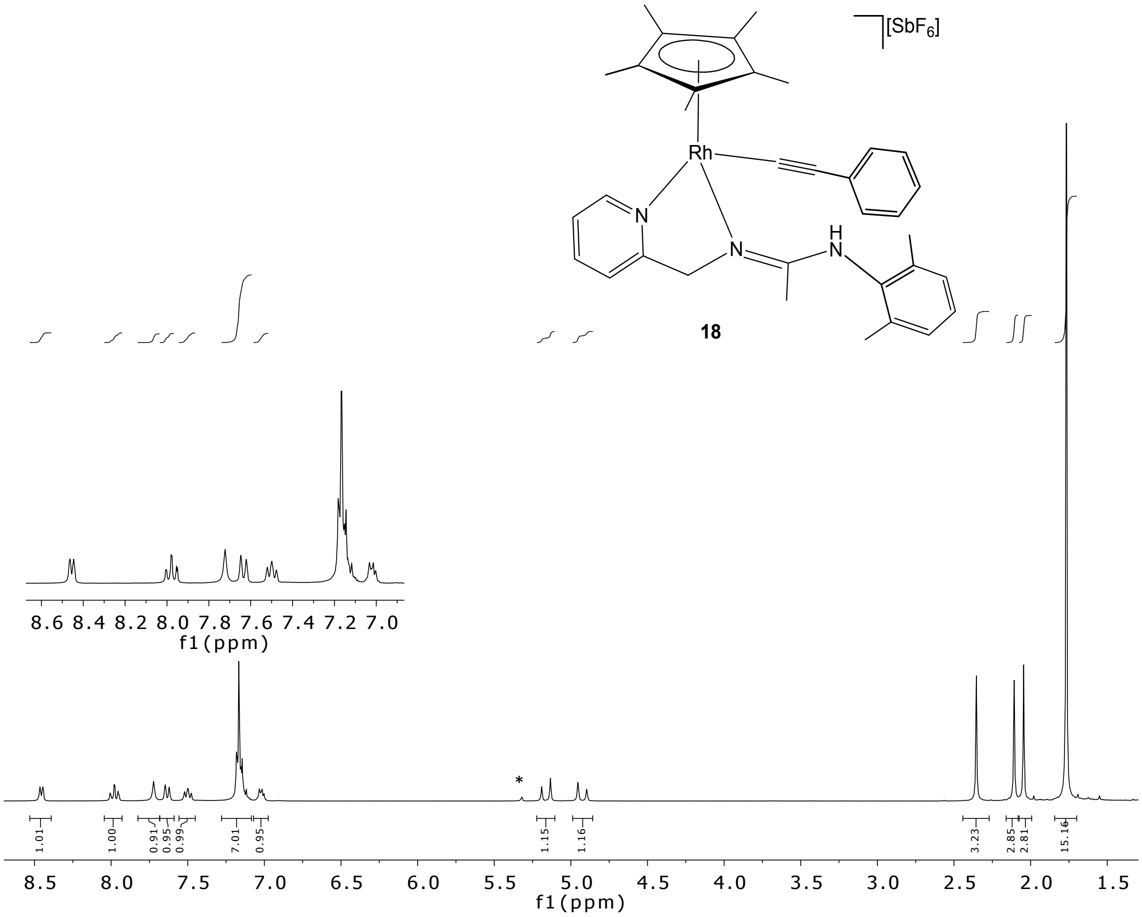

Fig. S32. ^{13}C{^{1}H}-NMR (CD_2Cl_2, RT) spectrum of **18**

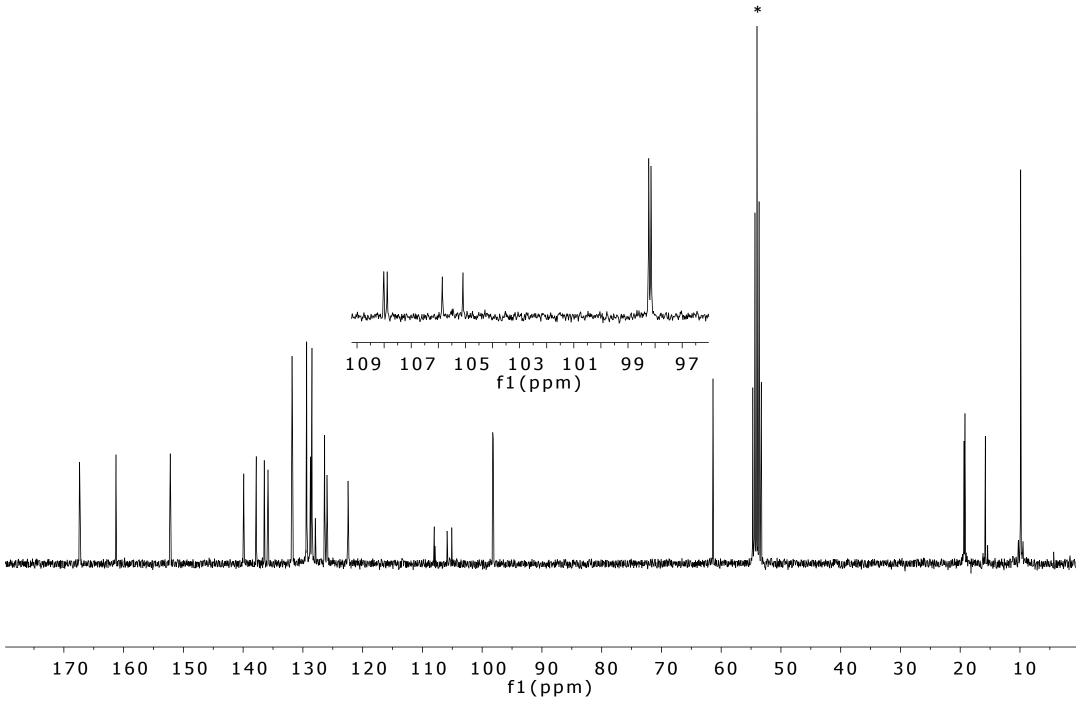

Fig. S33. ^{1}H-NMR (CD_2Cl_2, RT) spectrum of **19**

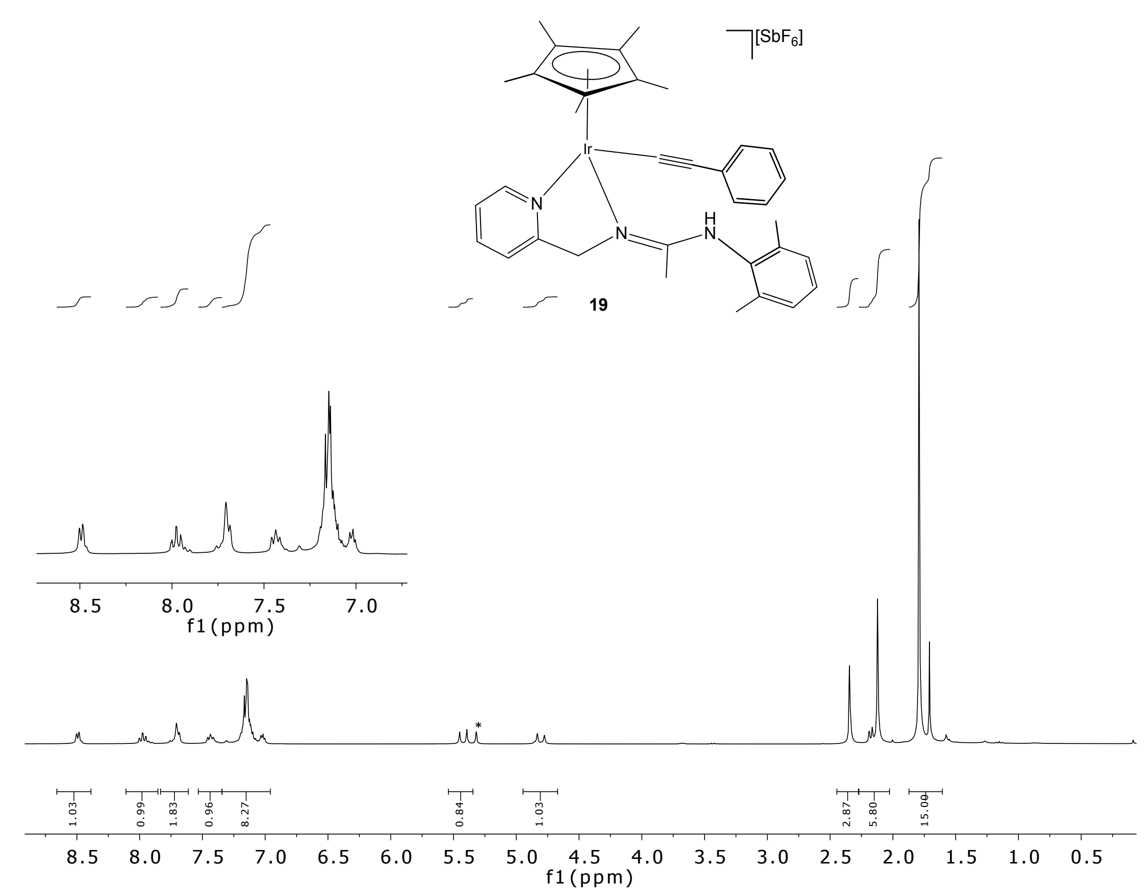

Fig. S34. $^{13}C\{^1H\}$-NMR (CD_2Cl_2, RT) spectrum of **19**

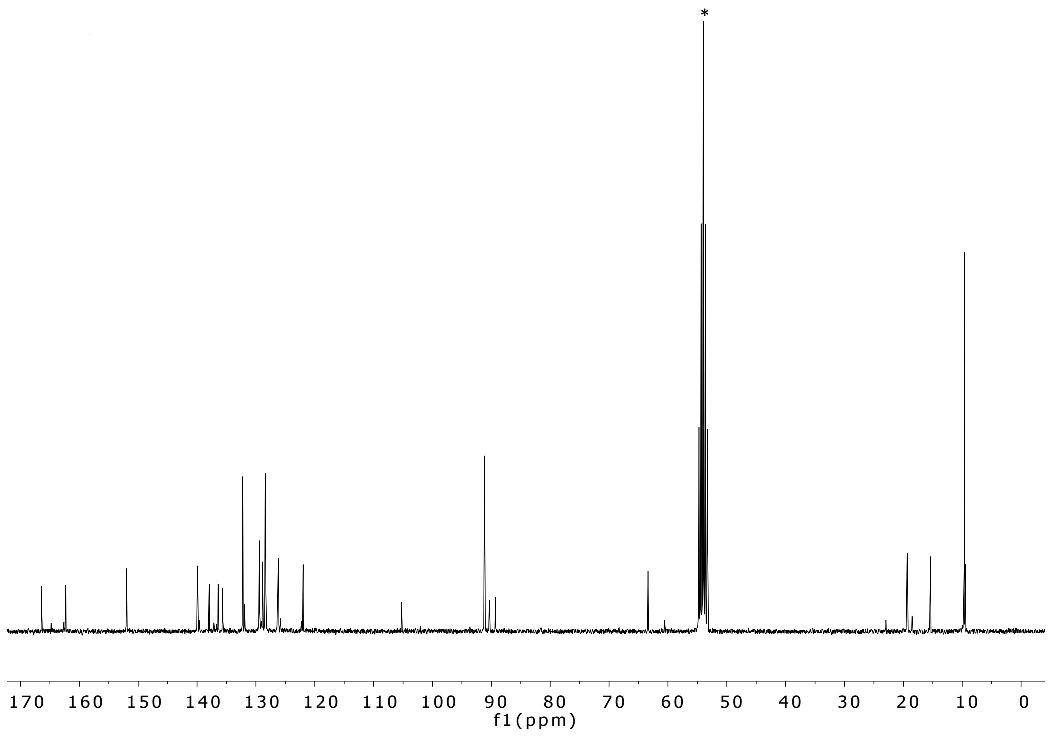

Fig. S35. ^1H-NMR (CD_2Cl_2, RT) spectrum of **20**

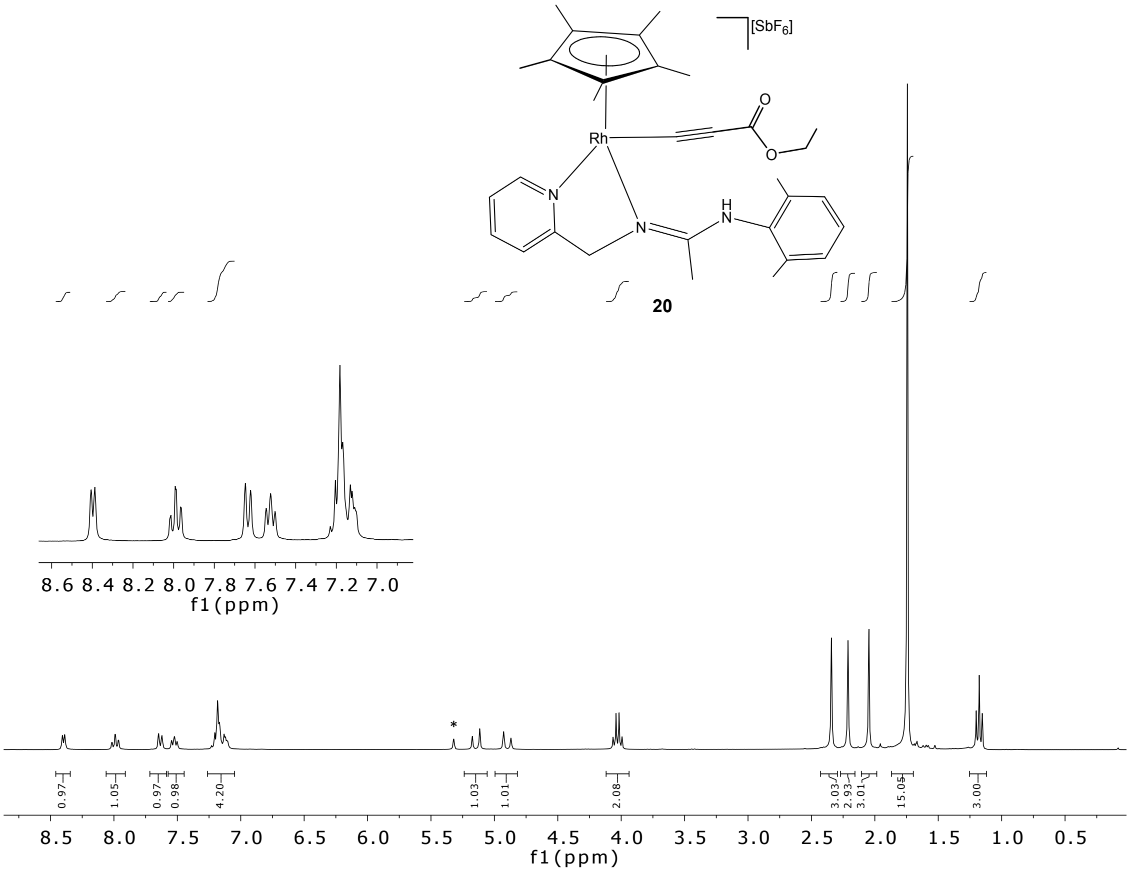

Fig. S36. $^{13}C\{^{1}H\}$-NMR (CD_2Cl_2, RT) spectrum of **20**

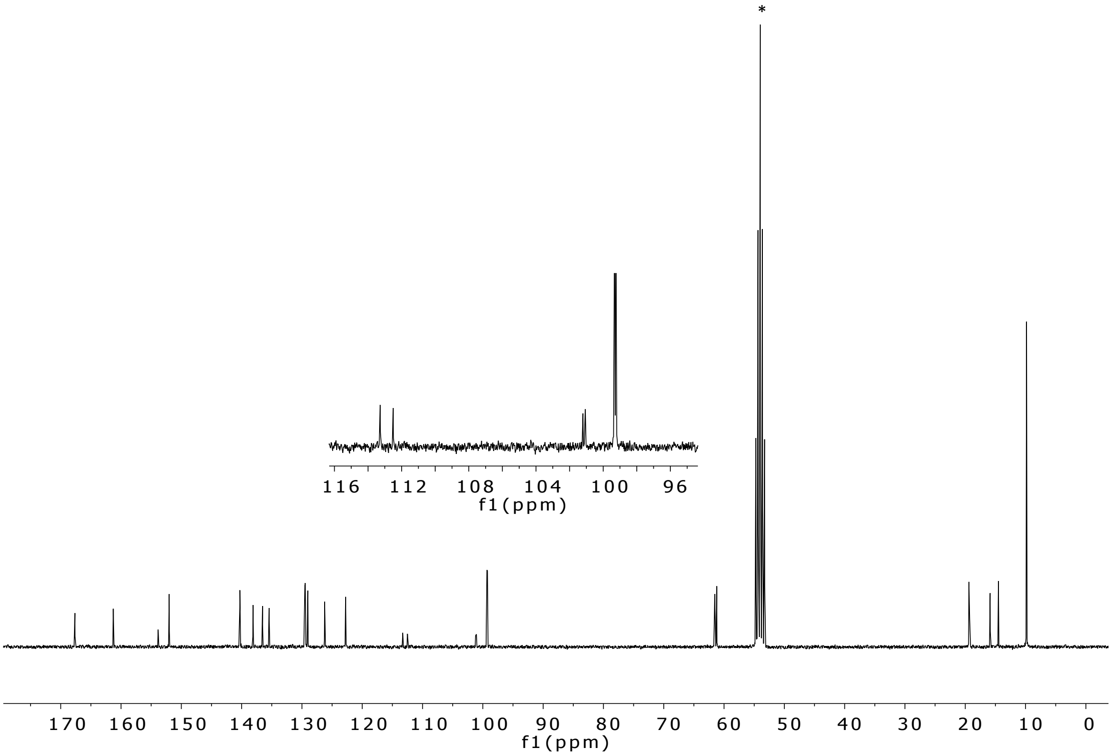

Fig. S37. ^{1}H-NMR (CD_2Cl_2, RT) spectrum of **21**

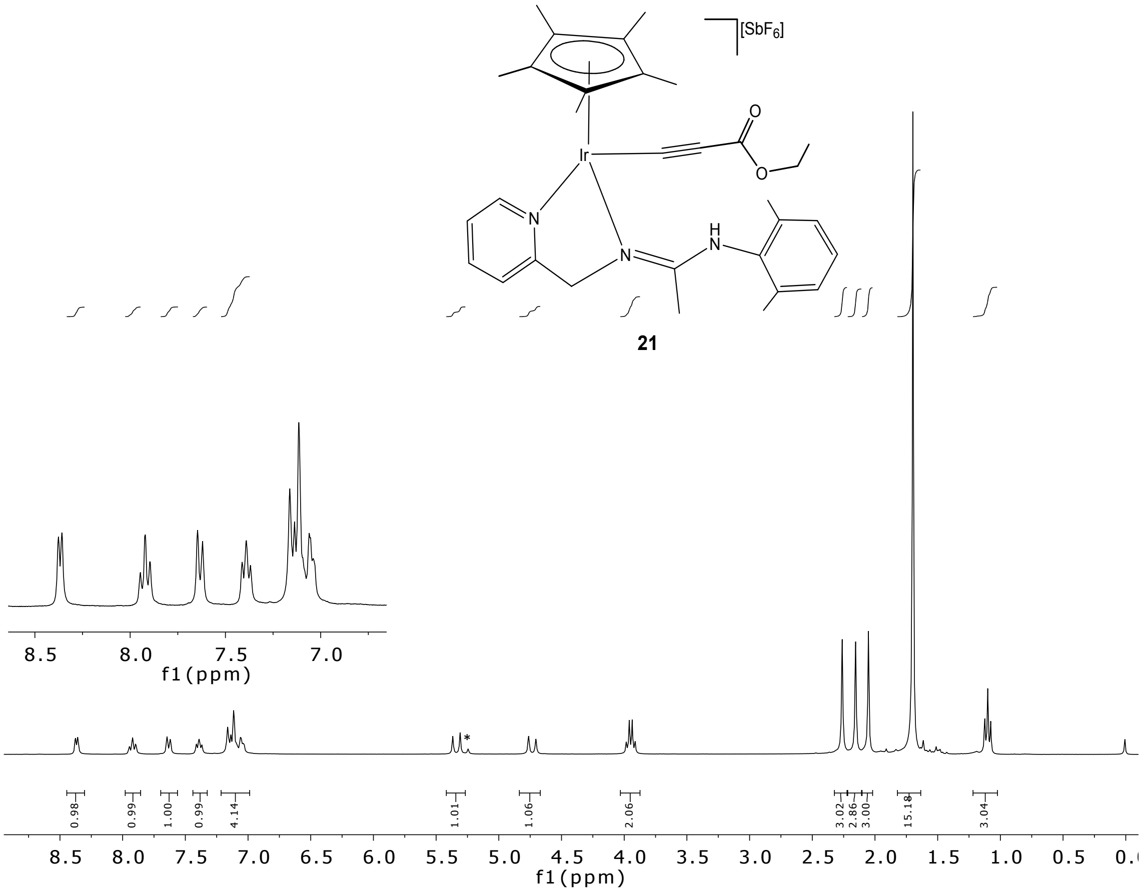

Fig. S38. $^{13}C\{^1H\}$-NMR (CD_2Cl_2, RT) spectrum of **21**

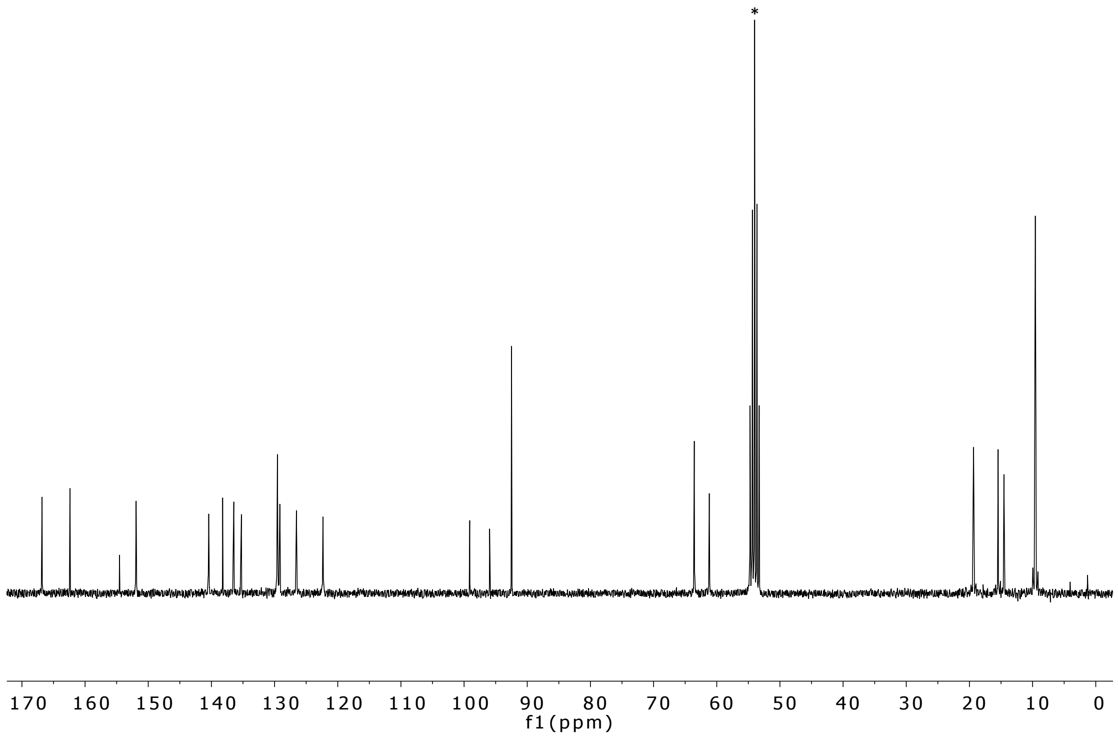

Fig. S39. ^{1}H-NMR (CD_2Cl_2, RT) spectrum of **22**

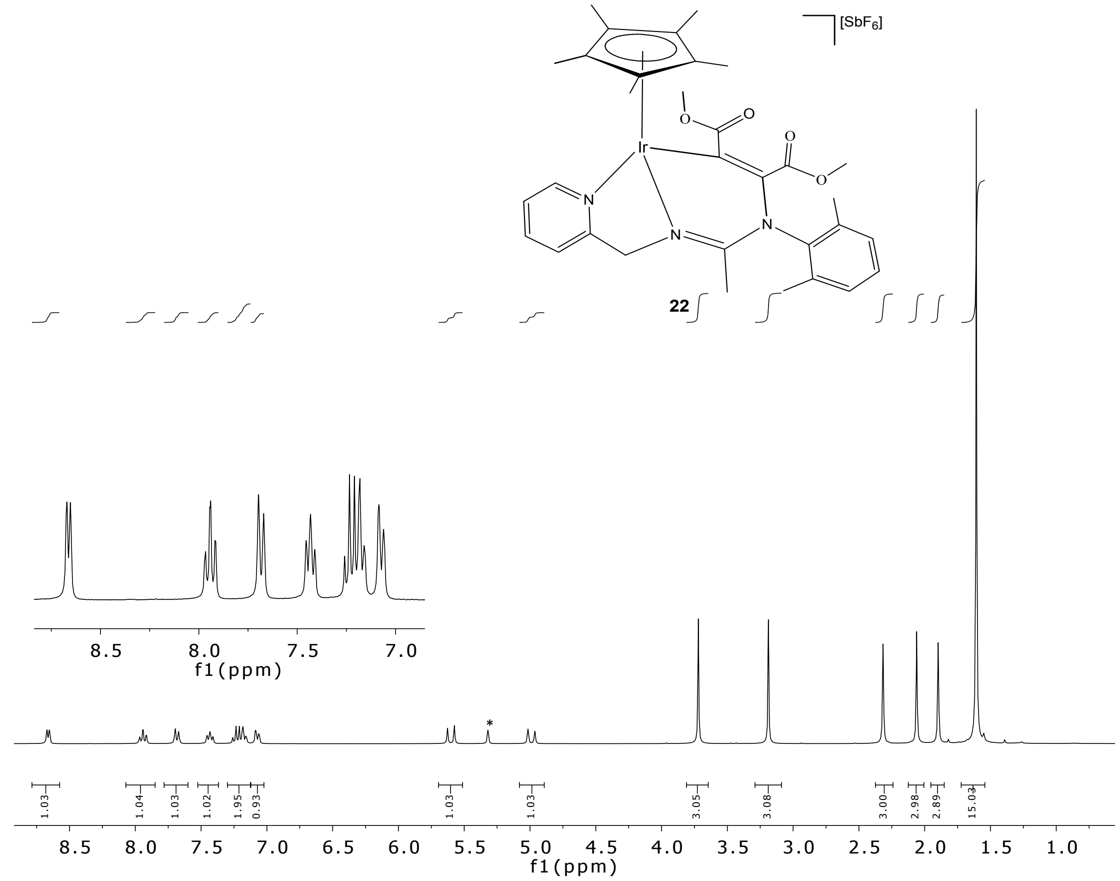

Fig. S40. $^{13}C\{^{1}H\}$-NMR (CD_2Cl_2, RT) spectrum of **22**

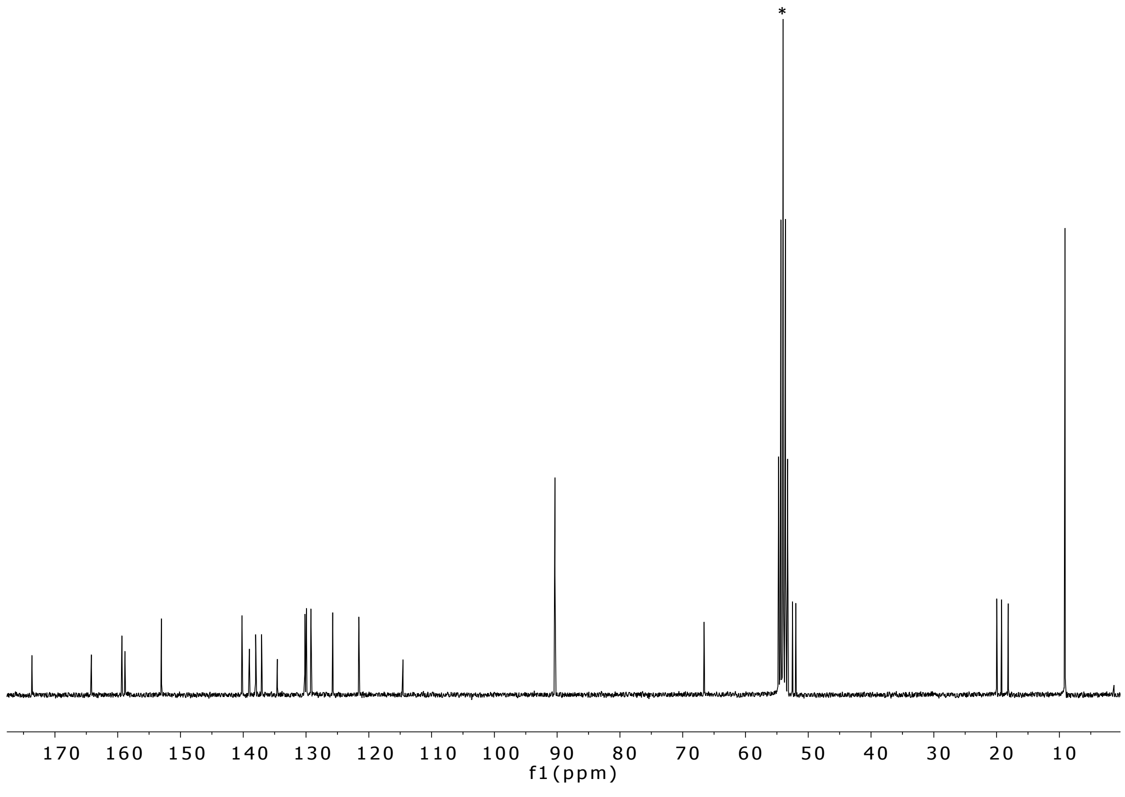

2. Relevant NOE interactions

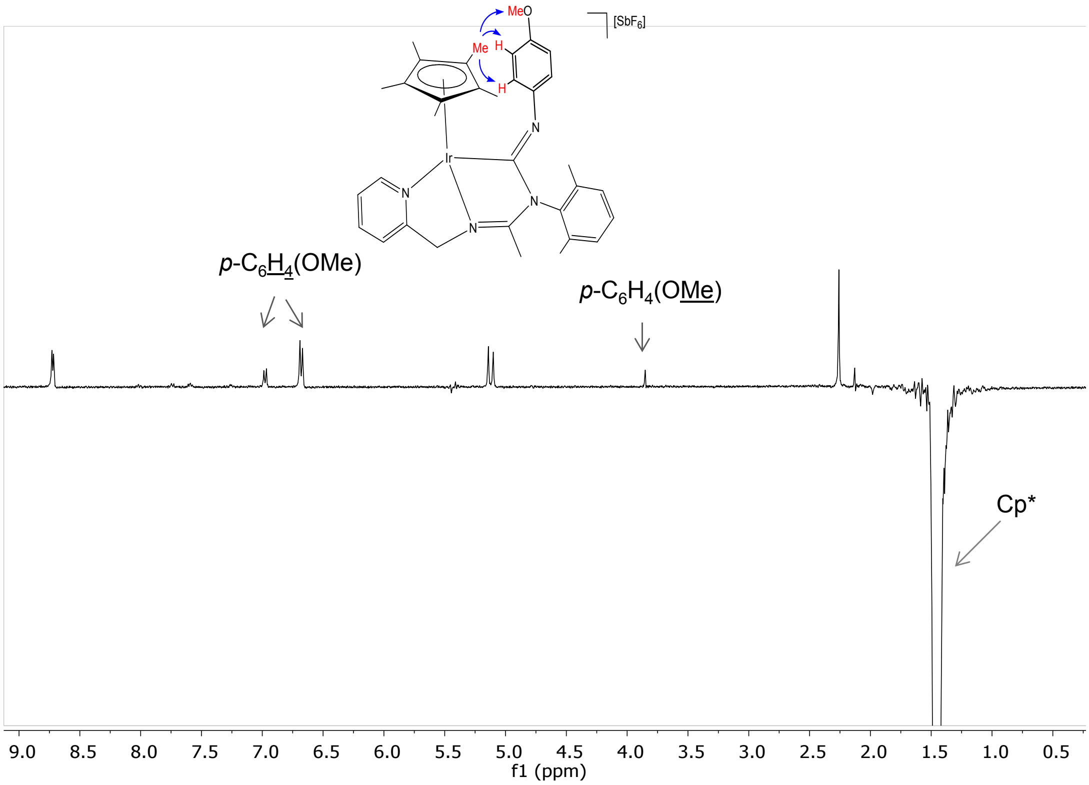

Fig. S41. NOEDIFF spectrum (CD_2Cl_2, RT) of **8** irradiating the Cp* methyl protons.

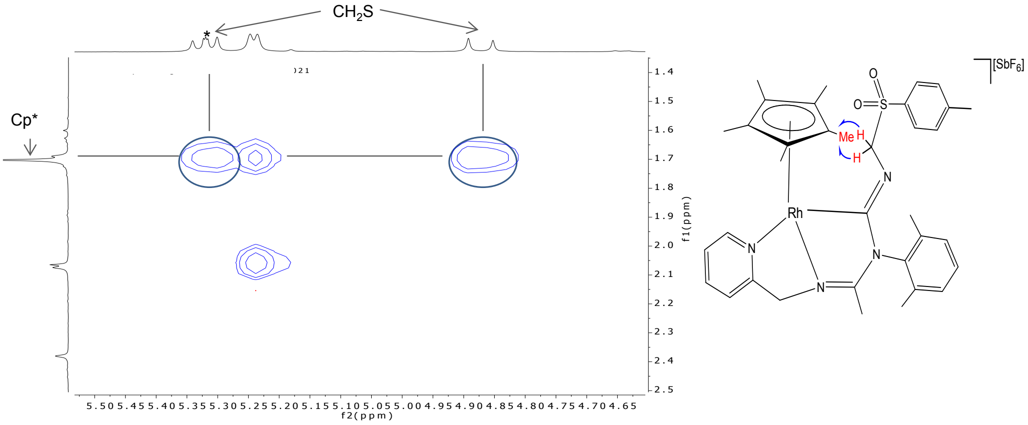

Fig. S42. NOESY fragment (CD_2Cl_2, RT) of **9** showing the contacts CH_2S contacts.

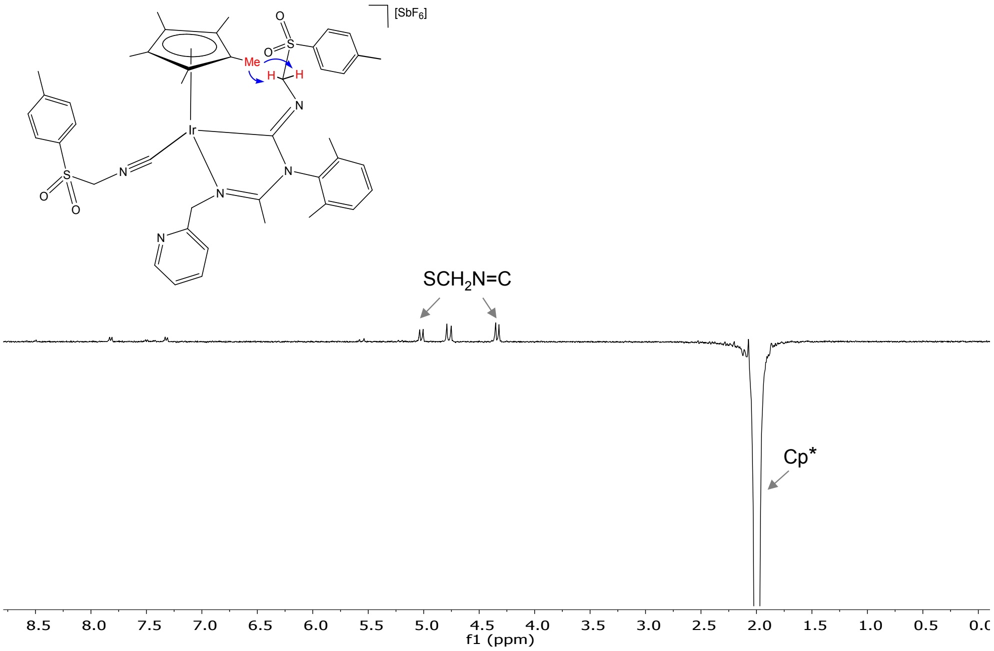

Fig. S43. NOEDIFF spectrum (CD_2Cl_2, RT) of **15** irradiating the Cp* methyl protons.

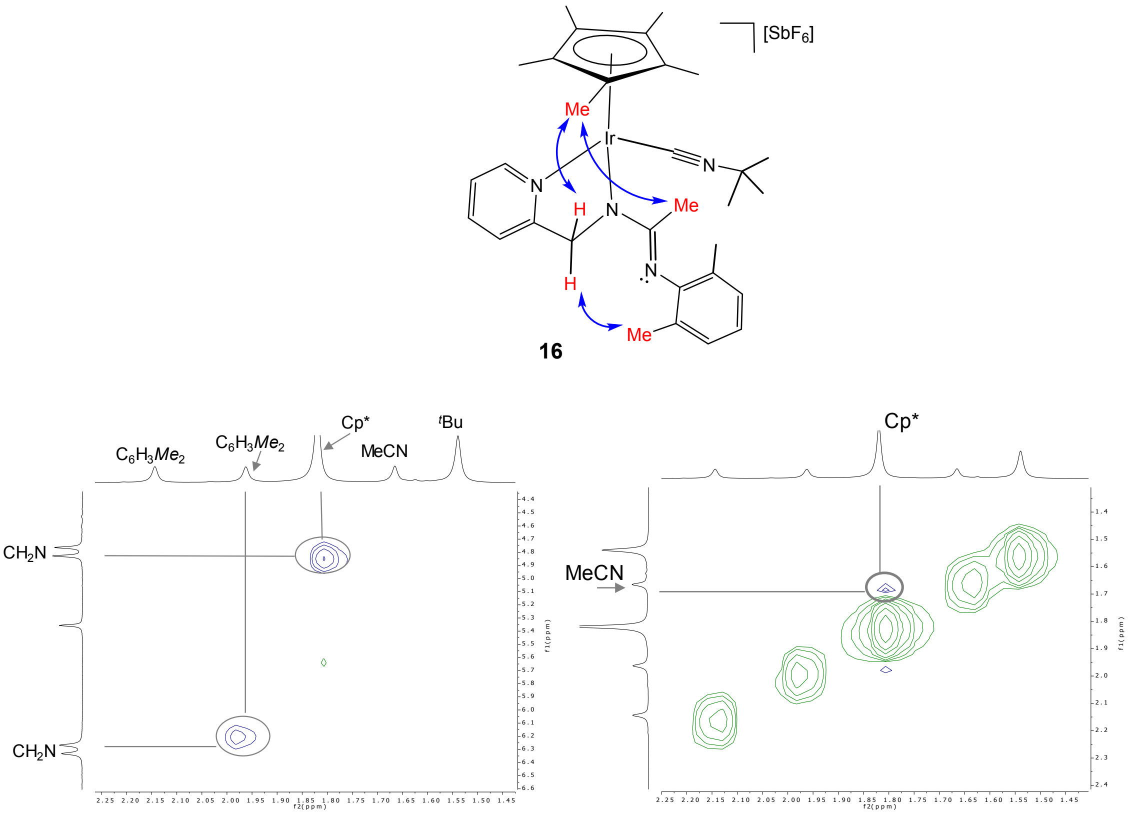

Fig. S44. NOESY fragments (CD_2Cl_2, 233 K) of **16** showing relevant contacts.

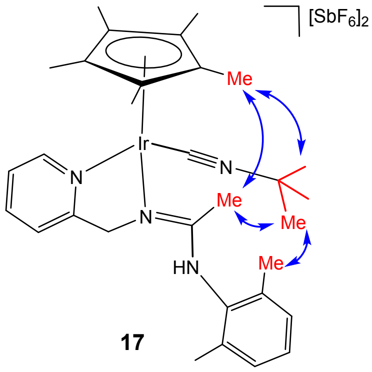

17

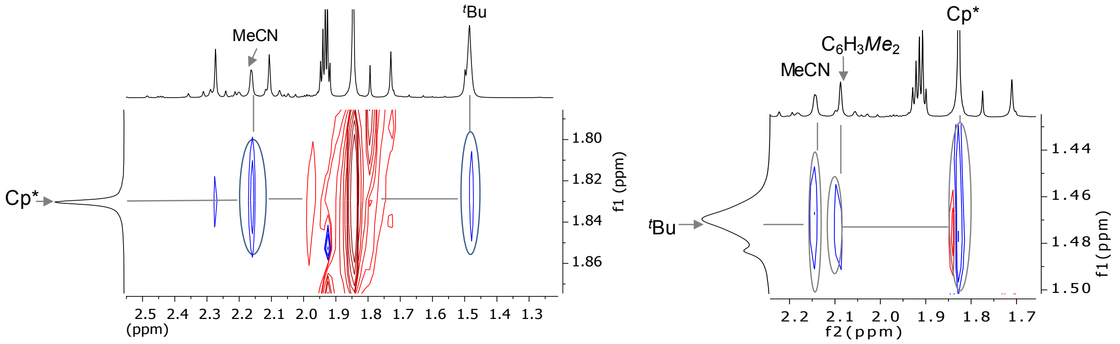

Fig. S45. NOESY fragments (CD_2Cl_2, RT) of **17** showing relevant contacts of Cp* (left) and ^{t}Bu (right).

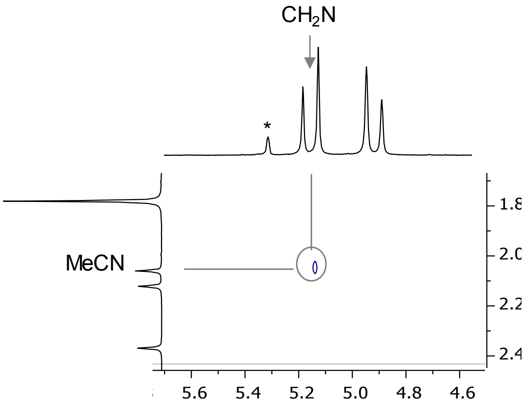

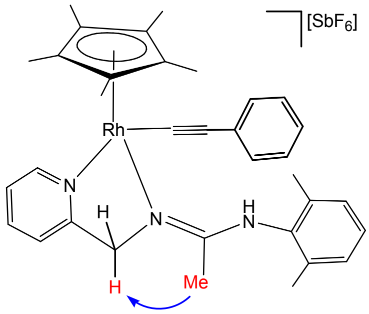

18

Fig. S46. NOESY fragment (CD_2Cl_2, RT) of **18** showing the MeCN↔CH_2N contact.

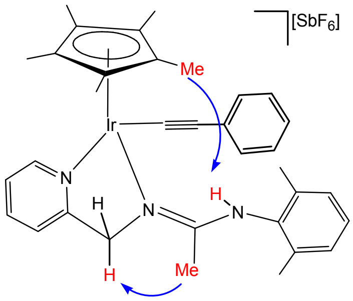

19

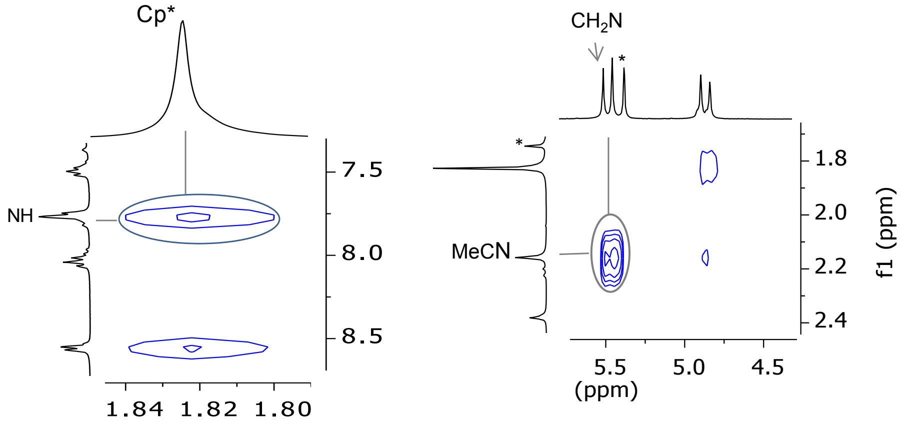

Fig. 47. NOESY fragments (CD_2Cl_2, RT) of **19** showing relevant NH (left) and MeCN (right) contacts.

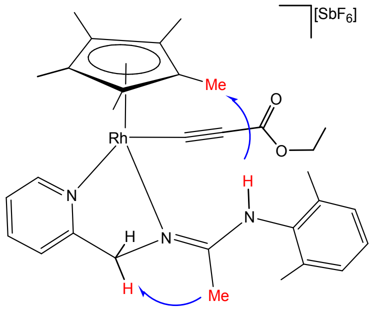

20

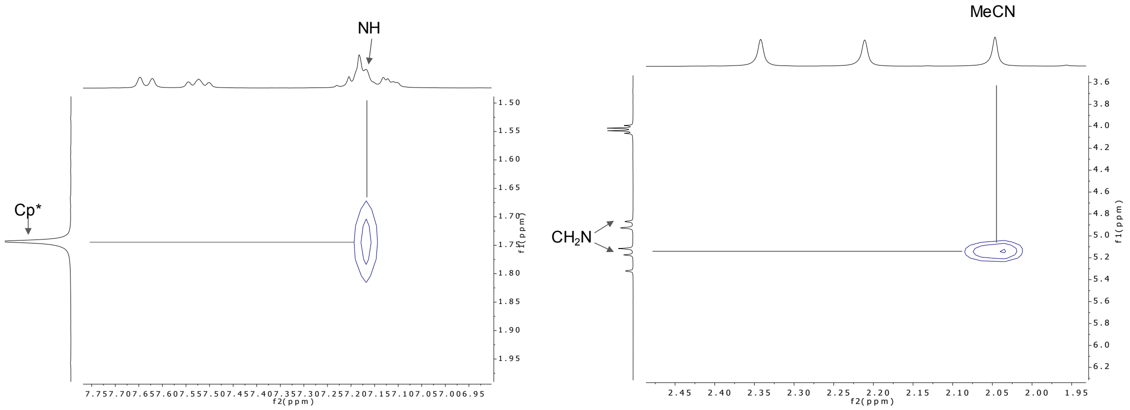

Fig. S48. NOESY fragments (CD_2Cl_2, RT) of **20** showing NH (left) and MeCN (right) contacts.

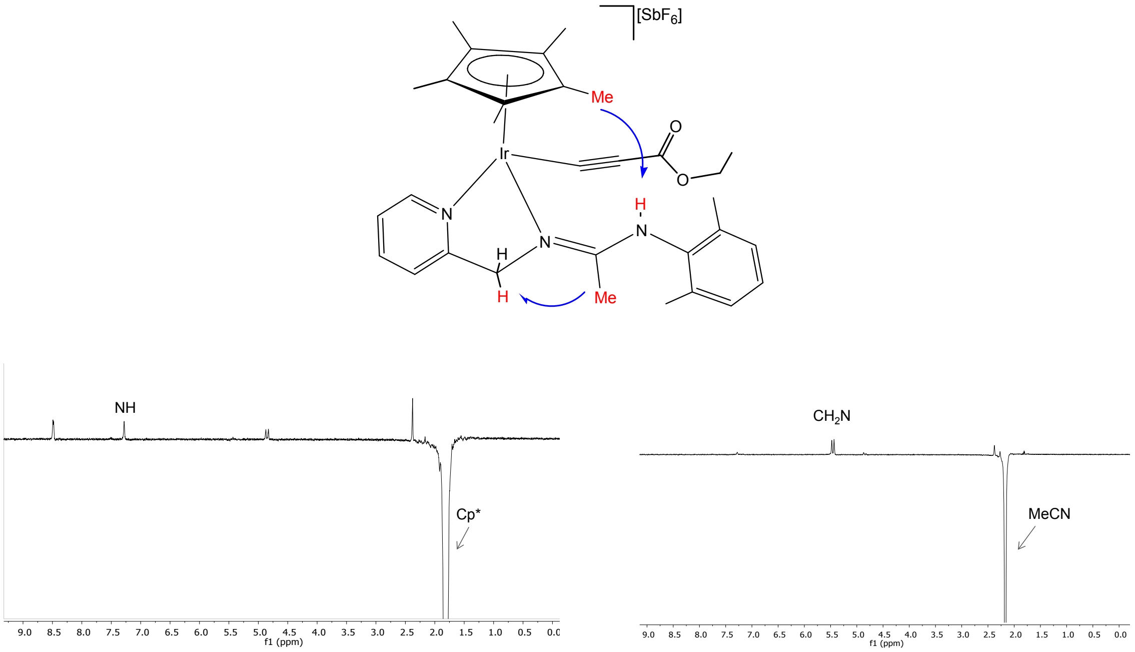

Fig. S49. NOEDIFF spectra (CD_2Cl_2, RT) of **21** irradiating the Cp* (left) and MeCN (right) protons.
